# SARS-CoV-2 receptor ACE2 is upregulated by fatty acids in human MASH

**DOI:** 10.1016/j.jhepr.2023.100936

**Published:** 2023-10-13

**Authors:** Luis Cano, Lise Desquilles, Gevorg Ghukasyan, Gaëlle Angenard, Clémence Landreau, Anne Corlu, Bruno Clément, Bruno Turlin, Eric Le Ferrec, Caroline Aninat, Julie Massart, Orlando Musso

**Affiliations:** 1 INSERM, INRAE, Univ Rennes 1, Nutrition Metabolisms and Cancer, Rennes, France; 2 Univ Rennes 1, CNRS, INSERM, UMS Biosit, Core Facility H2P2, Rennes, France; 3 Univ Rennes 1, INSERM, EHESP, IRSET (Institut de Recherche en Santé Environnement et Travail) UMR_S 1085, Rennes, France

**Keywords:** Metabolism, DC-SIGNR, CLEC4M, TMPRSS2, DPP4, SARS-CoV-2, MAFLD, NAFLD, NASH, Metabolic syndrome, Oleic acid, Stearic acid

## Abstract

**Background & Aims:**

Metabolic dysfunction-associated steatotic liver disease (MASLD) results in steatosis, inflammation (steatohepatitis), and fibrosis. Patients with MASLD more likely develop liver injury in coronavirus disease 2019 (COVID-19), caused by the severe acute respiratory syndrome coronavirus 2 (SARS-CoV-2). As viral RNA has been identified in liver tissues, we studied expression levels and cellular sources of the viral receptor angiotensin-converting enzyme 2 (ACE2) and coreceptors in MASLD and fibroinflammatory liver diseases.

**Methods:**

We built a transcriptomic MASLD meta-dataset (N = 243) to study SARS-CoV-2 receptor expression and verified results in 161 additional cases of fibroinflammatory liver diseases. We assessed the fibroinflammatory microenvironment by deconvoluting immune cell populations. We studied the cellular sources of ACE2 by multiplex immunohistochemistry followed by high-resolution confocal microscopy (N = 9 fatty livers; N = 7 controls), meta-analysis of two single-cell RNA sequencing datasets (N = 5 cirrhotic livers; N = 14 normal livers), and bulk transcriptomics from 745 primary cell samples. *In vitro,* we tested *ACE2* mRNA expression in primary human hepatocytes treated with inflammatory cytokines, bacterial lipopolysaccharides, or long-chain fatty acids.

**Results:**

We detected ACE2 at the apical and basal poles of hepatocyte chords, in CLEC4M^+^ liver sinusoidal endothelial cells, the lumen of ABCC2^+^ bile canaliculi, HepPar-1^+^-TMPRSS2^+^ hepatocytes, cholangiocytes, and CD34^+^ capillary vessels. ACE2 steeply increased between 30 and 50 years of age; was related to liver fat area, inflammation, high immune reactivity, and fibrogenesis; and was upregulated in steatohepatitis. Although *ACE2* mRNA was unmodified in alcoholic or viral hepatitis, it was upregulated in fibroinflammatory livers from overweight patients. *In vitro,* treatment of primary human hepatocytes with inflammatory cytokines alone downregulated but long chain fatty acids upregulated *ACE2* mRNA expression.

**Conclusions:**

Lipid overload in fatty liver disease leads to an increased availability of ACE2 receptors.

**Impact and implications:**

COVID-19 can be a deadly disease in vulnerable individuals. Patients with fatty liver disease are at a higher risk of experiencing severe COVID-19 and liver injury. Recent studies have indicated that one of the reasons for this vulnerability is the presence of a key cell surface protein called ACE2, which serves as the main SARS-CoV-2 virus receptor. We describe the cellular sources of ACE2 in the liver. In patients with fatty liver disease, ACE2 levels increase with age, liver fat content, fibroinflammatory changes, enhanced positive immune checkpoint levels, and innate immune reactivity. Moreover, we show that long chain fatty acids can induce ACE2 expression in primary human hepatocytes. Understanding the cellular sources of ACE2 in the liver and the factors that influence its availability is crucial. This knowledge will guide further research and help protect potentially vulnerable patients through timely vaccination boosters, dietary adjustments, and improved hygiene practices.

## Highlights

• ACE2 is detected at the apical pole of hepatocytes, sinusoidal and capillary endothelial cells, bile canaliculi, and bile ducts.
• In 243 patients with fatty liver disease, ACE2 increased with age, liver fat content, and fibroinflammatory markers.
• ACE2 expression correlated with enhanced innate immune reactivity and positive immune checkpoint modulation.
• ACE2 expression was upregulated in chronic fibroinflammatory liver diseases in overweight patients.
• ACE2 expression was induced by long-chain fatty acids in primary human hepatocytes *in vitro.*

## Graphical abstract

**Figure:**
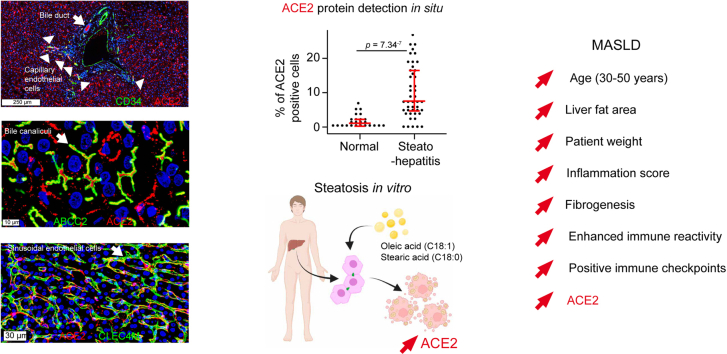

## Introduction

Steatotic liver disease affects 25% of the population, with a prevalence of 1 billion people worldwide. Western diet and lifestyle, and genetic and environmental background lead to common pathological changes, namely, steatosis, inflammation (steatohepatitis), fibrosis, and pre-neoplastic foci.^1^ The diagnoses of non-alcoholic fatty liver disease (NAFLD) and non-alcoholic steatohepatitis (NASH) exclude alcohol as an aetiological agent. However, in everyday clinical practice, steatotic liver disease may coexist with viral hepatitis, autoimmune diseases, and alcohol. Thus, the term *metabolic associated fatty liver disease* was proposed in 2020.^1^^,^^2^ However, a consensus among experts from 56 countries has been reached on the nomenclature for fatty liver disease. The vast majority agreed on the terms metabolic dysfunction-associated steatotic liver disease (MASLD) and metabolic dysfunction-associated steatohepatitis (MASH). These terms were chosen because they are considered less stigmatising and are expected to improve the identification of patients with steatotic liver conditions. MASLD diagnosis is based on steatosis plus at least one of five cardiometabolic criteria, including BMI, insulin resistance, blood pressure, plasma triglycerides, and HDL-cholesterol levels.^3^ MASLD severity is assessed in a continuum across a grading of inflammatory activity and the stage of fibrosis.[1], [2], [3]

Recent studies showed that patients with metabolic syndrome, MASLD, or MASH are at higher risk of liver injury when infected with the severe acute respiratory syndrome coronavirus 2 (SARS-CoV-2), which causes coronavirus disease 2019 (COVID-19).^4^^,^^5^ SARS-CoV-2 is an enveloped (60- to 120-nm particle) RNA betacoronavirus phylogenetically similar to SARS-CoV and the Middle East respiratory syndrome coronavirus (MERS-CoV). Approximately 50% of hospitalised SARS-CoV-2-infected individuals present comorbidities, including hypertension, metabolic syndrome, and coronary heart disease. Patients developing severe disease present acute respiratory distress syndrome, immune dysregulation, and a ‘cytokine storm’ with disseminated intravascular coagulation and blood vessel lesions.^6^

Cell entry of SARS-CoV-2 depends on the binding of the viral spike protein to its specific receptor, namely, the angiotensin-converting enzyme 2 (ACE2), and to co-receptors, such as the serine proteases transmembrane serine protease 2 (TMPRSS2) and FURIN for spike protein fusion^7^^,^^8^ and the C-type lectin domain family 4 member M (CLEC4M) for cell surface adhesion.^9^ In patients dying from COVID-19, viral particles have been detected in hepatocytes,^10^ and viral RNA has been detected in portal vein endothelial cells.^11^ Moreover, HuH7 human hepatocellular carcinoma and HepG2 hepatoblastoma cells express ACE2 and can be infected with SARS-CoV-2 at high titres,^7^ and ACE2 downregulation reduces virus infection in human livers perfused *ex situ*.^12^ However, both the cellular localisation and expression patterns of the SARS-CoV-2 cell receptor ACE2 and co-receptors remain unclear, in particular in MASLD.

ACE2 is a key player of the renin–angiotensin (Ang)–aldosterone system. Renin, which is secreted by the juxtaglomerular cells of the afferent kidney arterioles, cleaves liver-derived angiotensinogen into angiotensin 1 (Ang I), which is hydrolysed into Ang II by ACE1 and exerts potent vasopressor effects. The carboxypeptidase ACE2 cleaves Ang II into Ang_1–7_, which activates the MAS1 proto-oncogene, G protein-coupled receptor (MAS1), leading to smooth muscle relaxation, hypotension, and cardioprotection. ACE2 cleaves other substrates including the inflammatory peptide des-Arg_9_-bradykinin and apelin-13; the latter is involved in glucose uptake and energy metabolism.^13^ Activation of the ACE2/Ang_1–7_/MAS axis downregulates hepatic lipid uptake and lipogenesis, favouring lipid oxidation, and mitochondrial function, improving glucose metabolism^14^^,^^15^ and reducing oxidative stress, inflammation, and liver fibrosis in murine models of NAFLD.^16^

Liver sinusoidal endothelial cells (LSECs) have the highest endocytosis capacity of human cells. Together with Kupffer cells, LSECs remove large amounts of antigens, mediate strong inflammatory responses, and recruit leucocytes, which amplify local inflammation.^17^ LSECs are CD34 negative and CLEC4M positive (also known as L-SIGN, DC-SIGNR), whereas endothelial cells form CD34-positive capillaries. LSECs capture viruses via lectins at their surface and, in turn, can transfer them to hepatocytes.^18^ CLEC4M recognises a wide range of pathogens, among which are SARS-CoV-2, HCV, and Ebola virus.^9^ In SARS-CoV, CLEC4M recognises N-glycans on the spike protein and transfers the virus to permissive cells.^19^

We searched for the expression and localisation of ACE2, TMPRSS2, and CLEC4M in normal human liver and MASLD. To this end, we constructed a transcriptomic fatty liver disease meta-dataset of 243 samples including normal liver, steatosis, steatohepatitis, and MASH. To assess whether the results were specific to MASLD or related to unspecific fibroinflammatory liver changes, we explored five additional transcriptomic datasets totalling 161 patients with fibroinflammatory liver diseases. Receptor and co-receptor localisation and cellular sources were studied by high-resolution confocal microscopy scanning after multiplex immunohistochemistry in seven histologically normal liver controls and nine fatty liver disease cases. Cellular sources were also explored by meta-analysis of single-cell RNA sequencing data from two human studies^20^^,^^21^ totalling 14 normal and five cirrhotic livers and bulk transcriptomics from 745 primary cell samples.^22^ Regulation of ACE2 expression was studied on primary human hepatocyte cultures treated with inflammatory cytokines, bacterial lipopolysaccharides (LPS), and long-chain fatty acids.

We show that steatohepatitis was consistently associated with increased expression of ACE2 in LSECs and hepatocytes. In 243 patients with fatty liver disease, *ACE2* mRNA expression progressively increased with age but plateaued between 50 and 80 years and correlated with liver fat area, inflammation, enhanced immune reactivity, and fibrogenesis. In 41 patients with fibroinflammatory liver diseases, *ACE2* expression was upregulated in a background of patient overweight. These findings were supported by upregulation of *ACE2* expression by long-chain fatty acids in an *in vitro* model of steatohepatitis in primary human hepatocytes. By contrast, inflammatory cytokines alone were unable to upregulate *ACE2* expression. Altogether, these data suggest that ACE2 upregulation is a physiological compensatory mechanism in response to a liver overload in fatty acids. However, the co-occurrence of high immune reactivity and increased availability of ACE2 receptors in fatty liver disease may promote viral infection, amplified inflammation, and patient decompensation.

## Materials and methods

### Patients, samples, and datasets

For fatty liver disease samples, routine formalin-fixed, paraffin-embedded non-tumour liver tissues were obtained from the Anatomic Pathology Laboratory, Rennes University Hospital, after informed consent of nine patients with MASLD and five control patients undergoing partial hepatectomy (Table S1). Sample collection, exploitation, and data analysis were performed within the framework of the INSERM’s Institutional Review Board Approval No. 19-630. Routine cases were reviewed and selected by an experienced liver pathologist (BT). Digital slides were viewed using NDP.view (Hamamatsu Photonics). The signal was independently read by two observers (LC and OM), who were blinded to any information. Discrepancies were resolved by consensus reading. Scoring of MASLD and MASH activities was done as described on a four-point scale.^23^^,^^24^

A human fatty liver disease transcriptomic meta-dataset of 243 samples was constructed by merging three microarray datasets: GSE33814^25^ (12 normal livers, 19 steatoses, and 12 steatohepatitides), GSE48452^26^ (14 normal livers, 27 livers from obese patients, 14 steatoses, and 18 non-alcoholic steatohepatitides [MASHs]), and GSE83452^27^ (231 samples from which 126 MASHs were extracted for study). Altogether, the meta-dataset includes 27 normal livers, 27 livers from obese patients, 33 steatoses, 12 steatohepatitides, and 144 MASHs. Batch effect was removed using the ComBat algorithm (*sva* R package), as we previously described.^28^ Raw expression data were quantile-normalised and log_2_-transformed.

### Antibodies and oligonucleotides

Primary and secondary antibodies, antigen retrieval procedures, stainer kits, signal amplification methods, incubation conditions, and dilutions are summarised in Table S2. Real-time PCR primers are detailed in Table S3.

See Supplementary information for a detailed description of standard benchwork procedures such as human primary hepatocyte isolation and culture, immunohistochemistry, cell culture and treatments, RNA extraction and real-time PCR, deconvolution of immune cell populations, and statistical analyses.

## Results

### High expression of ACE2 protein in liver sinusoids in steatohepatitis

**Fig. 1:**
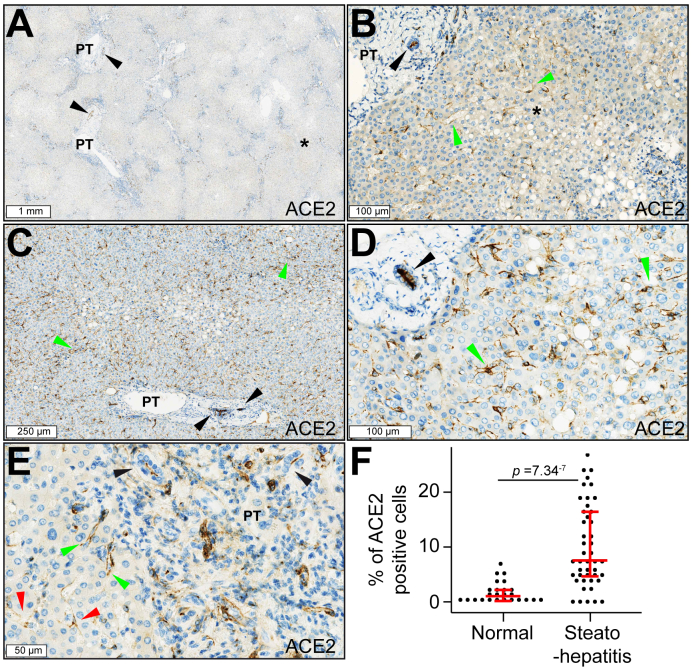
Increased expression of ACE2 in steatohepatitis. (A) ACE2-positive bile ducts (brown signal, arrowheads), enlarged PTs with inflammatory infiltrates, and incomplete fibrous septa outlining parenchymal nodules with variable levels of ACE2 staining (asterisk). (B) Steatohepatitis with predominantly ACE2-positive hepatocytes: ACE2-positive bile ducts (black arrowhead), macrovesicular steatosis with predominantly ACE2-positive hepatocytes (asterisk), and sparsely ACE2-positive sinusoidal cells (green arrowheads). (C and D) Macrovesicular steatosis with predominantly ACE2-positive staining in sinusoidal cells (green arrowheads) and bile ducts (black arrowheads). (E) PT with inflammatory infiltration and ductular reaction (black arrowheads). ACE2-positive cord-like structures resembling blood vessels (green arrowheads) are seen at the interface between the limiting plate and the parenchyma. Sinusoids are lined by ACE2-positive cells (red arrowheads). Digital slides were acquired in a microscope scanner using a 40× objective. (F) Percentage of ACE2-positive cells in normal liver controls (n = 5) and steatohepatitis (n = 9). From each patient, five ACE2-positive 1-mm^2^ hotspots were selected for image analysis. Each dot represents one hotspot; whisker bars show median plus first and third quartiles. The Mann–Whitney *U* test was used to assess statistical significance. Clinical data, and MASLD and MASH scores are shown in Table S1. ACE2, angiotensin-converting enzyme 2; PT, portal tract.

We previously showed that ACE2 is an attribute of normal adult hepatocyte identity.^29^ Recent studies consistently showed that human hepatocytes^10^^,^^12^^,^^15^^,^^30^ and cholangiocytes^12^ can be infected with SARS-CoV-2. Of note, when human cholangiocyte organoids lose their identity *in vitro,* they no longer express ACE2.^12^ In fatty liver disease, the mRNA expression of *ACE2* and that of the SARS-CoV-2 co-receptor *TMPRSS2* are upregulated.^31^^,^^32^ However, their precise cellular sources and the factors that trigger their upregulation in the clinical setting remain elusive. By immunohistochemistry, the primary antibodies and protocols used (Table S2) confirmed the expected high levels of ACE2 in the human kidney (Fig. S1A), the intestine (Fig. S1B), and cholangiocytes (Fig. 1A–D) and of TMPRSS2 in the human prostate (Fig. S1C and D). Of note, our analysis of a transcriptomic microarray meta-dataset assembling 745 primary cell samples from more than 100 studies^22^ revealed that *ACE2* and *TMPRSS2* mRNAs were detected in hepatocytes and the bronchial epithelium at similar levels (Fig. S2A). These findings were consistent with the reported detection of ACE2 protein in human livers^33^ and the detection of low but homogenously distributed ACE2 enzymatic activity by *in situ* autoradiography in normal rat livers.^34^

**Fig. 2:**
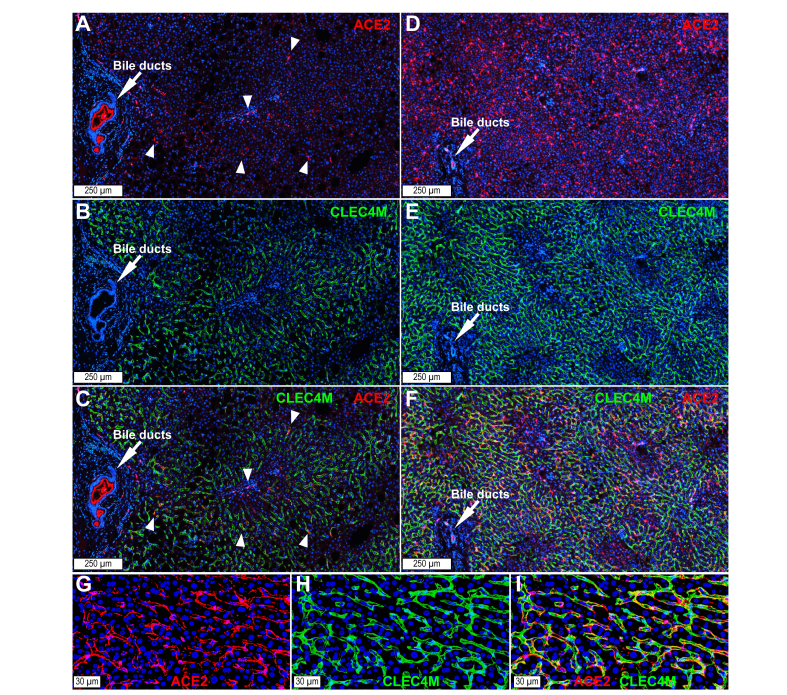
ACE2 in sinusoidal endothelial cells in (A–C) normal liver and (D–F) steatohepatitis. ACE2-positive signal (red) colocalises with the liver sinusoidal **endothelial cell marker CLEC4M (also known as DC-SIGNR) (green) lining the** sinusoidal endothelium. In normal liver, sparse ACE2-positive sinusoidal cell spots are seen at low power (white arrowheads). Bile ducts show strong ACE2 signal (white arrows). In steatohepatitis, a high density of ACE2-positive sinusoidal cells is observed. (G–I) Higher power view of ACE2 expression in the sinusoidal endothelium in steatohepatitis. Nuclei are seen in blue (DAPI). Digital slides were acquired using a 40× objective in a confocal scanner. The images show a Z-stack of four 500-nm focusing steps. ACE2, angiotensin-converting enzyme 2; CLEC4M, C-type lectin domain family 4 member M.

We analysed ACE2 and TMPRSS2 expression and localisation in nine cases of fatty liver disease and five controls by immunohistochemistry. Available clinical and biological data, as well as scores for steatosis, ballooning, inflammation, MASLD, and MASH are shown in Table S1. In normal livers, we did not detect TMPRSS2 protein (not shown) but confirmed ACE2 in bile ducts, capillary blood vessels within portal tracts, and sinusoidal cells (Fig. S2B and C). In steatohepatitis, we detected ACE2 (Fig. 1A and B) in hepatocytes within parenchymal nodules outlined by inflammatory infiltrates. ACE2 was also detected in sinusoidal cells (Fig. 1C and D) and in capillary blood vessels within inflamed portal tracts (Fig. 1E). Image analysis revealed higher levels of ACE2 in nine cases of fatty liver disease *vs.* five controls (Fig. 1F). Although TMPRSS2 protein was detected at lower levels than was ACE2, both were co-expressed in hepatocytes in steatohepatitis samples (Fig. 2D–G).

### Detection of ACE2 in sinusoidal endothelial cells and bile canaliculi in steatohepatitis

**Fig. 3:**
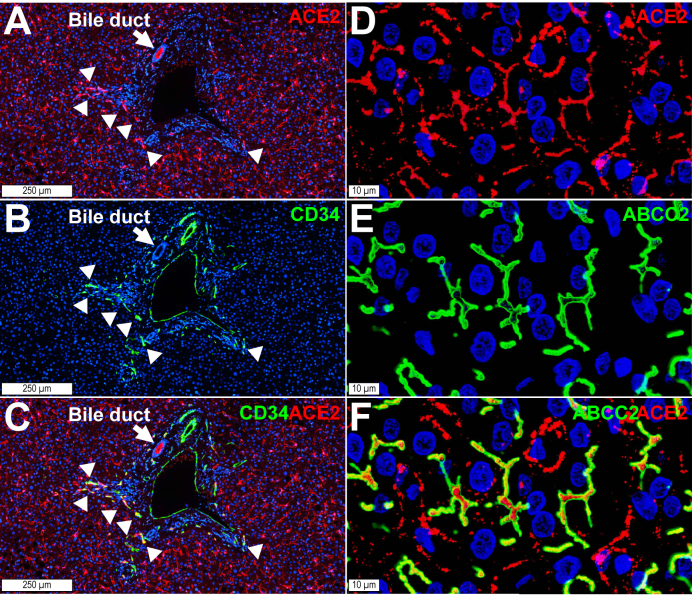
ACE2 in (A–C) CD34-positive capillary endothelial cells and (D–F) bile canaliculi. (A–C) Steatohepatitis. CD34-positive endothelial cells (green, arrowheads) within portal tracts and at the interface between the limiting plate and the parenchyma are ACE2-positive (red, arrowheads). (D–F) Bile canaliculi, identified with the specific marker ABCC2 (also known as MRP2) (green), contain ACE2 (red). Digital slides were acquired using a 40× objective in a confocal scanner. The image shows a Z-stack of four 500-nm focusing steps. ABCC2, ATP binding cassette subfamily C member 2; ACE2, angiotensin-converting enzyme 2; MRP2, multidrug resistance-associated protein 2.

Immunohistochemistry confirmed colocalisation of ACE2 and the LSEC marker CLEC4M (also known as DC-SIGNR) in normal liver and steatohepatitis (Fig. 2A–I). Colocalisation with CD34-confirmed ACE2 in capillary endothelial cells within inflamed portal tracts (Fig. 3A–C). Moreover, ACE2 colocalised with the bile canaliculi marker ATP binding cassette subfamily C member 2 (ABCC2, also known as multidrug resistance-associated protein 2 [MRP2]) (Fig. 3D–F), which is consistent with the abundance of ACE2 in the human bile proteome.^35^ Altogether, these findings showed that the basal and apical poles of hepatocyte chords display ACE2 receptors in patients with steatohepatitis.

We also searched for ACE2 protein expression in resident liver immune cells. ACE2 was not detected at significant levels in CD45^+^ leucocytes (Fig. S3). Frequent images of CD68^+^ Kupffer cell membrane extensions in close contact with ACE2^+^ endothelial cells and thus overlapping within the same focal plane were seen (Fig. S4A and D). In this case, digital deconvolution of high-power confocal images across 500-nm steps in the Z axis showed that CD68^+^ Kupffer cell membrane extensions lined ACE2^+^ sinusoidal endothelial cells (Fig. S4E and G). ACE2 was not detected at significant levels in CD3^+^ T lymphocytes or in Actin alpha 2, smooth muscle (ACTA2)-positive myofibroblasts (Fig. S5).

Meta-analysis of single-cell sequencing data of histologically normal liver samples from nine patients^20^ confirmed that *ACE2* mRNA levels are globally low in all cell populations tested. Scattered *ACE2-*positive cells were detected in clusters containing sinusoidal endothelial cells, periportal and perivenous hepatocytes, and cholangiocytes (Fig. S6). In addition, single-cell RNA sequencing data of five normal and five cirrhotic human livers (including NAFLD, alcohol-related liver disease, and primary biliary cirrhosis)^21^ revealed low levels of *ACE2* and *TMPRSS2* in hepatocytes, cholangiocytes, and mesenchymal and endothelial cells (Fig. S7A–C). Taken together, our experimental and meta-analyses data showed low general levels of *ACE2* and *TMPRSS2* in all liver cell subsets tested, with upregulation of *ACE2* mRNA^31^^,^^32^ and protein in fatty liver disease. ACE2 was located mainly at the sinusoidal and biliary poles of hepatocyte cords. In summary, cholangiocytes, sinusoidal endothelial cells, and hepatocytes are the major cell sources of ACE2 protein in the liver. Resident liver immune cells do not appear to express detectable levels of ACE2 protein in steatohepatitis, as observed using the highly sensitive tyramide signal amplification method.

### Liver *ACE2* mRNA expression increases proportionally with age, inflammation, and fibrogenesis in patients with fatty liver disease

**Fig. 4:**
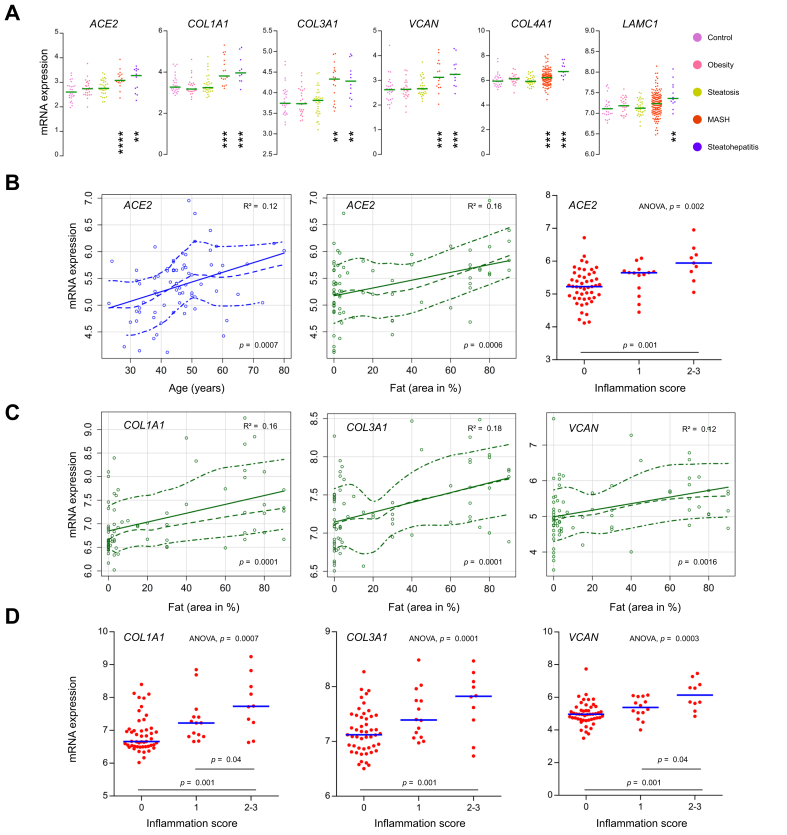
*ACE2* mRNA expression increases proportionally with age, fat content, inflammation, and fibrogenesis in 243 patients with fatty liver disease. Meta-dataset constructed from three independent datasets (GSE33814, GSE48452, and GSE83452). After quantile normalisation and batch effect suppression, it was composed 243 liver samples (27 normal controls, 27 obese, 33 steatoses, 144 MASHs, and 12 steatohepatitides) and 20,941 different RNA transcripts. (A) *ACE2* is associated with the fibrogenesis markers *COL1A1*, *COL3A1*, and *VCAN,* and basement membrane remodelling markers *COL4A1* and *LAMC1*. ANOVA followed by Tukey’s *post hoc* test was used (∗∗*p* <0.01, ∗∗∗*p* <0.001, ∗∗∗∗*p* <0.0001; asterisks compare pathological conditions with controls). For each gene, the numbers of available observations are indicated in Table S4. (B) *ACE2* mRNA expression is associated with increasing age, liver biopsy fat area, and inflammation score in 72 patients from the GSE48452 dataset. (C and D) The fibrogenesis markers *COL1A1*, *COL3A1*, and *VCAN* are associated with increasing liver fat area (C) and inflammation (D). ACE2, angiotensin-converting enzyme 2; MASH, metabolic dysfunction-associated steatohepatitis.

We assessed the regulation of *ACE2* and *TMPRSS2* expression in MASLD after merging three independent microarray datasets[25], [26], [27] into a meta-dataset consisting of 243 liver samples and 20,941 mRNA transcripts. Batch effect correction and normalisation were applied to minimise experimental bias (Fig. S8A). The expression of *TMPRSS2* remained unchanged in MASLD, whereas the MERS-CoV receptor dipeptidyl peptidase 4 (*DPP4*) and the SARS-CoV-2 coreceptor CLEC4M were lower in steatosis and steatohepatitis, with respect to controls (Fig. S8B). By contrast, *ACE2* mRNA reached higher levels in MASH and steatohepatitis (Fig. 4A). An expression profile similar to that of *ACE2* was detected for the extracellular matrix components *COL1A1, COL3A1, VCAN, COL4A1*, *and LAMC1* (Fig. 4A), which suggests that *ACE2* expression is upregulated concomitantly with fibrogenesis. *ACE2* expression was also correlated with patient age, fat area, and inflammation score in liver biopsies (Fig. 4B). Remarkably, the ACE2/age relationship drew a steep slope between 30 and 50 years of age and plateaued afterwards. Fat area was also related to the expression of the major extracellular matrix components *COL1A1, COL3A1*, and *VCAN* (Fig. 4C). In turn, the mRNA level of these extracellular matrix components was directly proportional to the inflammation scores (Fig. 4D). Altogether, these data reveal upregulation of *ACE2* mRNA expression in MASLD.

### The expression of *ACE2*, *TMPRSS2*, and *DPP4* mRNAs in fatty liver tissues correlates with markers of immune cell infiltration

The absence of detectable ACE2 protein in liver immune cells by immunohistochemistry, despite the use of the highly sensitive tyramide-based signal amplification technology, led us to search for mRNA expression of *ACE2*, *TMPRSS2*, and *DPP4* in immune cell subsets isolated from circulating leucocytes. To this end, we analysed the *Immune Response in Silico* (IRiS) microarray dataset, composed of 228 immune cell samples that had been isolated by cell-specific antibody pull-down before transcriptomic analyses.^36^ First, we confirmed detection of immune cell subset marker mRNAs specific to the cell types analysed (Fig. S9). Next, we detected *ACE2* in B lymphocytes (naive B cells, and IgA and IgM/G memory and plasma cells) and neutrophils. In turn, *TMPRSS2* was detected at variable levels in all cell types except in plasma cells. By contrast, DPP4 was restricted to CD4^+^ T, CD8^+^ T, and natural killer lymphocytes (Fig. S10A). Of note, although *ACE2* and *TMPRSS2* mRNAs were detected in purified immune cells from circulating leucocytes, the mRNA abundance was 100-fold lower than that of cell surface cluster differentiation immune cell markers (compare Figs. S9 and S10A).

To infer whether expression of *ACE2, TMPRSS2,* and *DPP4* mRNAs in fatty liver disease tissues was associated with inflammatory infiltration, we explored the GSE33814 dataset composed of 13 normal livers, 19 steatoses, and 12 steatohepatitides^25^ and searched for correlations with immune cell subset-specific markers, according to the IRiS immune cell repository^36^ (Fig. S10B). Liver *ACE2* mRNA expression was most highly correlated with liver infiltration by T and B lymphocytes, macrophages, and dendritic cells. *TMPRSS2* mRNA was associated with macrophage and neutrophil markers, whereas *DPP4* was associated with macrophage, neutrophil, and T-cell markers. Validating the above findings, we found correlation of *ACE2* with T-cell, B-cell, and dendritic cell markers; *TMPRSS2* with macrophage and neutrophil markers; and *DPP4* with T-cell markers in an independent dataset (GSE48452; Fig. S11).^26^

These results show that upregulation of *ACE, TMPRSS2*, and *DPP4* mRNA expression correlates with immune cell infiltration in steatohepatitis. They match with the higher levels of ACE2 protein expression seen in steatohepatitis with respect to control livers and with the association of *ACE2* mRNA levels with fibroinflammation in fatty liver disease. Taken together, these results show an association between the upregulation of the expression of SARS-CoV-2 entry points in the liver and inflammation. Although the virus could use circulating immune cells as shuttles to colonise the liver, resident liver immune cells do not exhibit significant levels of the major SARS-CoV-2 receptor ACE2.

### Steatohepatitis shows enhanced immune reactivity

**Fig. 5:**
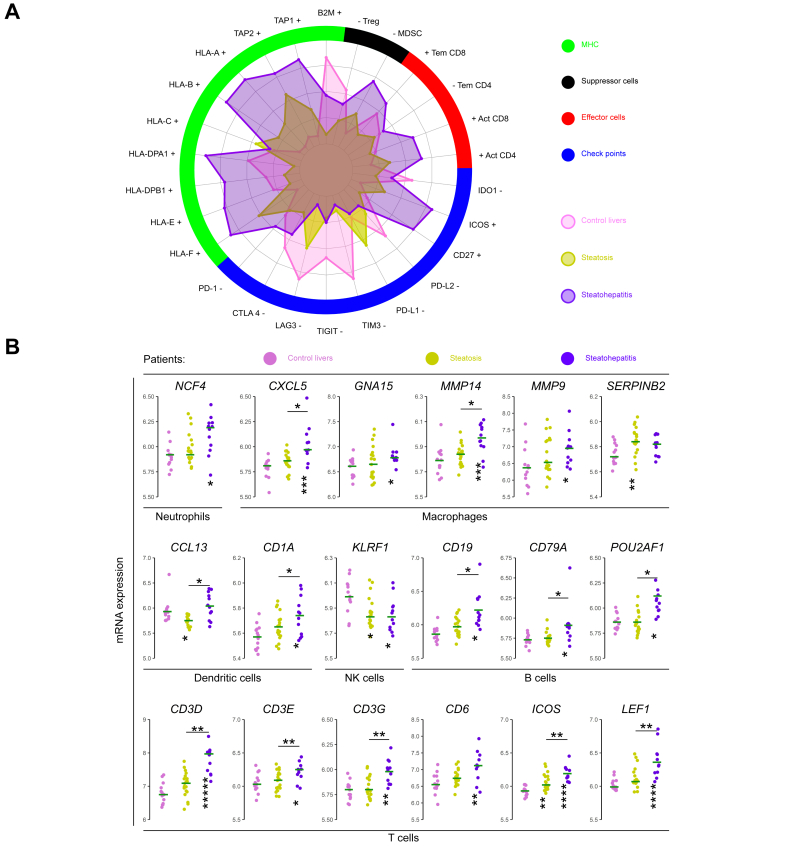
Enhanced immune reactivity in steatohepatitis: antigen presentation, CD4 T-cell activation, and checkpoint modulation. (A) Immune cell subsets in 44 human liver samples from the GSE33814 dataset (13 controls, 19 steatoses, and 12 steatohepatitides) are classified into four immunogenicity functional families: MHC, effector cells, suppressor cells, and checkpoints. Enhancement and suppression of immune reactivity are indicated by^+^ and ^−^, respectively. Parameters’ full names and gene symbols are indicated in Table S5. Steatohepatitis shows enhancement of MHC *(HLA-F*, *HLA-DPA1*, *HLA-B*, *HLA-A*, and *TAP2*) and two positive immunomodulators (*CD27* and *ICOS*), with moderate activation of CD4 T cells and downregulation of the checkpoint inhibitor *TIGIT.* Statistical significances are shown in Table S6. (B) Fatty liver disease samples from the GSE33814 dataset show increased expression of molecular markers for neutrophils, macrophages, dendritic cells, NK cells, and B and T cells. Decreased expression of *KLRF1* indicates NK cell activation.^36^ ANOVA was followed by Tukey’s *post hoc* test to assess the statistical significance of the difference between *control* and *steatosis* or *steatohepatitis* (∗*p* <0.05, ∗∗*p* <0.01, ∗∗∗*p* <0.001, ∗∗∗∗*p <0.0001*). MHC, major histocompatibility complex; NK, natural killer.

To appraise the functional significance of immune cell infiltrates in MASLD, we constructed immunophenograms *(i.e.* phenetic diagrams) that classify immune functions into four families, namely, major histocompatibility complex, effector cells, suppressor cells, and checkpoint inhibitors and immune modulators (Fig. 5A), applying deconvolution algorithms.^37^ Immunophenograms were obtained from 13 controls, 19 steatoses, and 12 steatohepatitides (Fig. S12) from the GSE33814 transcriptomic dataset.^25^ Data are summarised in a spider web chart (Fig. 5A). We observed enhanced immune reactivity in steatohepatitis, with increase in major histocompatibility complex markers involved in antigen processing and presentation (*HLA-F*, *HLA-DPA1*, *HLA-B*, *HLA-A*, and *TAP2*) and positive immunomodulation (*CD27* and *ICOS*), with downregulation of the negative checkpoint *TIGIT* and activation of CD4 T cells. We confirmed these data using two different approaches. First, using the IRiS tool,^36^ we found that steatohepatitides were enriched in neutrophils, macrophages, dendritic cells, natural killer lymphocytes, B lymphocytes, and T lymphocytes (Fig. 5B). Second, we constructed an unsupervised gene co-expression network to identify densely connected sub-networks or modules relating biological functions with the patient groups. Applying weighted gene co-expression network analysis to the meta-dataset consisting of 243 human liver samples, we identified three modules highly associated with steatohepatitis (Figs. S13–16 and Table S7). Gene ontology analysis confirmed functions involved in antigen processing and presentation, lymphocyte chemotaxis, inflammation, fibrogenesis, and typical hepatocyte metabolism. These findings confirm an enhanced immune reactivity in steatohepatitis leading to a chronic fibroinflammatory microenvironment.

### *ACE2* expression is upregulated in chronic fibroinflammatory liver diseases in a context of patient overweight

We tested whether the upregulation of ACE2 was specific to steatohepatitis or whether this was also seen in other fibroinflammatory liver diseases. Analysis of four transcriptomic datasets totalling 120 patients did not reveal significant *ACE2* mRNA changes in the liver in alcoholic hepatitis (GSE28619), blood mononuclear cells (GSE119117), and the liver in HCV infection (GSE48445) or in the liver in HBV infection (GSE54747), except that treatment of patients with HCV with pegylated interferon alpha 2 (IFNA2) was associated with decreased *ACE2* expression with respect to untreated patients (Fig. S17).

We then asked whether recombinant cytokines could upregulate *ACE2* mRNA expression in hepatocytes. Isolation of primary normal human hepatocytes from three patients followed by *in vitro* treatment with IL-6, IL-1B, TNFA, or LPS decreased *ACE2* mRNA expression by several folds, without significant changes in *TMPRSS2* expression. In turn, IL-1B, TNFA and LPS decreased, but IL6 increased *FURIN* mRNA levels. (Fig. S18). These findings are consistent with downregulation of or no effect on *ACE2* mRNA expression of IL-1B, IL-6, IL-10, IL-18, or IFNA2 in combination with IL-1B, IL-6, and TNFA.^38^ They also agree with the notion that inflammatory cytokines lead to a loss of the differentiated hepatocyte identity.^39^ Of note, as we previously showed, *ACE2* is preferentially expressed in differentiated hepatocytes.^29^

**Fig. 6:**
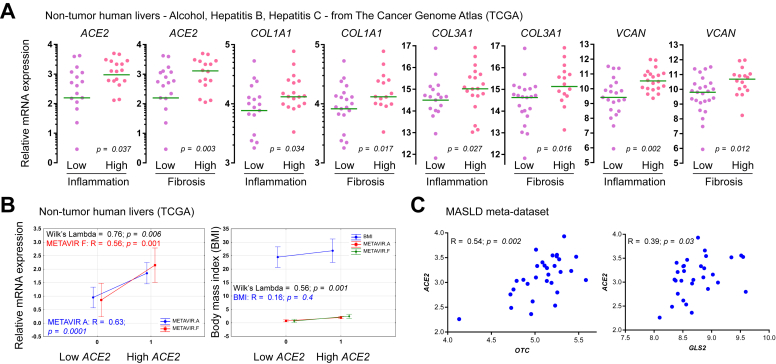
*ACE2* mRNA expression is upregulated in chronic fibroinflammatory liver diseases resulting from alcohol abuse or viral hepatitis in a context of patient overweight. (A) *ACE2* in 41 non-tumour livers from TCGA dataset according to the grading of inflammatory activity and fibrosis, as assessed by METAVIR scoring. Inflammation scores: *low* (0–1) and *high* (2–3). Fibrosis scores: *low* (0–1–2) and *high* (3–4). BMI was available for 30/41 patients; 60% of patients (18/30) had a BMI >23 (overweight). The indicated markers of fibrogenesis correlate with *ACE2* expression. Case scoring is shown in Table S8. Statistical significance of the differences between *low* and *high* were assessed using the Mann–Whitney *U* test. (B) Discriminant function analysis shows the percent of variance in *ACE2* mRNA levels explained by fibrosis (*METAVIR F*), inflammation (*METAVIR A*)*,* and BMI in 41 patients. Smaller values of Wilk’s lambda indicate greater discriminatory ability of the function. Inclusion of BMI increases the discriminatory ability of the model. (C) *ACE2* mRNA levels are correlated with the markers of hepatocyte metabolism *OTC* and *GLS2* in 30 patients with steatohepatitis. ACE2, angiotensin-converting enzyme 2; MASLD, metabolic dysfunction-associated steatotic liver disease; TCGA, The Cancer Genome Atlas.

Treatment of human microvascular endothelial cells with IL-6, IL-1B, TNFA, or LPS did not significantly change *ACE2*, *TMPRSS2*, or *FURIN* mRNA expression (Fig. S19). Thus, we hypothesised that ACE2 upregulation could result from the synergy between chronic inflammation and fibrogenesis in a context of patient overweight. We analysed the mRNA expression of *ACE2* and the markers of fibrogenesis *COL1A1, COL3A1,* and *VCAN* in 41 non-tumour livers from TCGA dataset according to the BMI and the staging of inflammatory activity and fibrosis, as assessed by METAVIR scoring^40^ on digital slides. Table S8 shows METAVIR scores, as well as anonymised IDs; BMI; *ACE2*, *COL1A1*, *COL3A1*, and *VCAN* mRNA levels; and aetiologies. METAVIR inflammation scores were dichotomised as *low* (0–1) and *high* (2–3). METAVIR fibrosis scores were dichotomised as *low* (0–1–2) and *high* (3–4). BMI was available for 30/41 patients, 60% of whom (18/30) had a BMI >23 (overweight). High mRNA expression of *ACE2*, *COL1A1*, *COL3A1*, and *VCAN* correlated with the histological grading of inflammation and fibrosis (Fig. 6A). Discriminant function analysis showed the percent of variance in *ACE2* mRNA levels explained by fibrosis (*METAVIR F*), inflammation (*METAVIR A*), and BMI in 41 patients (Fig. 6B). Inclusion of BMI increased the discriminatory ability of the model. The fact that *ACE2* mRNA levels correlated with the markers of hepatocyte metabolism *OTC* and *GLS2* in 30 patients with steatohepatitis confirms our previous findings of *ACE2* expression in metabolically competent hepatocytes.^29^ Taken together, these findings suggested that ACE2 expression was upregulated in chronic fibroinflammatory liver diseases in a context of patient overweight.

### Oleic and stearic acids induce steatosis in normal human hepatocytes and lead to increased *ACE2* mRNA expression *in vitro*

**Fig. 7:**
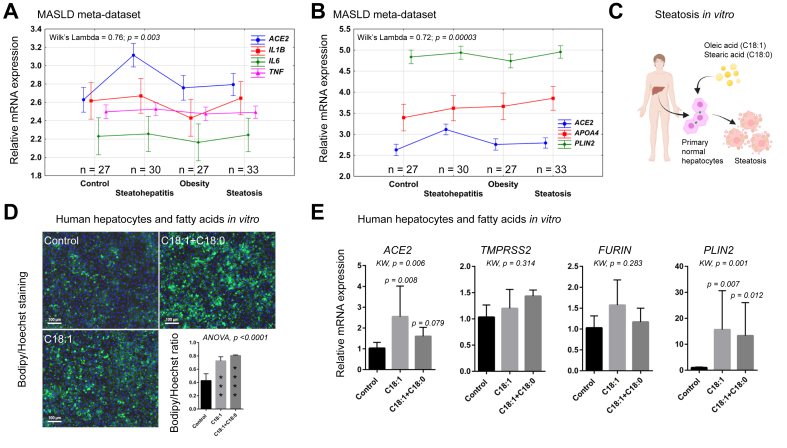
*ACE2* expression is induced by fatty acids in primary human hepatocytes *in vitro.* (A) Multiple discriminant analysis showing the proportion of variance in the MASLD meta-dataset explained by *ACE2* and the markers of inflammation *IL-1B, IL-6*, and *TNF*. The curve slopes show that *ACE2* accounts for the highest variance in steatohepatitis. Vertical bars indicate 95% CIs. (B) Multiple discriminant analysis showing the percent of variance in the MASLD meta-dataset explained by *ACE2* and the markers of steatosis *APOA4* and *PLIN2*. Vertical bars indicate 95% confidence intervals. (C) Schematic outline of *in vitro* induction of steatosis with fatty acids in primary human hepatocytes. Oleic acid alone is used to induce steatosis, whereas oleic and stearic acid together are used to model MASH-like metabolic effects.^41^ Cartoon constructed using BioRender (https://biorender.com/). (D) Representative images of primary human hepatocytes treated with 150 μM oleic acid (C18:1) or oleic plus stearic acids (C18:1 + C18:0; 150 μM each) for 1 week and labelled with the lipid stain Bodipy 493/503 (green) and the nuclear stain Hoechst (blue). The Bodipy/Hoechst ratio indicates fatty acid accumulation in hepatocytes. Asterisks indicate statistical differences with control (∗∗∗*p* <0.001, ∗∗∗∗*p* <0.0001). (E) Primary human hepatocytes from three patients were treated with 150 μM oleic acid (C18:1) or oleic plus stearic acids (C18:1 + C18:0; 150 μM each) for 1 week. Hepatocytes were seeded and treated in duplicate wells. RNA from each well was analysed in duplicate by real-time RT-PCR using the 2^–ΔΔCt^ method. Bars show means ± SD from three patients. Statistical intergroup differences were assessed using KW ANOVA as indicated, followed by Dunn’s *post hoc* multiple comparison test between control and fatty acid-treated wells. High *PLIN2*, a protein that coats lipid droplets, confirms the presence of steatosis. ACE2, angiotensin-converting enzyme 2; KW, Kruskal–Wallis; MASLD, metabolic dysfunction-associated steatotic liver disease; MASH, metabolic dysfunction-associated steatohepatitis; *PLIN2*, perilipin 2; TCGA, The Cancer Genome Atlas.

The above results led us to compare the impact of two statistical models of the variance of the MASLD meta-dataset by combining *ACE2* mRNA expression with either inflammatory cytokines or markers of fatty liver disease. Both models had similar impact on Wilk’s Lambda scores and confirmed the differences in *ACE2* expression between 27 control patients and 30 patients with steatohepatitis (Fig. 7A and B). These results are in line with a hypothetical interaction between fatty liver disease and chronic inflammation on ACE2 upregulation. To experimentally test this hypothesis, we challenged primary cultures of normal human hepatocytes from three patients by *in vitro* modelling the metabolic effects of steatohepatitis (Fig. 7C).^41^ Treatment of hepatocytes with the long-chain fatty acids oleic acid or oleic + stearic acids led to steatosis as shown by Bodipy staining (Fig. 7D) and increased expression of the steatosis marker perilipin 2 (*PLIN2*) mRNA (Fig. 7E). Under these conditions, *ACE2* expression was upregulated, unlike *TMPRSS2* and *FURIN.*

In summary, we conducted a multiparametric study of ACE2 expression and cellular sources in fatty liver diseases. We analysed liver bulk transcriptomes from eight independent sets totalling 404 patients with chronic fibroinflammatory liver diseases of different aetiologies. ACE2 protein was detected *in situ* at the sinusoidal and biliary poles of hepatocyte cords in nine patients with steatohepatitis and five controls. Single-cell liver RNA sequencing meta-analyses were done in 19 patients including those with NAFLD and alcohol-related liver disease. Finally, we *in vitro* modelled steatohepatitis in primary human hepatocyte cultures. In conclusion, converging evidence concurs to indicate that fibroinflammatory liver disease in a background of fatty liver leads to increased expression of the SARS-CoV-2 receptor ACE2.

## Discussion

We show that the expression of ACE2 is upregulated in chronic fibroinflammatory liver diseases in a context of patient overweight. In particular, the basal and apical poles of hepatocyte chords display an increased density of ACE2 receptors in patients with steatohepatitis. High liver ACE2 expression is related to the percentage of fat area, enhanced liver immune reactivity, and fibrogenesis. *In vitro* modelling of steatohepatitis by treating primary normal human hepatocytes with the long-chain fatty acids oleic and stearic acids leads to increased *ACE2* mRNA expression, confirming the molecular data obtained from clinical samples.

ACE2 was also detected in CLEC4M^+^ sinusoidal endothelial cells and in CD34^+^ portal capillaries in steatohepatitis. CLEC4M may facilitate the transfer of viruses from the sinusoidal endothelium to ACE2-positive cells.^19^ Although TMPRSS2 protein was not detected in normal liver, it was co-detected with ACE2 in hepatocytes in steatohepatitis. Therefore, ACE2, TMPRSS2, and CLEC4M could support viral infection through the space of Disse. In consistency with these findings, SARS-CoV-2 has been detected within endothelial cells in portal veins^42^ and sinusoidal microthrombi^43^ and associated with vascular dysfunction, such as occlusive thrombosis, in patients who died from COVID-19.^11^^,^^44^

In contrast with our findings, a recent report^45^ concluded that there was no evidence for an increased *ACE2* expression in MASLD. The authors found no difference in *ACE2* mRNA expression between MASLD, steatosis, and MASH after analysis of the GSE48452^26^ transcriptomic microarray dataset. First, the GSE48452 dataset (n = 54) is included within our meta-dataset consisting of 243 patients with fatty liver disease. Second, we agree that, in this dataset, there is no difference between lean patients without MASLD and the other groups. However, *ACE2* mRNA levels were higher in patients with MASH than in obese patients without MASLD and in patients with simple steatosis (Wilcoxon test adjusted for multiple testing using Benjamini and Hochberg’s correction; Fig. S20). In consistency with our results, *ACE2* and *TMPRSS2* upregulation in MASH correlates with activity score.^32^ Moreover, international studies including over 60 million patients revealed a higher risk for COVID-19 in metabolic syndrome. In particular, patients with MASH,^5^ MASLD,^46^ and NAFLD^4^ are at high risk of severe COVID-19 and liver injury.

Our data suggested that a background of fatty liver disease is important for upregulation of ACE2 to occur. Several lines of evidence substantiate this hypothesis. First, patients with alcoholic, HBV, or HCV hepatitis did not present increased *ACE2* mRNA levels. By contrast, in overweight patients, *ACE2* mRNA was correlated with fibroinflammatory activity. Discriminant function analysis showed that the variance in *ACE2* mRNA levels was better explained by a model including BMI than not.

Second, our model of the metabolic effects of steatohepatitis in primary normal human hepatocytes using long-chain fatty acids revealed that steatosis upregulates *ACE2* expression *in vitro*. In consistency with our results, primary human hepatocytes where steatosis had been induced by methionine and choline deprivation are more vulnerable to SARS-CoV-2 infection.^15^ However, induction of steatosis in HepG2 hepatoblastoma cells with palmitic acid (PA) has led to contrasting results. PA alone was reported to increase^47^ and reduce^48^ ACE2 expression. In the latter report, it was the association of PA with the glucagon-like peptide liraglutide that activated the Ang_1–7_/Mas axis downstream ACE2, reducing inflammation in fatty liver disease in mice. Similarly, a murine model of Ang II-dependent hypertension supplemented with omega-3 fatty acids increased *ACE2* and reduced inflammation.^49^

Third, treatment of primary human hepatocytes with recombinant IL-6, IL-1B, TNFA, or LPS decreased *ACE2* mRNA expression. Similar results were reported by other authors.^38^ Indeed, inflammatory cytokines lead to a loss of hepatocyte identity.^39^ Hepatocyte^29^ or cholangiocyte^12^ dedifferentiation results in loss of ACE2 expression. These findings agree with our observation that *ACE2* mRNA levels correlate with the markers of hepatocyte differentiation *OTC* and *GLS2.*

An alternative mechanism leading to ACE2 upregulation could result from the antiviral IFN response. Type 1 IFNs, specifically IFNA2, whose levels are increased in patients with severe COVID-19, increase ACE2 expression and viral copy number *in vitro*.^38^ The same mechanism could explain higher ACE2 expression in primary hepatocytes infected with HCV *in vitro*.^30^ However, our meta-analysis of 30 HCV-infected patients from GSE48445 (Fig. S17) revealed that treatment with pegylated IFNA2 resulted in decreased *ACE2* expression. This raises questions about the interactions between HCV viral load and the expression of endogenous IFNA2 and ACE2 across the timeline of antiviral treatment in the era of direct antiviral agents against HCV.

Fourth, by digital sectioning of bile canaliculi using confocal microscopy, we found that the lumen of bile canaliculi in steatohepatitis contained ACE2. Indeed, ACE2 is shed from the cell surface by ADAM17^50^ and is thus found abundantly in the human bile proteome.^35^ Moreover, high serum levels of ACE2 are found in cholestasis because bile acids control *ACE2* gene expression through farnesoid X receptor (FXR) response elements in its promoter.^12^ Fatty acids can also activate FXR, and inflammation and hepatocyte ballooning in steatohepatitis can induce cholestasis. Therefore, FXR and ACE2 could be involved in a regulatory loop in fatty liver disease. In support of this hypothesis, FXR downregulates liver lipogenesis, thus protecting against NAFLD,^51^ and ACE2 contributes to postprandial amino acid absorption,^13^ thus improving hepatocyte homoeostasis.

Altogether, this body of evidence raises the hypothesis that high ACE2 expression in response to an excess in fatty acids could protect hepatocytes from inflammation and activate fatty acid oxidation through the Ang_1–7_/Mas axis. In turn, SARS-CoV-2 evolution may have exploited this regulatory loop, which increases susceptibility to infection. The concomitance of enhanced innate immune reactivity with increased availability of ACE2 receptors in a backdrop of fatty liver disease may promote viral infection, endocytosis of virus-receptor complexes, rapid cell surface ACE2 depletion, and finally metabolic decompensation and amplified inflammation.

## Financial support

This work was supported by 10.13039/501100001677Inserm; Univ Rennes 1; Ministère de l’Enseignement Supérieur; 10.13039/501100006364Institut National Du Cancer, grant no. INCA_12688; and Ligue Nationale Contre le Cancer 2018, Comités d’Ille-et-Vilaine et Vendée.

## Authors’ contributions

Study design: OM, LD, LC, AC, BC, JM, CA. Cell culture experiments and molecular biology analyses: GA, ELF, CA, JM. Meta-datasets merging and pretreatment: LD, RD. Database search: LD, LC, CL, OM. Anatomic pathology analysis: BT, LC, OM. Immunohistochemistry: GG. Statistics: LD, LC, OM. Image analysis: LC, OM. Data analysis: LD, LC, OM. Manuscript preparation: LD, LC, OM. Manuscript editing: all authors.

## Data availability statement

Data associated with this study are available in nine Supplementary tables. Transcriptomics datasets and digital histological slides are publicly available from the Gene Expression Omnibus (GEO-NCBI), TCGA, and the Digital Slide Archive website. The MASLD meta-dataset results from the pretreatment, merging, and batch effect correction of raw transcriptomic data from publicly available datasets from different technical platforms. Meta-dataset construction has been previously described.^28^ The merged user-friendly dataset can be made available from the authors upon request.

## Conflicts of interest

The authors of this study declare that they do not have any conflict of interest.

Please refer to the accompanying ICMJE disclosure forms for further details.
