# Supplementary material for: SARS-CoV-2 receptor ACE2 is upregulated by fatty acids in human MASH
Source: JHEP Rep. 2023 Oct 13;6(1):100936. doi: 10.1016/j.jhepr.2023.100936 (PMC10698276; doi:10.1016/j.jhepr.2023.100936)
Supplement: Multimedia component 1 [file mmc1.pdf]

SARS-CoV-2 receptor ACE2 is upregulated by fatty acids in human MASH

Luis Cano, Lise Desquilles, Gevorg Ghukasyan, Gaëlle Angenard, Clémence Landreau, Anne Corlu, Bruno Clément, Bruno Turlin, Eric Le Ferrec, Caroline Aninat, Julie Massart, Orlando Musso

Table of contents

Supplementary Methods………………………………………………………………….. 2

Supplementary Figure Legends………………………………………………………… 7

Supplementary Table Legends…………………………………………………………16

Supplementary References……………………………………………………………...19

Supplementary Figures…………………………………………………………………..23

Supplementary Tables…………………………………………………………………...46

Supplementary Methods

Human primary cells: Gene expression was explored in the *Primary Cell Atlas*,[1] a transcriptomic microarray meta-dataset that assembles 745 samples (GSE49910). Expression data were quantile-normalized and top ten differentially expressed samples across tissues were plotted.

Immunohistochemistry

Routine formalin-fixed, paraffin-embedded tissue blocks from the Anatomic Pathology laboratory, Rennes University Hospital, were used to prepare 5 µm sections mounted on Super Frost Plus slides (VWR International). Antibodies used, incubation conditions, horseradish peroxidase and tyramide-based signal amplification are summarized in Table S2. Digital images were obtained with either a single focus (Nanozoomer, Hamamatsu Photonics) or confocal (3D-Histech) slide scanner. Image analysis quantitation was performed with the HALO Spatial Analysis software (Indica Labs). Immunohistochemistry, digital imaging and data acquisition were performed in an ISO-9001-certified core facility (High Precision Histopathology, H2P2, CNRS 3840, INSERM 018, Rennes 1 University). Data analysis was performed with R 3.6.2 and the R package dplyr 0.8.5 for data cleaning and transformation. The Mann-Whitney U test was used to assess the differences between means.

Cell culture and treatments

The Human Macrovascular Endothelial cell line (HMEC-1) was obtained and cultured as previously described.[2] Briefly, cells were routinely cultured in MCDB-131 medium

with hydrocortisone (1 μg/ml), epidermal growth factor (10 ng/ml), penicillin (50 units/ml), streptomycin (50 units/ml), L-glutamine (10 mM) and with 10% foetal bovine serum. Cells were treated with the indicated cytokines or LPS at 90% confluence after overnight culture in serum-free medium.

Primary human hepatocyte isolation and culture

Primary human adult hepatocytes from non-tumor histologically normal liver fragments were obtained from Biopredic International (Saint-Grégoire, France). Donors were patients undergoing resection of primary or liver secondary tumors. Prior to liver tissue perfusion and dissociation for hepatocyte isolation, the hepatectomy specimen was examined by an anatomic pathologist. Written informed consent was obtained from the patients. The procedure, approved by INSERM's Review Board and the local Ethics Committee, was performed as described.[3]

Primary human hepatocyte treatments

Treatment of primary human hepatocytes with cytokines and LPS:

Hepatocytes were seeded at a density of 250,000 cells/cm^2 in 24-well plates and fed with Williams' E medium supplemented with 10 % foetal calf serum (FCS), 100 units/ml penicillin, 100 μg/ml streptomycin, 5 μg/ml insulin, 2 mM glutamine, and 50 μM hydrocortisone hemisuccinate. One day after seeding, the medium was complemented with 2% DMSO and renewed daily. Human hepatocytes were used three to five days after seeding. Treatments with cytokines (IL-6, 20 ng/ml; IL-1B, 10 ng/ml; TNFA, 100 ng/ml) or LPS (10 ng/ml) were performed in FCS- and DMSO-deprived medium. Cells

were harvested after 12 hr of treatment. Human recombinant IL-6, IL1B and TNFA were purchased from Biotechne (R&D Systems) and LPS *(E. coli* 055:B5) *was* from Sigma.

Treatment of primary human hepatocytes with fatty acids:

Cells were seeded in collagen-coated 24-well plates at a density of $0.4.10^6$ cells/well and cultured at 37°C with 5% CO2 and saturating humidity in William’s E medium supplemented with 5% FCS, 100 U/ml penicillin, 100 µg/ml streptomycin, 2 mM glutamine, 5 µg/ml insulin and 1 µM hydrocortisone hemisuccinate. After cell adherence to the culture plates (24 hr), hepatocytes were treated every other day for 7 days with 150 µM oleic acid (C18:1) or 150 µM oleic acid + 150 µM stearic acid (C18:1+C18:0). Fatty acid stock solutions (5 mM) were prepared in heated NaOH 0.1 M and then diluted in prewarmed 10% fatty acid-free BSA solution in serum-free William’s E medium. Control cell medium was supplemented with the same amount of NaOH/FFA-free BSA mixture.

Neutral lipid staining

Cells were incubated for 15 min at 37°C with 2 µM BODIPY 493/503 (Molecular Probes, ThermoFisher Scientific), fixed with formalin (Sigma-Aldrich) for 1 hr and stained with 1 µg/ml Hoechst 33342 (ThermoFisher Scientific) for 20 min.

RNA extraction and Real-time PCR

For primary human hepatocytes seeded in plastic (see cytokine and LPS treatments above), total RNA was extracted with the Nucleospin RNA isolation kit from Macherey-Nagel (Düren, Germany), with DNAse treatment, followed by analysis of RNA concentration and 260/280 and 230/260 ratios with a Nanodrop 1000 (Thermo Fisher Scientific, Waltham, MA). RNA was reverse-transcribed using the High-Capacity cDNA Reverse Transcription kit (Applied Biosystems, Woolston, UK). Gene expression was measured by real-time PCR using SYBR Green PCR Master Mix (Applied Biosystems) and a 384-well-QuantStudio 7 Flex Real-Time PCR System (Thermo Fisher). Primer sequences are shown in Table S3. *HPRT1* or *TBP* were used as housekeeping genes and results analyzed by the $2^{-\Delta\Delta Ct}$ method using control conditions as calibrators.

For primary human hepatocytes seeded in collagen (see fatty acid treatments above), total RNA was isolated with Trizol according to the manufacturer's recommendations (Life Technologies). Total RNA concentration was quantified spectrophotometrically (NanoDrop ND-1000 Spectrophotometer, ThermoFisher Scientific, Waltham, MA). RNA was reverse-transcribed to cDNA using the High Capacity cDNA RT kit. Gene expression was determined by real-time PCR using SYBR Green (Life Technologies), applying the the $2^{-\Delta\Delta Ct}$ method and using *HPRT1* gene expression as an internal standard. Primer sequences are shown in Table S3.

Deconvolution of immune cell populations from transcriptomic data in fatty liver disease

Deconvolution of immune cell populations to construct phenetic diagrams (i.e., immune phenotypic scoring or immunophenoscoring) was done by applying previously

described algorithms.[4] Data were processed using the R script provided by the authors (https://github.com/icbi-lab/Immunophenogram). An alternative approach to infer the importance of leucocyte cell populations infiltrating fatty liver disease samples was to search for expression of immune cell subset marker RNA using the Immune Response in Silico (IRiS)[5] microarray dataset.

Statistical analysis

Correlations between genes were calculated using either the Pearson's or Spearman's R methods. Continuous variables were compared by parametric testing (Student's *t*-test and ANOVA) or non-parametric testing (Mann-Whitney U test); whereas categorical data were compared with Fisher's exact test. *P* values were adjusted for multiple testing using the Benjamini-Hochberg procedure.

Weighted Gene Correlation Network Analysis (WGCNA) was performed using the *WGCNA[6]* R package, as we previously described.[7] The network file was then exported to Cytoscape.[8] Functional analysis and GO enrichment were conducted after gene module detection and results were summarized using Express Analysis from Metascape[9] or REVIGO,[10] as indicated.

Supplementary Figure Legends

Fig. S1. Positive controls for ACE2 (A, B) and TMPRSS2 (C, D) antibodies. Immunoperoxidase staining *(brown)* and light hematoxylin counterstaining *(blue),* in kidney (A), intestine (B) and prostate (C, D). In the kidney, ACE2 positive signal is seen in the parietal cell layer of the Bowman's capsule and the convoluted tubules (A). ACE2 is detected in the apical pole of the intestinal epithelium (B). TMPRSS2 is detected in the apical pole of the glandular epithelium and in surrounding smooth muscle (C, D). Digital slides were acquired in a slide scanner with a X20 objective (Nanozoomer, Hamamatsu Photonics).

Fig. S2. (A) *ACE2* and *TMPRSS2* mRNA expression data extracted from the Primary Cell Atlas meta-database,[1] which assembles 745 primary cell samples from over 100 studies, exploited as described.[11, 12] Both *ACE2* and *TMPRSS2* mRNAs are detected in primary human hepatocytes. (B, C) ACE2 expression in normal human liver. (B) In a portal tract, ACE2 is detected in bile ducts at low magnification *(arrows).* (C) Higher power view of the region of interest framed in *red* in *B.* ACE2 signal in a bile duct *(red arrowhead),* in vessels within the portal tract and along the limiting plate *(green arrowheads)*, as well as in cells lining the sinusoids *(black arrowheads).* (D-G) Co-expression of ACE2 and TMPRSS2 in hepatocytes. ACE2 *(red, TRITC),* TMPRSS2 *(orange, Cy5)* and the hepatocyte marker HepPar-1 *(green, FITC)* were detected with a microscope scanner and a X40 objective. Images are Z-stacks of four 500 nm focusing steps.

Fig. S3. ACE2 is not detected in CD45 positive leukocytes. Co- staining of ACE2 *(red, TRITC)* and CD45 *(green, FITC)* in the liver parenchyma. (A-C) At high magnification, signal in *yellow* corresponds to overlapping endothelial and leukocyte cell membranes. Immunofluorescence digital images were acquired with a microscope scanner using a X40 objective (Nanozoomer, Hamamatsu Photonics). Images are Z-stacks of four 500 nm focusing steps.

Fig. S4A-D. ACE2 and CD68 in liver sinusoids. Co-staining of ACE2 *(red, TRITC)* and CD68 *(green, FITC)* in the liver parenchyma. (A-D) Overlaps of both epitopes are seen in *yellow* in some cells (shown in *D).* Immunofluorescence digital images were acquired with a confocal microscope scanner and a X40 objective. Images are Z-stacks of four 500 nm focusing steps.

Fig. S4E-G. ACE2 and CD68 in liver sinusoids. Co-staining of ACE2 *(red, TRITC)* and CD68 *(green, FITC)* in the liver parenchyma. (A-D) Overlaps of both epitopes are seen in *yellow* in some cells (shown in *G).* Image deconvolution across 500 nm steps in the Z axis shows that CD68 (+) Kupffer cell membrane extensions line ACE2 (+) sinusoidal endothelial cells. Immunofluorescence digital images were acquired with a confocal microscope scanner and a X40 objective. Images are Z-stacks of four 500 nm focusing steps.

Fig. S5. ACE2 is not detected in CD3 positive lymphocytes or in ACTA2-(a.k.a alpha smooth muscle actin)-positive myofibroblasts. Co-staining of

ACE2 *(red, TRITC)* and CD3 *(green, FITC)* in the liver parenchyma (A-B) or ACE2 and ACTA2 *(green, FITC)* at the interface between the liver parenchyma and a fibrous septum (C). Immunofluorescence digital images were acquired with a microscope scanner using a X40 objective (Nanozoomer, Hamamatsu Photonics, in *A)* or with a confocal microscope scanner and a X40 objective. Images are Z-stacks of four 500 nm focusing steps.

Fig. S6. Single cell RNA sequencing data from histologically normal liver samples obtained from nine patients undergoing resection of colorectal cancer metastases or cholangiocarcinoma, without underlying liver disease. Data were obtained from Aizarani et al.,[13] through a dedicated web interface (http://human-liver-cell-atlas.ie-freiburg.mpg.de/). Arrows show *ACE2* mRNA expression in comparison with *TMPRSS2, FURIN, DPP4* and albumin *(ALB)* mRNAs; the sinusoidal endothelial cell *(SEC)* marker *CLEC4M;* the periportal *(PP)* markers *PCK1, ALDOB, HAL;* the perivenous *(PV)* marker *GLUL;* the cholangiocyte *(Cho)* and liver progenitor cell markers *EPCAM, KRT19, SOX9;* the capillary endothelial cell *(Endo)* marker *CD34;* the bile canaliculi *(Canaliculi)* marker *ABCC2*; the myofibroblast *(Myof)* marker *ACTA2*; the liver basement membrane *(BM)* markers *COL4A1 and LAMC1;* the monocyte *(Mo)* marker *CD163;* the Kupffer cell marker *ITGAM;* the B lymphocyte marker *CD37;* the T lymphocyte marker *CD3D;* the NK cell markers *CCL4* and *KLRF1* and the NKT cell marker *CCL5.* Log_2 expression intensities are indicated by a heat scale. Overall, *ACE2* mRNA levels are low in all cell populations tested, but detected in scattered cells in the clusters containing sinusoidal endothelial cells; periportal and perivenous hepatocytes and cholangiocytes.

Fig. S7. Single cell RNA sequencing data from five normal *(uninjured)* and five cirrhotic human livers (2x NAFLD, 2x alcohol-related liver disease, 1x primary biliary cirrhosis), extracted from the Ramachandran *et al.* dataset,[14] through a dedicated web interface (https://shiny.igmm.ed.ac.uk/livercellatlas/), where violin plots can be obtained from integrated Seurat functions (ggplot2, pheatmap and grid R packages).[14] (A) *ACE2,* the sinunoidal endothelial cell marker *CLECL4M, albumin (ALB)*, the hepatocyte marker *Cytokeratin 18 (KRT18)* and the myofibroblast marker *alpha smooth muscle actin (ACTA2)* in MP, mononuclear phagocytes; pDC, plasmocytoid dendritic cells; ILC, innate lymphoid cells; Tc, T lymphocytes; Bc, B lymphocytes; Plc, plasma cells; MC, mast cells; Endo, endothelial cells; Mes, mesenchymal cells; Meso, mesothelial cells; Hc, hepatocytes; Cho, cholangiocytes.
(B) *ACE2,* the capillary endothelial cell marker *CD34* and the liver sinusoidal endothelial cell marker *CLEC4M* in the indicated cell populations. (C) *TMPRSS2, FURIN* and *DPP4* in the indicated cell populations.

Fig. S8. (A) Merging of three transcriptomic microarray datasets into a fatty liver disease meta-dataset of 243 human liver samples: GSE33814,[15] consists of 12 normal livers, 19 steatoses, and 12 steatohepatitides; GSE48452[16] consists of 14 normal livers, 27 livers from obese patients, 14 steatoses and 18 non-alcoholic steatohepatitides (MASH); GSE83452[17] consists of 231 samples from which 126 MASHs were extracted for study. Altogether, the meta-dataset includes 27 normal livers; 27 livers from obese patients; 33 steatoses; 12 steatohepatitides and 144 MASHs. Batch effect was corrected using the ComBat algorithm (*sva* R package). Raw expression data were quantile-normalized and log2-transformed. (B) Expression of the

SARS-CoV-2 co-receptor TMPRSS2, the MERS-CoV receptor DPP4 and the sinusoidal endothelial cell marker and SARS-CoV-2 co-receptor *CLEC4M* in the fatty liver disease meta-dataset. Statistical significance of the difference between groups was calculated with analysis of variance (ANOVA) followed by Tuckey post-hoc test *(*, p<0.05; **, p<0.01; ***, p<0.001)*.

Fig. S9. RNA expression of immune cell subset-specific markers in circulating immune cells isolated with cell-surface-specific antibodies and analyzed by cDNA microarrays. Raw data were extracted from the GSE22886 dataset.[5] Only values above the mean expression level for each probe were analyzed.

Fig. S10. ***ACE2, TMPRSS2* and *DPP4* mRNAs are detected at very low levels in circulating immune cells.** (A) Expression of *ACE2, TMPRSS2* and *DPP4* mRNAs in specifically isolated circulating immune cell subsets from the GSE22886[5] dataset. The number of observations in each cell subset is shown in Table S9. (B) The expression of *ACE2, TMPRSS2* and *DPP4* mRNAs in fatty disease liver tissues correlates with immune cell markers in 13 normal livers,

19 steatoses and 12 steatohepatites in the transcriptomic microarray dataset GSE33814. Immune cell populations in liver tissues were deconvoluted as shown in Fig. S10, according to the GSE22886 dataset.[5] The color scale for the heatmap of Pearson’s correlation coefficients and the color codes for the immune cell populations are shown on the right.

Fig. S11. (A) *ACE2, TMPRSS2* and *DPP4* mRNA levels correlate with markers of specific immune cell subsets in 14 normal livers, 27 livers from obese patients, 14 steatoses and 18 MASHs in the GSE48452[16] transcriptomic dataset. The color scale for the heatmap of Pearson's correlation coefficients and the color codes for immune cell populations are shown on the right. (B) MASH samples from the GSE48452[16] dataset show increased expression of molecular markers for T cells. ANOVA was followed by Tuckey post-hoc test to assess the statistical significance of the difference between *control* and *obesity* or *steatosis* or *MASH (*, $p<0.05$).*

Fig. S12. (A) Immunophenogram classifying immune reactivity parameters in four immunogenicity functional families: MHC, Major Histocompatibility Complex; EC, Effector Cells; SC, Suppressor Cells; CP, Check Points. Enhancement/suppression of immune reactivity are indicated by (+)/(–). Parameters' full names and gene symbols are indicated in Table S5. (B-D) Immunophenograms for each liver sample from the GSE33814 dataset. (E) Parameters' Z-scores and functional families are shown as red/blue heat map and grayscale, respectively.

Fig. S13. Relatedness dendrogram (A) and correlation heatmap (B) of modules identified by Weighted Gene Coexpression Network Analysis (WGCNA) in the fatty liver disease metadataset of 243 human liver samples, which includes 27 normal livers; 27 livers from obese patients; 33 steatoses; 12 steatohepatites and 144 MASHs. After network analysis with the *WGCNA* R package,[6] the dendrogram shows the eigengene relatedness between modules. Modules are sub-networks of highly

correlated genes. The eigengene represents the first principal component of each module transcriptome across samples. The heatmap shows the pairwise correlation coefficients of module eigengenes, with *red* representing highly positive; *blue* highly negative and *white* no pairwise correlations. The *blue box* indicates three highly correlated modules that are enriched in steatohepatitis *(see Fig. S14).* Color coding of module names is arbitrary and unrelated to phenotype.

Fig. S14. Matrix of module-trait relationship after analysis of 27 normal livers; 27 livers from obese patients; 33 steatoses; 12 steatohepatites and 144 MASHs by WGCNA. The purpose of this matrix is to identify the gene expression modules that are the most highly associated with at least one of the patient groups. Correlation coefficients and statistical significance are indicated within each cell of the matrix. They are color-coded according to the heatmap on the right, where *red* represents highly positive correlation; *blue* highly negative correlation and *white* no correlation. The *blue frame* highlights the three modules the most highly associated with steatohepatitis. The number of patients in each group is indicated below. Color coding of module names is arbitrary and unrelated to phenotype.

Fig. S15. Representation of the three most highly correlated gene network modules *(grey; red; sky blue).* The hub gene is colored in *pink* at the center of the module. It corresponds to the node with the highest number of connections *(black lines)*. Node size is proportional to the number of connections*.* The network files for each module (Table S7) were exported to Cytoscape[8] for graphical representation.

Fig. S16. Functional analysis and gene ontology enrichment of the three gene network modules *(grey; red; sky blue)* the most highly correlated with steatohepatitis. Results were summarized using *Express Analysis* from Metascape.[9]

Fig. S17. (A) Expression of the indicated genes in alcoholic hepatitis *versus* control livers in the GSE28619 transcriptomic dataset.[18] *TIMP1* and *CST3* are shown as markers of fibrosis and inflammation, respectively. (B) Expression of *ACE2* mRNA in peripheral blood mononuclear cells in response to acute HCV infection, according to the GSE119117 RNA sequencing dataset shows no significant variation in *ACE2* mRNA levels.[19] (C) Pegylated interferon alpha 2B –but not alpha 2A, decreases *ACE2* mRNA expression in 30 HCV patients, according to the GSE48445 microarray dataset.[20] (D) Pegylated interferon alpha 2A plus adefovir do not change *ACE2* mRNA levels in 15 HBV patients regardless of the HBeAg and responder/non responder status, according to the GSE54747 microarray dataset.[21]

Fig. S18. Primary hepatocytes from three patients were treated with IL6 (20 ng/ml), IL1B and LPS (10 ng/ml) and TNFA (100 ng/ml) without serum during 12 hr. Hepatocytes were seeded and treated in triplicate wells. RNA from each well was analyzed in duplicate by real time RT-PCR using the $2^{-\Delta\Delta Ct}$ method. Bars indicate means ± SD. Statistical intergroup differences were assessed by Kruskal-Wallis *(KW)* ANOVA as indicated, followed by the post-hoc Dunn's multiple comparison test comparing control with cytokine/LPS treatment (*, p<0.05; **, p<0.01; ***, p<0.001).

Fig. S19. Human microvascular endothelial cells (HMEC-1 cell line) were treated with IL6 (20 ng/ml), IL1B, LPS (10 ng/ml) or TNFA (100 ng/ml) without serum during 12 hr. Cells were seeded and treated in duplicate wells. RNA from each well was analyzed in duplicate by real time RT-PCR using the $2^{-\Delta\Delta Ct}$ method. Bars indicate means ± SD. *HMEC_1* and *HMEC_2* denote two separate experiments. *CXCL8* and *CCL2* mRNA expression are shown as positive controls of chemokine induction in response to the applied treatment.

Fig. S20. *ACE2* mRNA expression in the GSE48452 dataset consisting of transcriptomic microarray data from 12 controls (lean patients without MASLD), 16 obese patients without MASLD (healthy obese), 17 MASH and 9 patients with steatosis. Box-and-whisker plots represent median plus 1st and 3rd quartiles. Vertical bars represent [1.5 x interquartile range, above the 3rd quartile] and [1.5 x interquartile, range below the 1st quartile]. Outliers (present only in MASH and steatosis as colored dots) indicate values > 1.5 folds and < 3 folds the interquartile range above and below the boxes, respectively. The GSE48452 was explored using RMA-normalized expression data (*oligo* R package), as described.[22] Samples obtained after bariatric surgery were excluded from the analysis. All pairwise comparisons were performed using the non-parametric Wilcoxon's test (*stats* R package). *P* values were adjusted for multiple testing using the Benjamini-Hochberg's procedure.

Supplementary Table Legends

Table S1. Patient clinical and biological data; steatosis, ballooning, inflammation, NAFLD Activity Score (NAS) and MASH grading. NAS assesses active injury defined by the sum of the scores for steatosis (0-3), lobular inflammation (0-3) and ballooning (0-2); thus ranging from 0 to 8.[23] MASH grading assesses the necro- inflammatory activity for steatohepatitis as mild (grade 1); moderate (grade 2) and severe (grade 3), as described.[24] The control case n° U1241_10 could have been diagnosed as MASLD on the basis of the hepatic steatosis index[25], plus a BMI >25 kg/m^2 and type II diabetes. However, histological assessment of the partial hepatectomy specimen revealed <5% steatosis, mild portal fibrosis and no inflammation. Thus, liver histology for this case was not compatible with MASLD, NAFLD or MASH diagnostic criteria.[26, 27]

Table S2. Antibodies and immunohistochemical methods.

Table S3. Oligonucleotides used for real-time PCR.

Table S4. Numbers of available observations for the listed genes after meta-dataset pretreatment. The meta-dataset was constructed after merging the following datasets: GSE33814; GSE48452; GSE83452. Quantile normalization and batch effect correction were applied, as shown in Supplementary Figure 8A.

Table S5. Full names and gene symbols for immunogenicity functional families shown in Fig. 5A and Fig. S12. Positive and negative modulators are highlighted by red/green cell coloring, respectively.

Table S6. Statistical significance of the differences between mean expression levels of immunophenoscore parameters shown in Fig. 5A and Fig. S12. Positive and negative modulators are highlighted by red/green cell coloring, respectively.

Table S7. Excel file containing three spreadsheets named after the three color-coded names of the modules associated with steatohepatitis, as shown in Supplementary Figs. 13-16. Module names are: *Grey60*; *Red* and *Skyblue*. Spreadsheet columns headings: *"names",* gene symbols. *"Nb_edges",* measures the number of genes with which a given gene is correlated. *"Gene_Significance",* measures the biological significance of a given gene in a 0 to 1 range; the higher the absolute value, the more biologically significant a given gene is. Gene significance can take on positive or negative values. *"Module_Membership",* measures the correlation of a given gene with the first principal component of the module in a 0 to 1 absolute range. Module membership can take on positive or negative values. Highly connected hub genes will have high module membership values. Measures were obtained by Weighted Gene Correlation Network Analysis (WGCNA) in the fatty liver disease metadataset of 243 human liver samples, which includes 27 normal livers; 27 livers from obese patients; 33 steatoses; 12 steatohepatites and 144 MASHs.

Table S8. METAVIR[28] liver fibrosis and inflammation scores in 41 non-tumor livers from the TCGA-LIHC dataset. Digital hematoxylin-eosin-stained slides were accessed from the publicly available platform Cancer Digital Archive website (https://cancer.digitalslidearchive.org/). Histological analysis was performed by two observers trained in liver pathology (OM & LC), blinded to molecular profiling. Anonymized IDs are provided in the 1st column. Assessed features are indicated as column headings. Normalized mRNA expression values for *ACE2, COL1A1, COL3A1* and *VCAN* are indicated.

Table S9. Number of observations in each cell subset from the GSE22886 IRiS Immune Response in Silico dataset.

Supplementary References

[1] Mabbott NA, Baillie JK, Brown H, Freeman TC, Hume DA. An expression atlas of human primary cells: inference of gene function from coexpression networks. BMC Genomics 2013;14:632.

[2] Le Goff M, Lagadic-Gossmann D, Latour R, Podechard N, Grova N, Gauffre F, et al. PAHs increase the production of extracellular vesicles both in vitro in endothelial cells and in vivo in urines from rats. Environ Pollut 2019;255:113171.

[3] Guguen-Guillouzo C, Campion JP, Brissot P, Glaise D, Launois B, Bourel M, et al. High yield preparation of isolated human adult hepatocytes by enzymatic perfusion of the liver. Cell Biol Int Rep 1982;6:625-628.

[4] Charoentong P, Finotello F, Angelova M, Mayer C, Efremova M, Rieder D, et al. Pan-cancer Immunogenomic Analyses Reveal Genotype-Immunophenotype Relationships and Predictors of Response to Checkpoint Blockade. Cell Rep 2017;18:248-262.

[5] Abbas AR, Baldwin D, Ma Y, Ouyang W, Gurney A, Martin F, et al. Immune response in silico (IRIS): immune-specific genes identified from a compendium of microarray expression data. Genes & Immunity 2005;6:319-331.

[6] Langfelder P, Horvath S. WGCNA: an R package for weighted correlation network analysis. BMC Bioinformatics 2008;9:559.

[7] Desquilles L, Musso O. Metabolic Networks: Weighted Gene Correlation Network Analysis. Methods Mol Biol 2023;2675:317-325.

[8] Shannon P, Markiel A, Ozier O, Baliga NS, Wang JT, Ramage D, et al. Cytoscape: a software environment for integrated models of biomolecular interaction networks. Genome Res 2003;13:2498-2504.

[9] Zhou Y, Zhou B, Pache L, Chang M, Khodabakhshi AH, Tanaseichuk O, et al. Metascape provides a biologist-oriented resource for the analysis of systems-level datasets. Nat Commun 2019;10:1523.

[10] Supek F, Bosnjak M, Skunca N, Smuc T. REVIGO summarizes and visualizes long lists of gene ontology terms. PloS one 2011;6:e21800.

[11] Desert R, Mebarki S, Desille M, Sicard M, Lavergne E, Renaud S, et al. "Fibrous nests" in human hepatocellular carcinoma express a Wnt-induced gene signature associated with poor clinical outcome. Int J Biochem Cell Biol 2016;81(Pt A):195-207.

[12] Mebarki S, Desert R, Sulpice L, Sicard M, Desille M, Canal F, et al. De novo HAPLN1 expression hallmarks Wnt-induced stem cell and fibrogenic networks leading to aggressive human hepatocellular carcinomas. Oncotarget 2016;7:39026-39043.

[13] Aizarani N, Saviano A, Sagar, Mailly L, Durand S, Herman JS, et al. A human liver cell atlas reveals heterogeneity and epithelial progenitors. Nature 2019;572:199-204.

[14] Ramachandran P, Dobie R, Wilson-Kanamori JR, Dora EF, Henderson BEP, Luu NT, et al. Resolving the fibrotic niche of human liver cirrhosis at single-cell level. Nature 2019;575:512-518.

[15] Starmann J, Falth M, Spindelbock W, Lanz KL, Lackner C, Zatloukal K, et al. Gene expression profiling unravels cancer-related hepatic molecular signatures in steatohepatitis but not in steatosis. PloS one 2012;7:e46584.

[16] Ahrens M, Ammerpohl O, von Schönfels W, Kolarova J, Bens S, Itzel T, et al. DNA methylation analysis in nonalcoholic fatty liver disease suggests distinct disease-specific and remodeling signatures after bariatric surgery. Cell Metab 2013;18:296-302.

[17] Lefebvre P, Lalloyer F, Baugé E, Pawlak M, Gheeraert C, Dehondt H, et al. Interspecies NASH disease activity whole-genome profiling identifies a fibrogenic role of PPARα-regulated dermatopontin. JCI Insight 2017;2:6.

[18] Affo S, Dominguez M, Lozano JJ, Sancho-Bru P, Rodrigo-Torres D, Morales-Ibanez O, et al. Transcriptome analysis identifies TNF superfamily receptors as potential therapeutic targets in alcoholic hepatitis. Gut 2013;62:452-460.

[19] Rosenberg BR, Depla M, Freije CA, Gaucher D, Mazouz S, Boisvert M, et al. Longitudinal transcriptomic characterization of the immune response to acute hepatitis C virus infection in patients with spontaneous viral clearance. PLoS Pathog 2018;14:e1007290.

[20] Dill MT, Makowska Z, Trincucci G, Gruber AJ, Vogt JE, Filipowicz M, et al. Pegylated IFN-alpha regulates hepatic gene expression through transient Jak/STAT activation. J Clin Invest 2014;124:1568-1581.

[21] Jansen L, de Niet A, Makowska Z, Dill MT, van Dort KA, Terpstra V, et al. An intrahepatic transcriptional signature of enhanced immune activity predicts response to peginterferon in chronic hepatitis B. Liver Int 2015;35:1824-1832.

[22] Carvalho BS, Irizarry RA. A framework for oligonucleotide microarray preprocessing. Bioinformatics 2010;26:2363-2367.

[23] Kleiner DE, Brunt EM, Van Natta M, Behling C, Contos MJ, Cummings OW, et al. Design and validation of a histological scoring system for nonalcoholic fatty liver disease. Hepatology 2005;41:1313-1321.

[24] Brunt EM, Janney CG, Di Bisceglie AM, Neuschwander-Tetri BA, Bacon BR. Nonalcoholic steatohepatitis: a proposal for grading and staging the histological lesions. Am J Gastroenterol 1999;94:2467-2474.

[25] Lee JH, Kim D, Kim HJ, Lee CH, Yang JI, Kim W, et al. Hepatic steatosis index: a simple screening tool reflecting nonalcoholic fatty liver disease. Dig Liver Dis 2010;42:503-508.

[26] Eslam M, Sanyal AJ, George J. MAFLD: A Consensus-Driven Proposed Nomenclature for Metabolic Associated Fatty Liver Disease. Gastroenterology 2020;158:1999-2014.e1991.

[27] Eslam M, Newsome PN, Sarin SK, Anstee QM, Targher G, Romero-Gomez M, et al. A new definition for metabolic dysfunction-associated fatty liver disease: An international expert consensus statement. J Hepatol 2020;73:202-209.

[28] Poynard T, Bedossa P, Opolon P. Natural history of liver fibrosis progression in patients with chronic hepatitis C. The OBSVIRC, METAVIR, CLINIVIR, and DOSVIRC groups. Lancet 1997;349:825-832.

Supplementary Figures

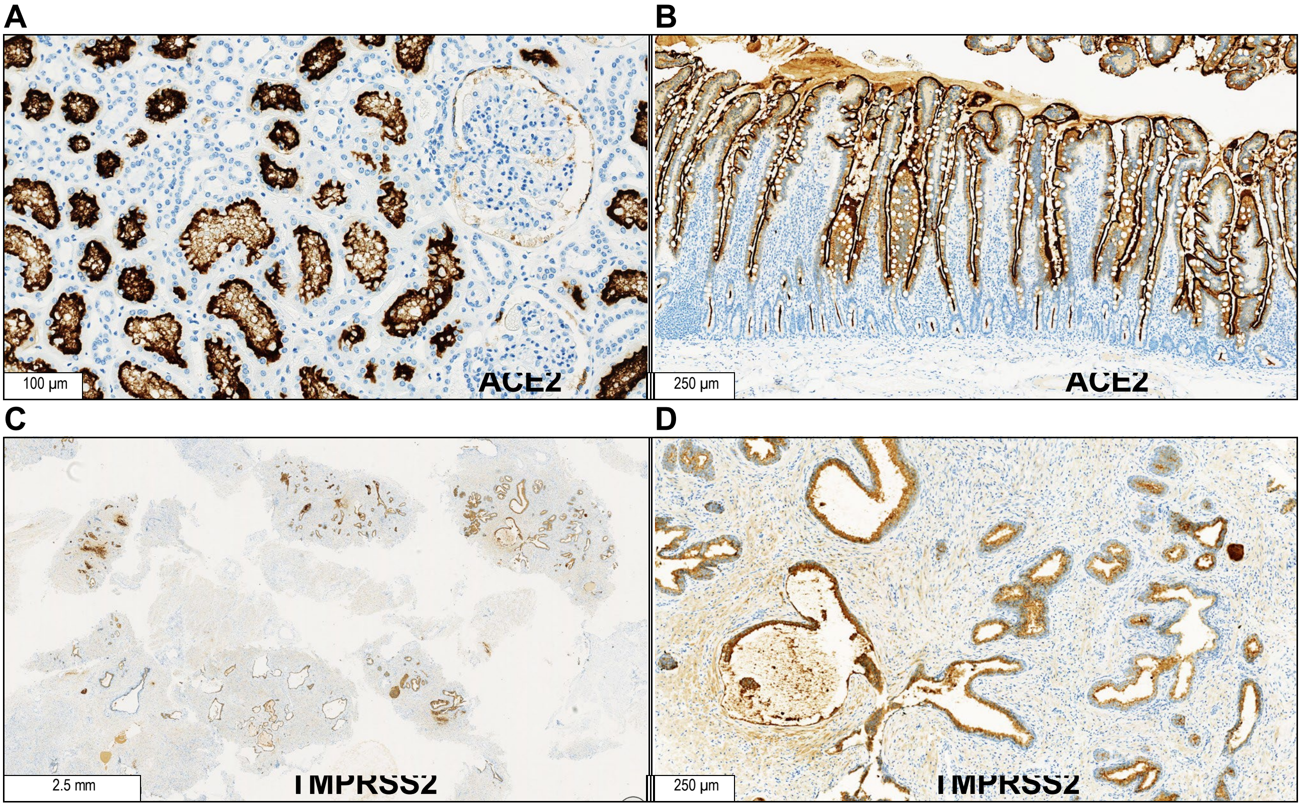

Cano et al., Fig. S1

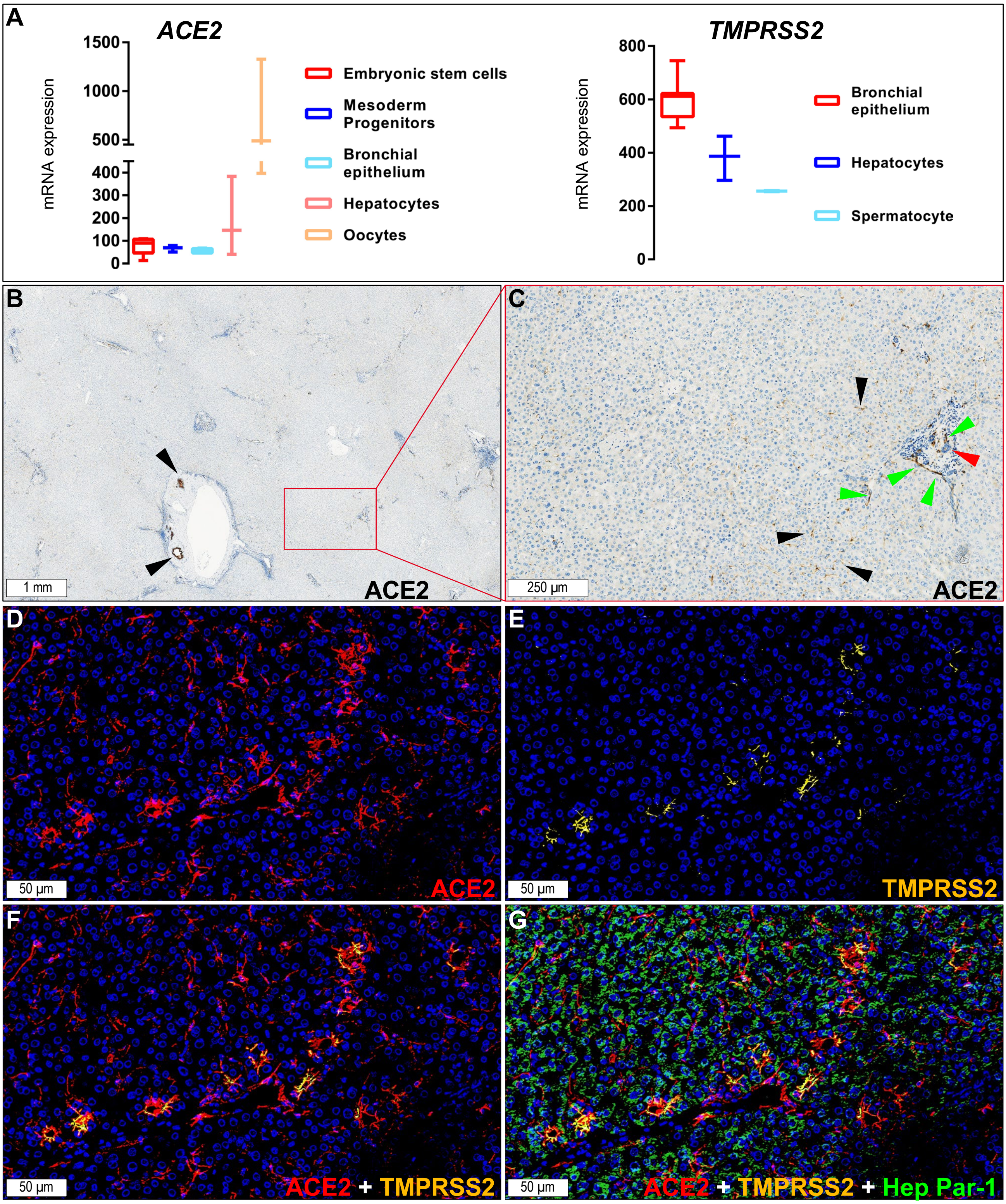

Cano et al., Fig. S2

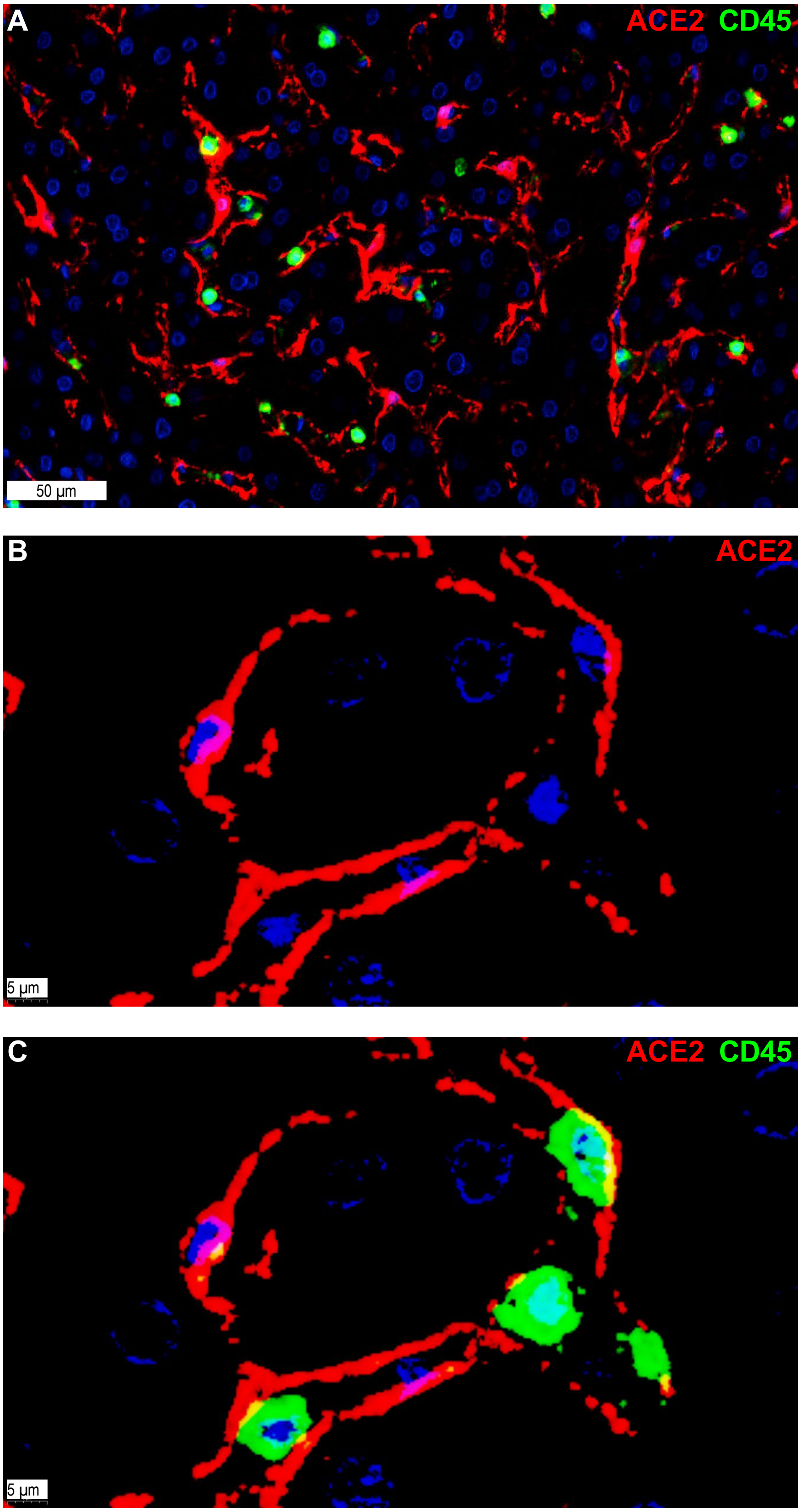

Cano et al., Fig. S3

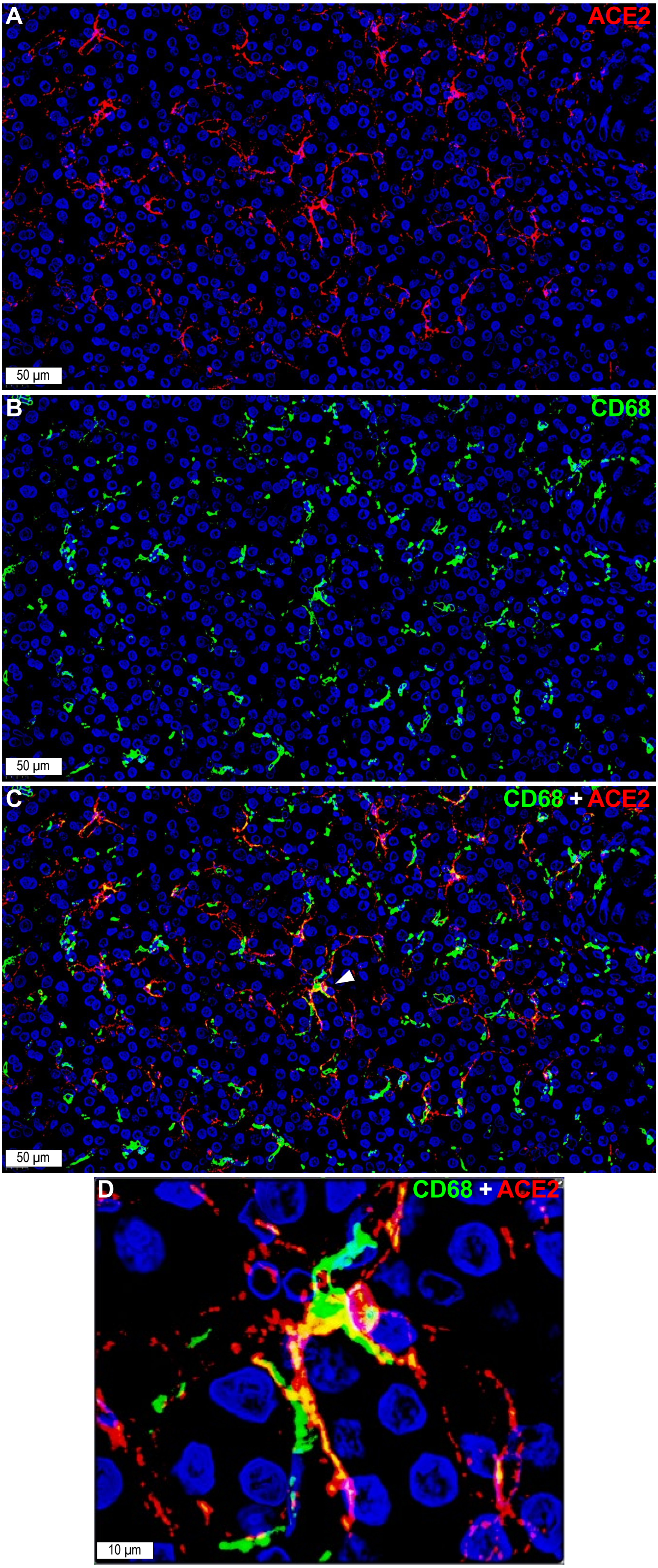

Cano et al., Fig. S4A-D

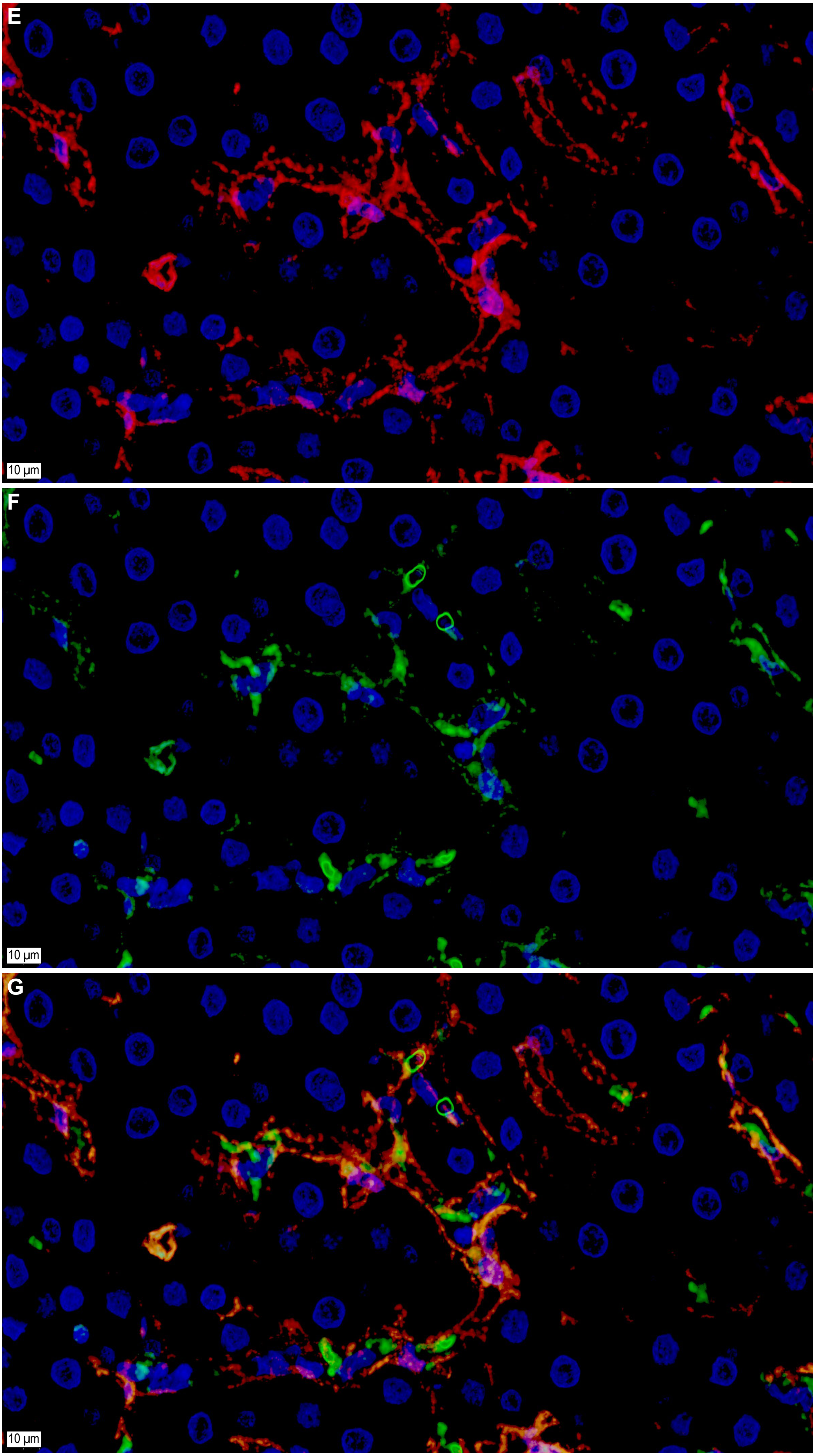

Cano et al., Fig. S4E-G

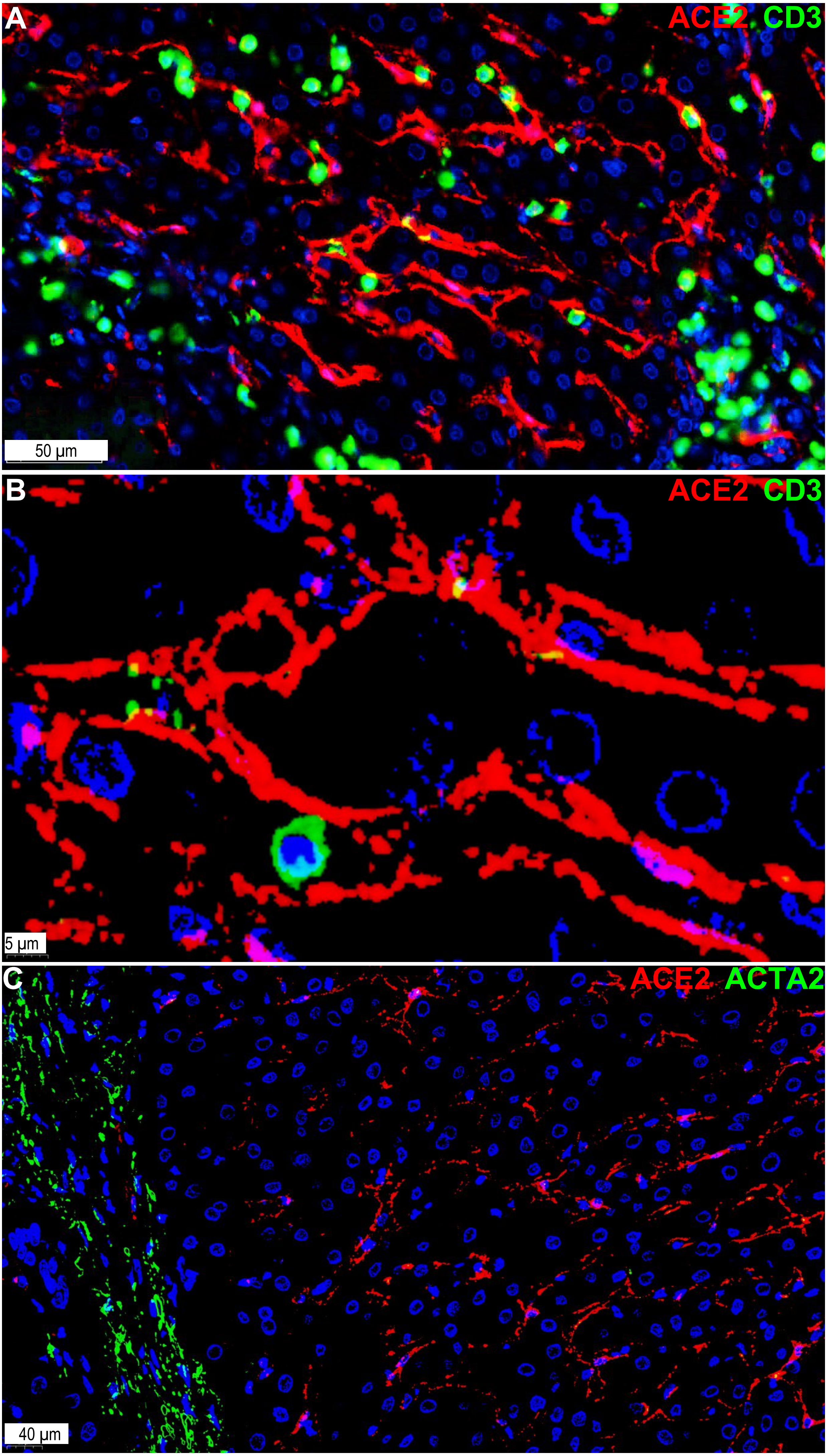

Cano et al., Fig. S5

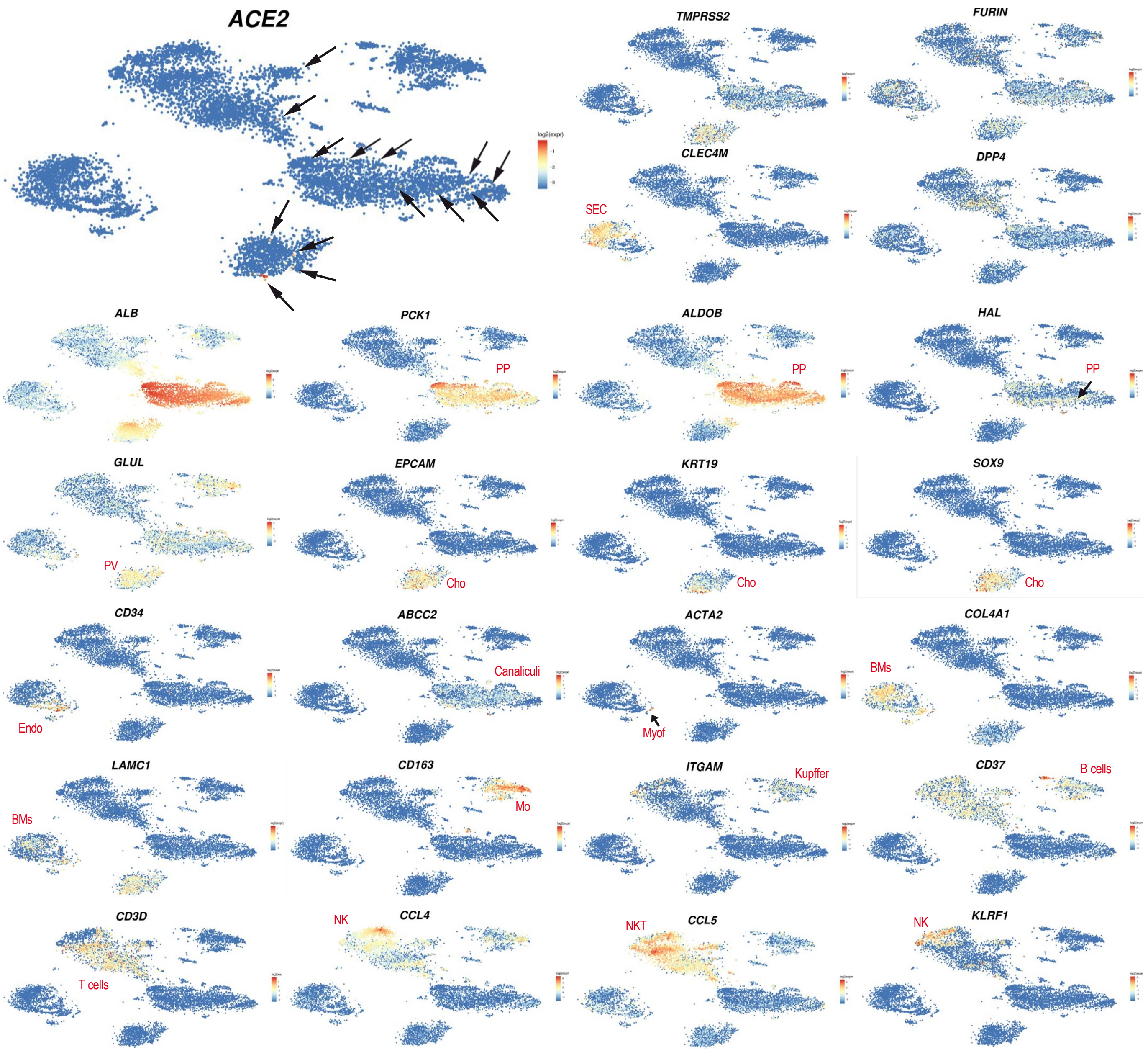

Cano et al., Fig. S6

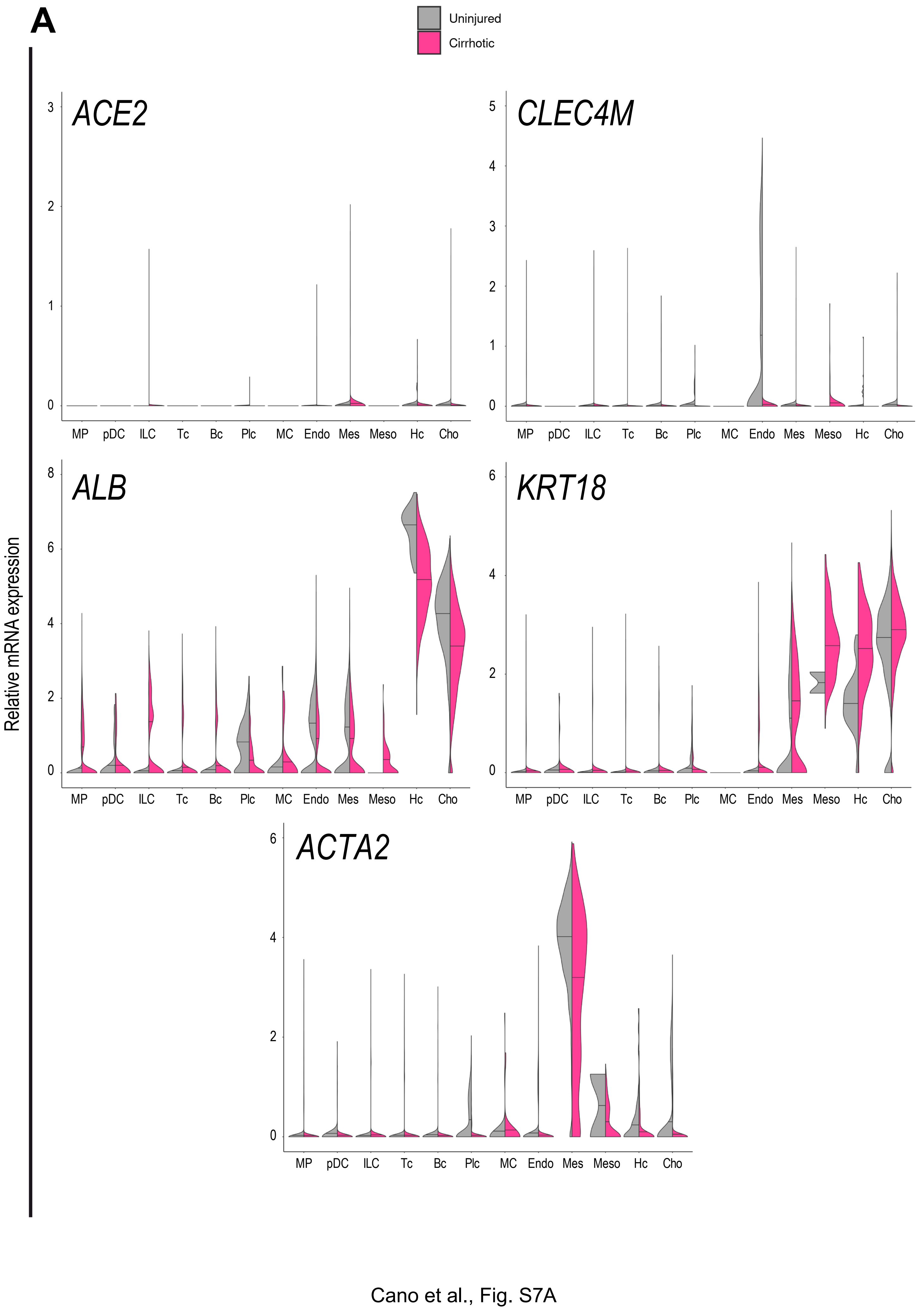

Cano et al., Fig. S7A

B

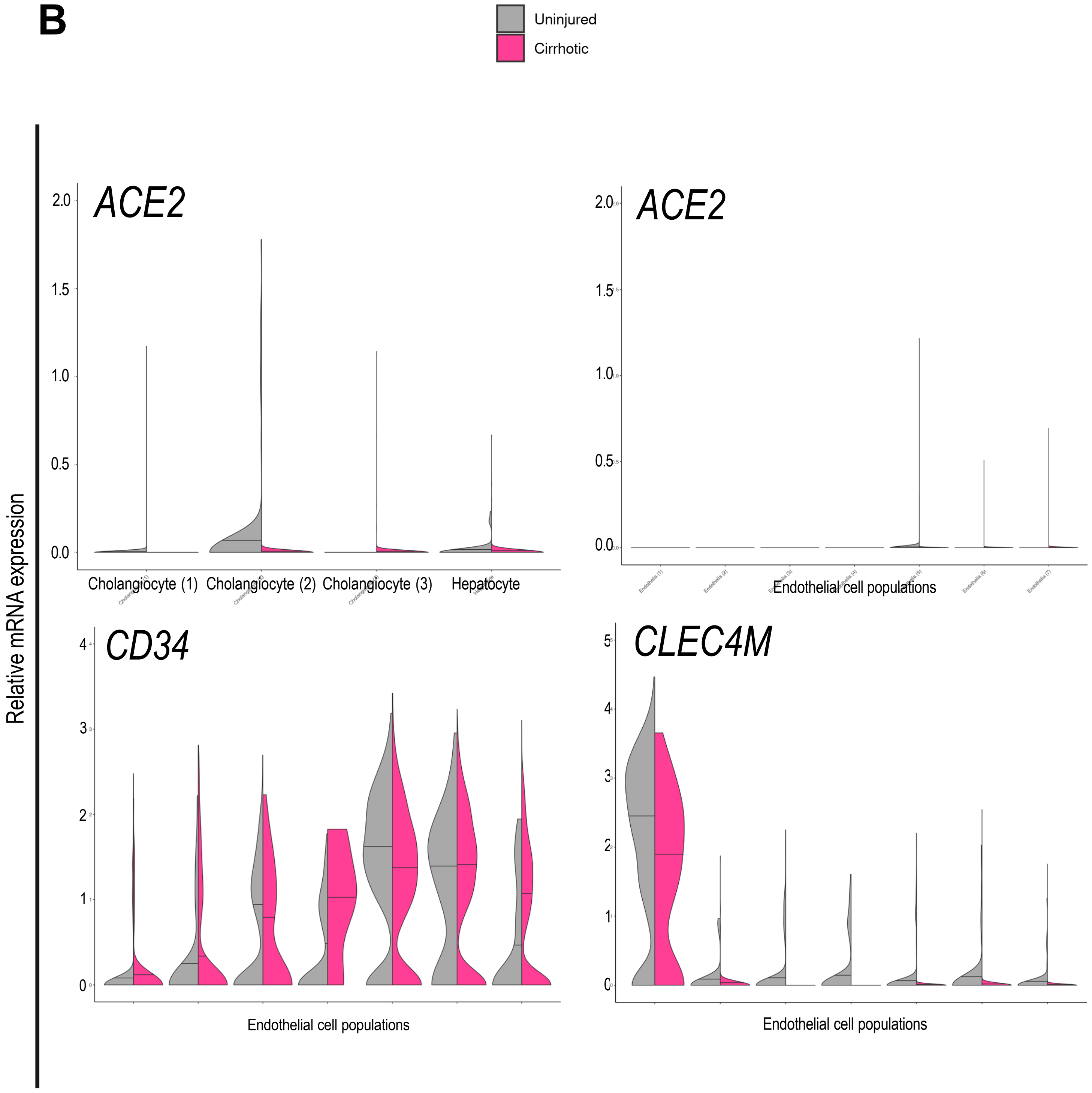

Cano et al., Fig. S7B

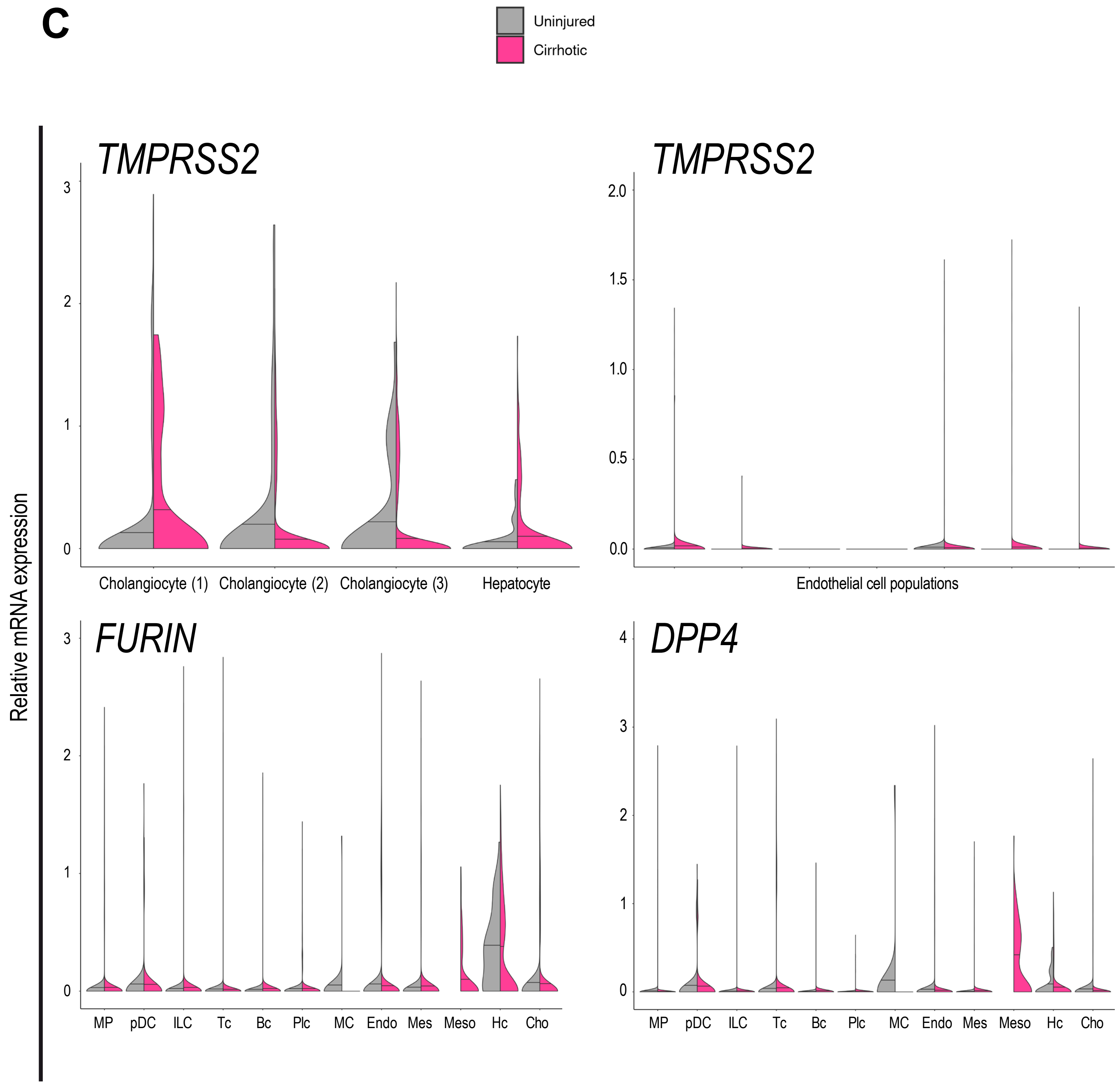

Cano et al., Fig. S7C

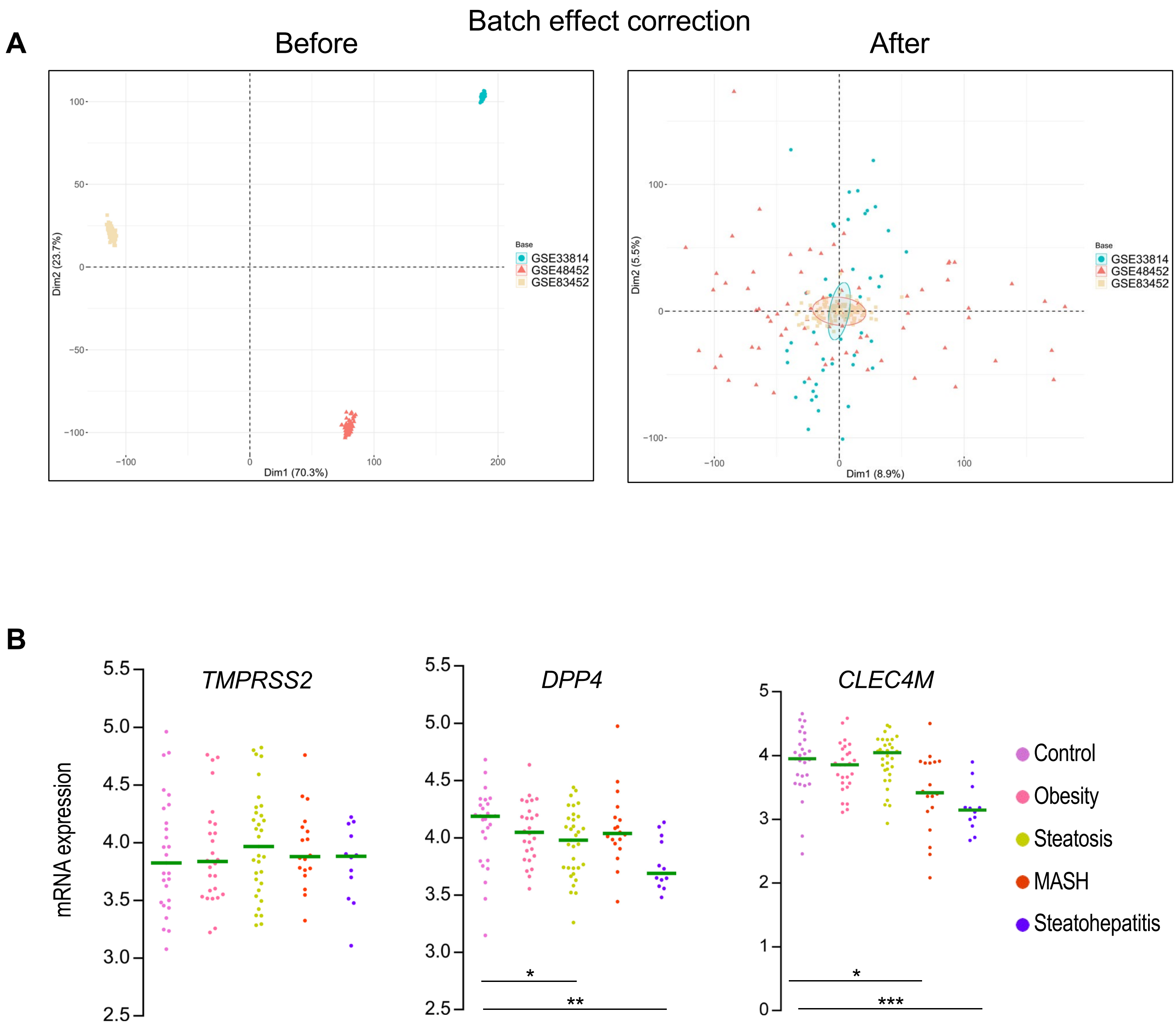

Cano et al., Fig. S8

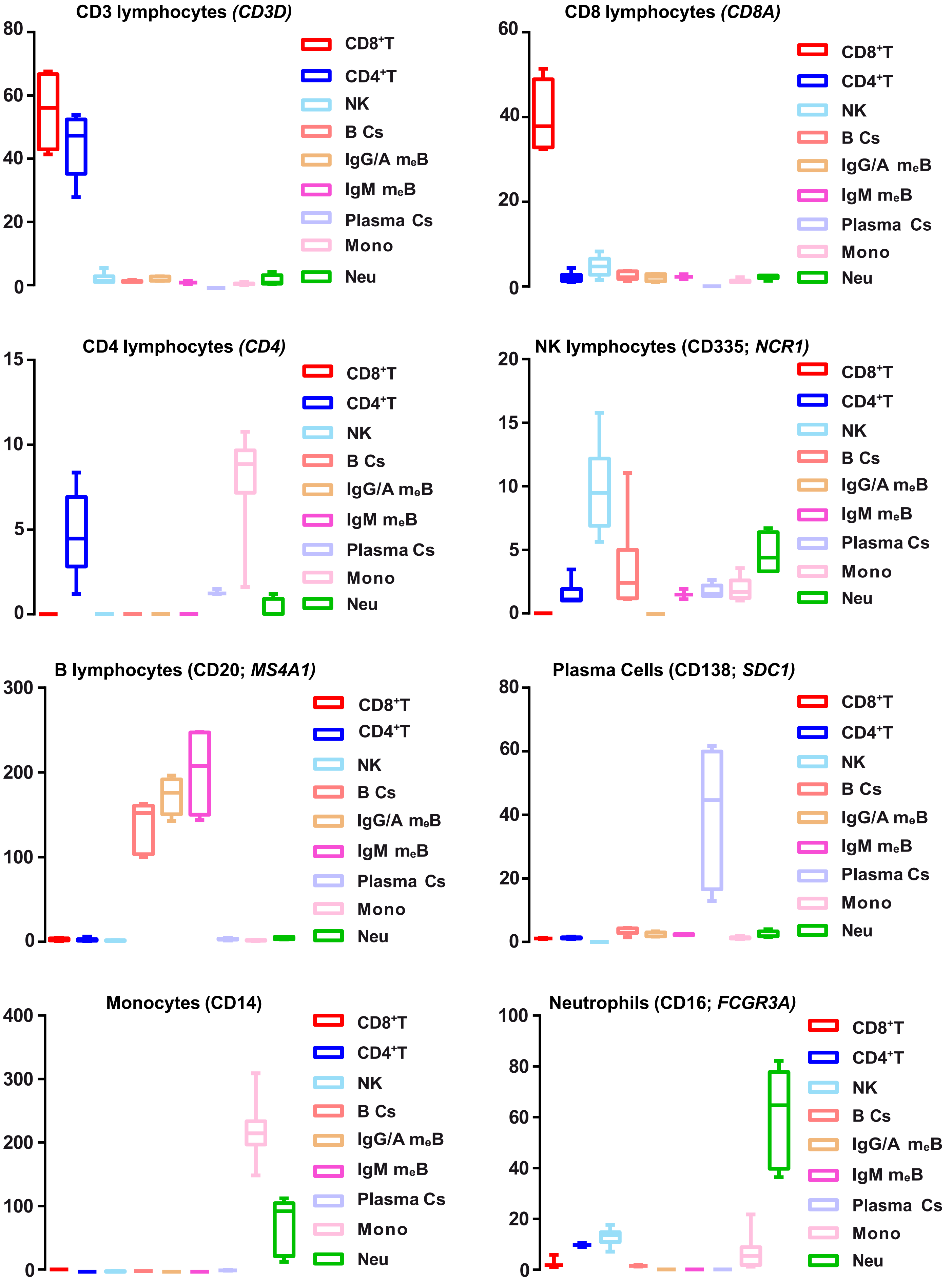

Cano et al., Fig. S9

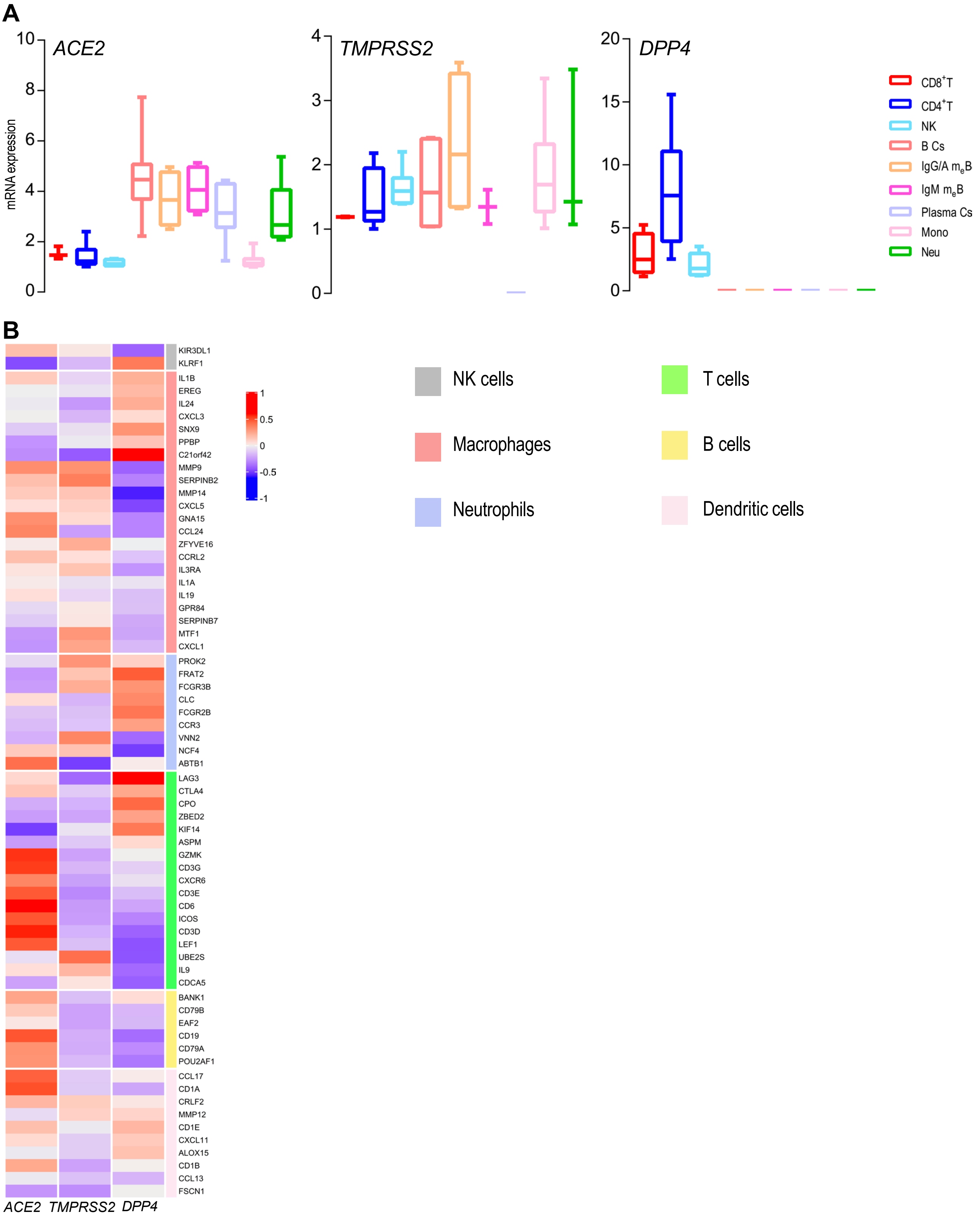

Cano et al., Fig. S10

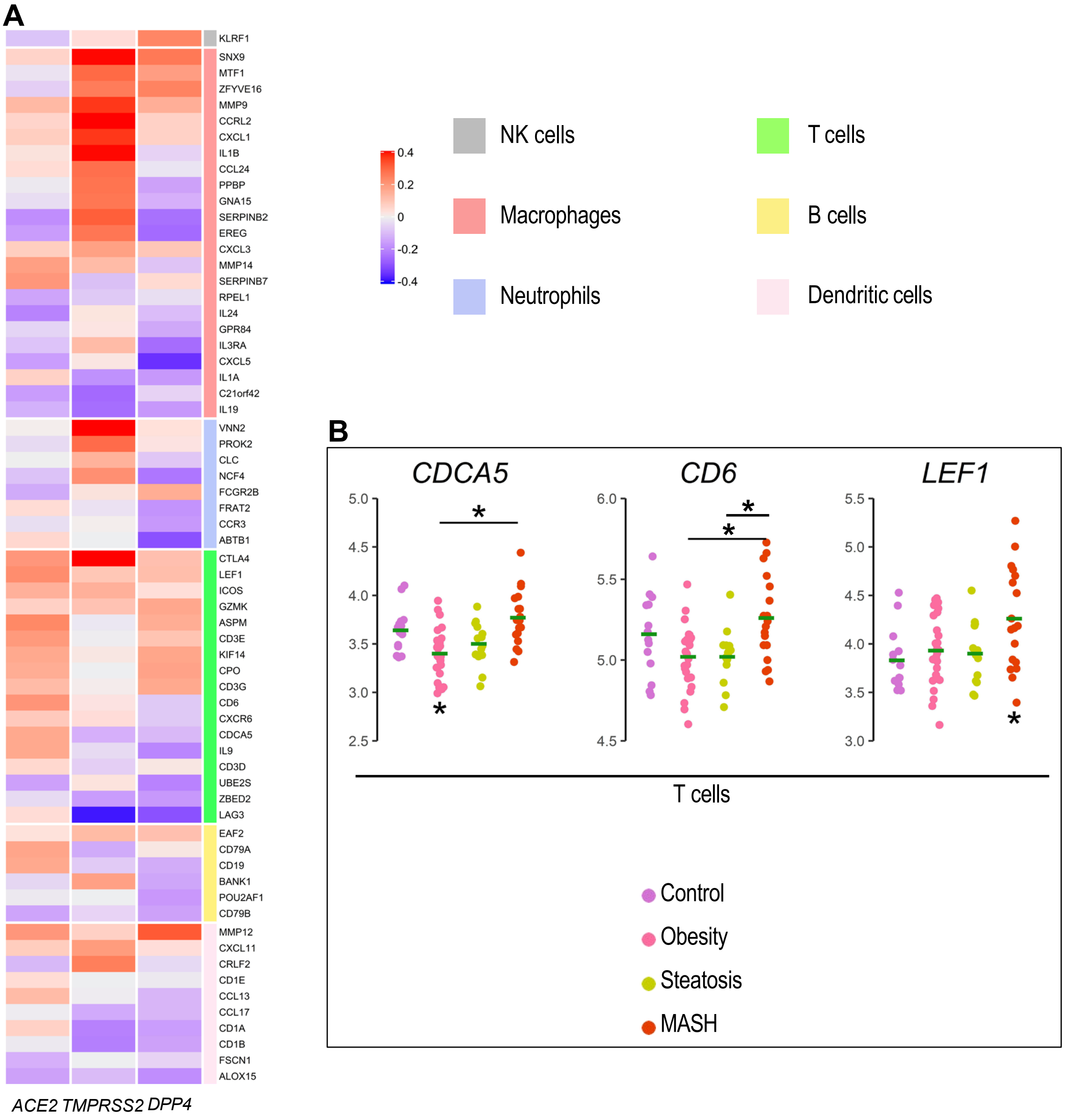

Cano et al., Fig. S11

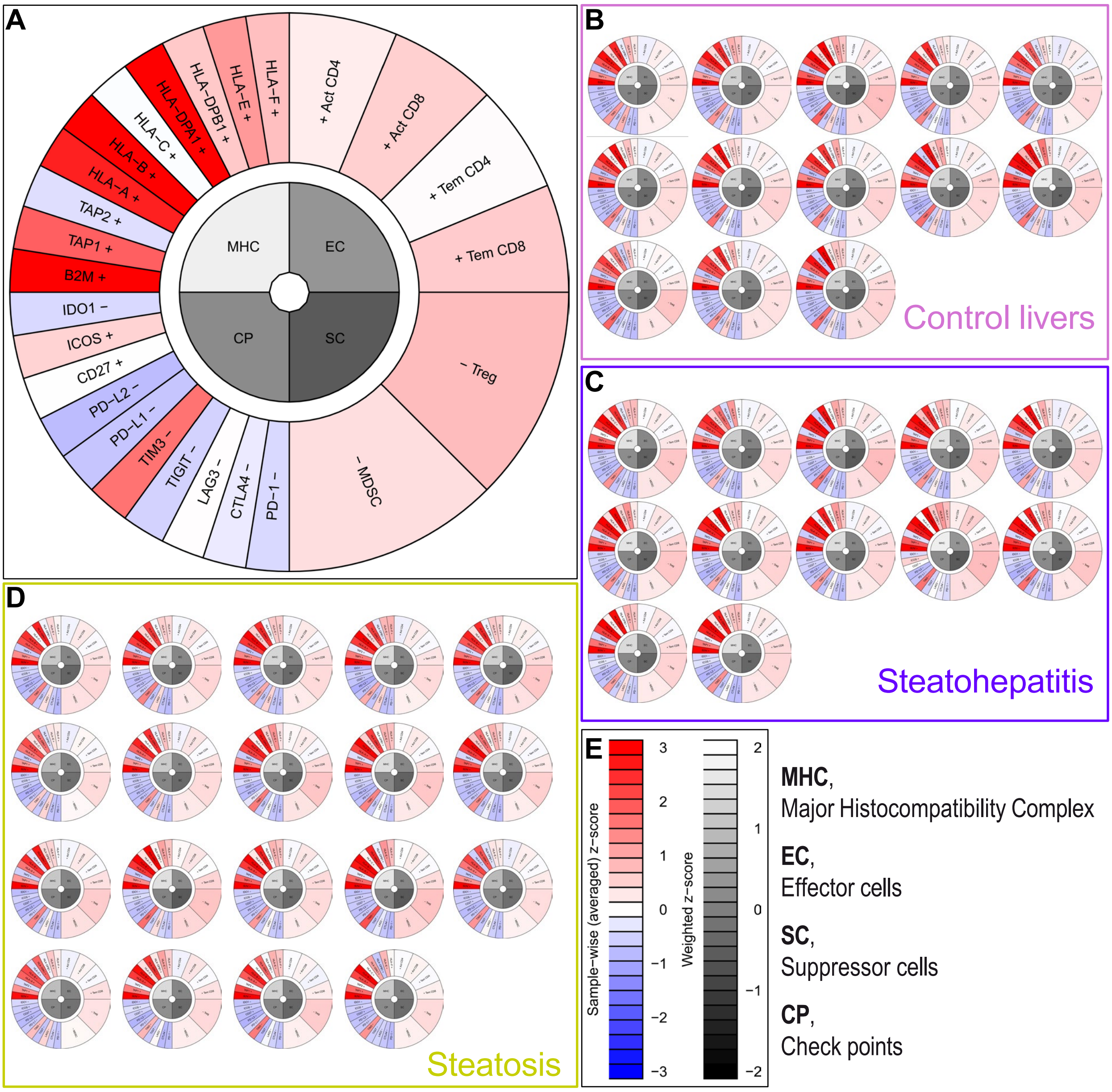

Cano et al., Fig. S12

Eigengene Network

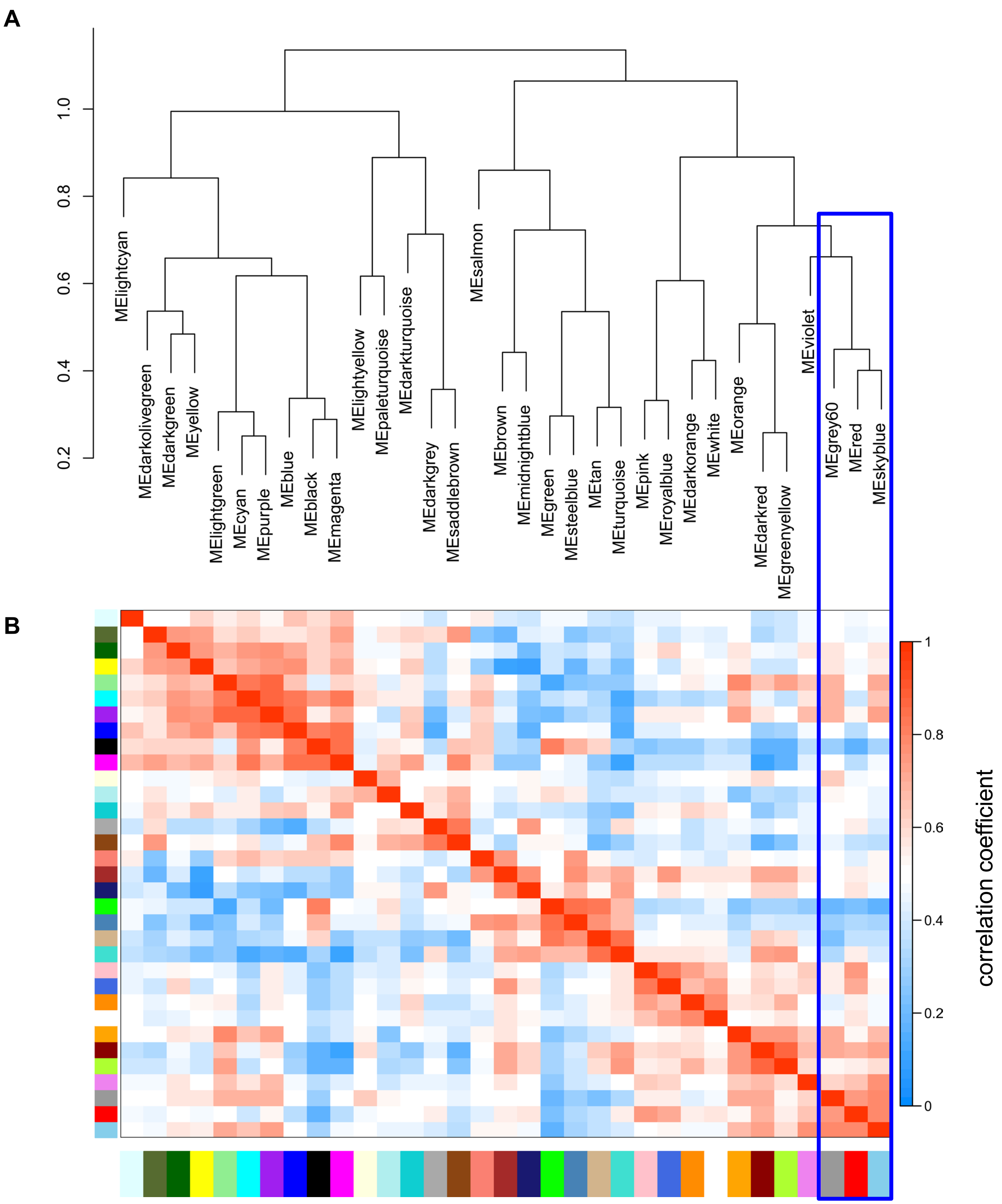

Cano et al., Fig. S13

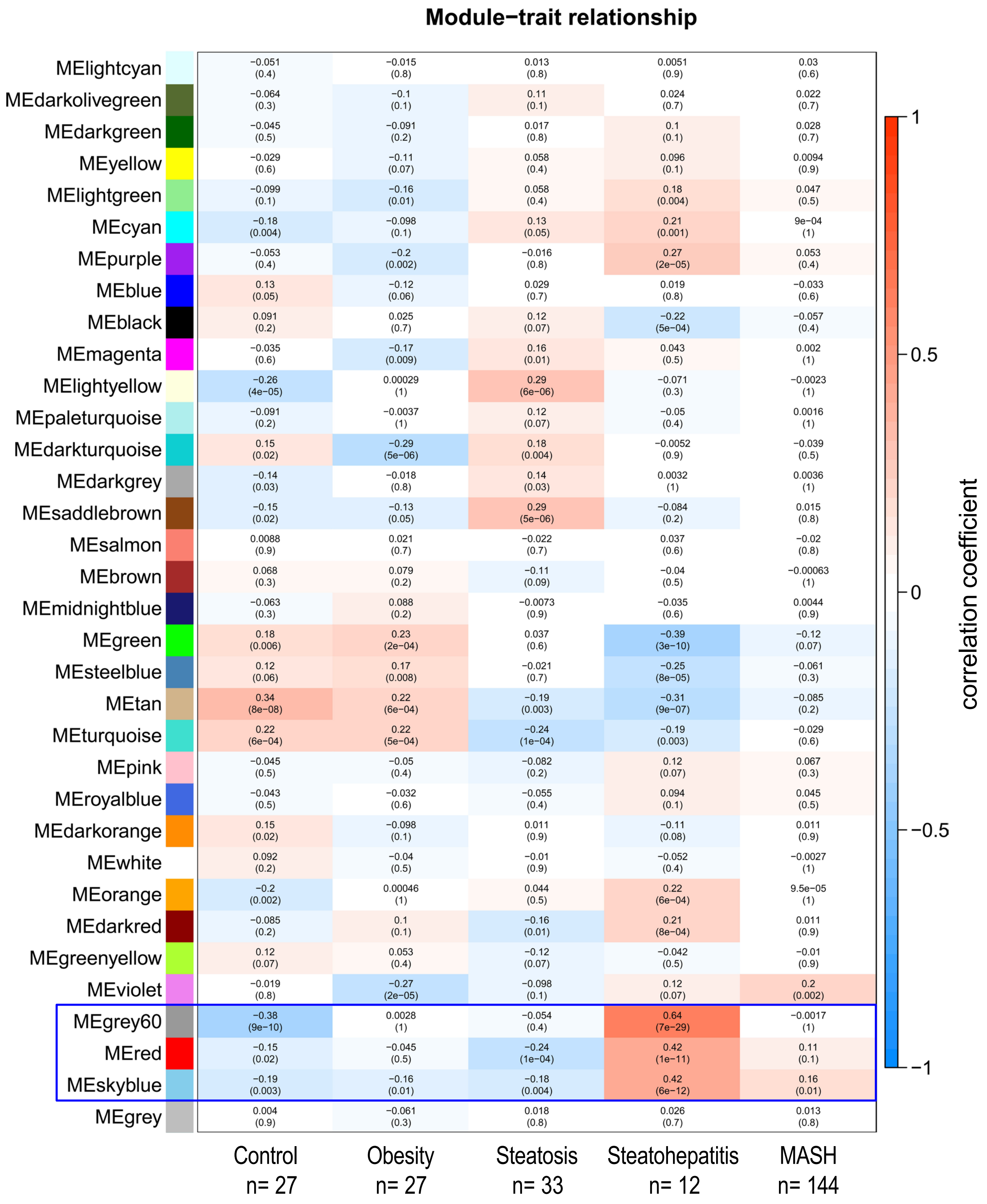

Cano et al., Fig. S14

Module Grey 60

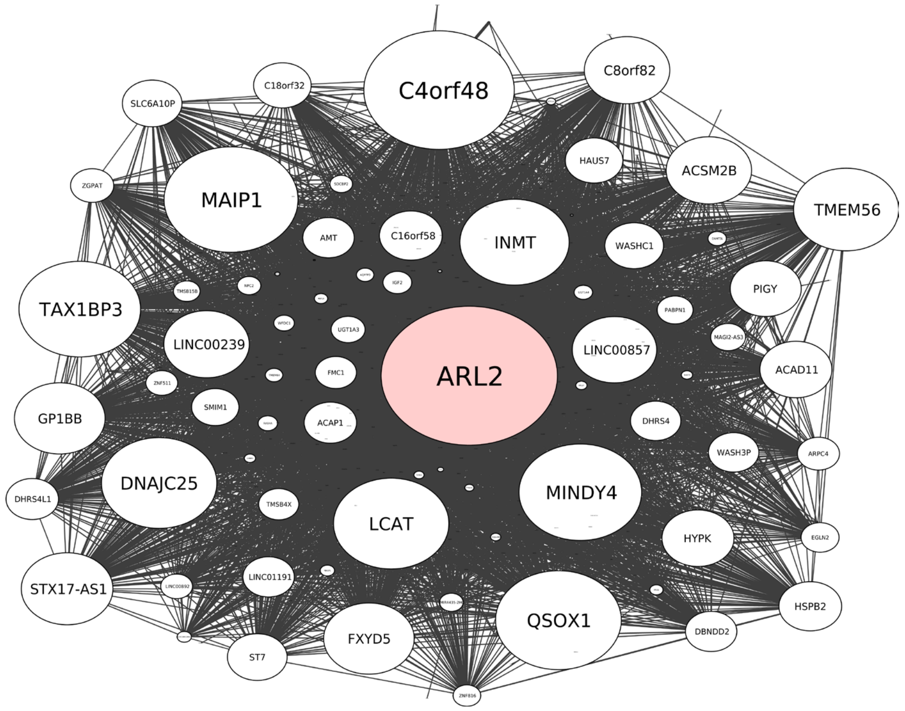

Module Red

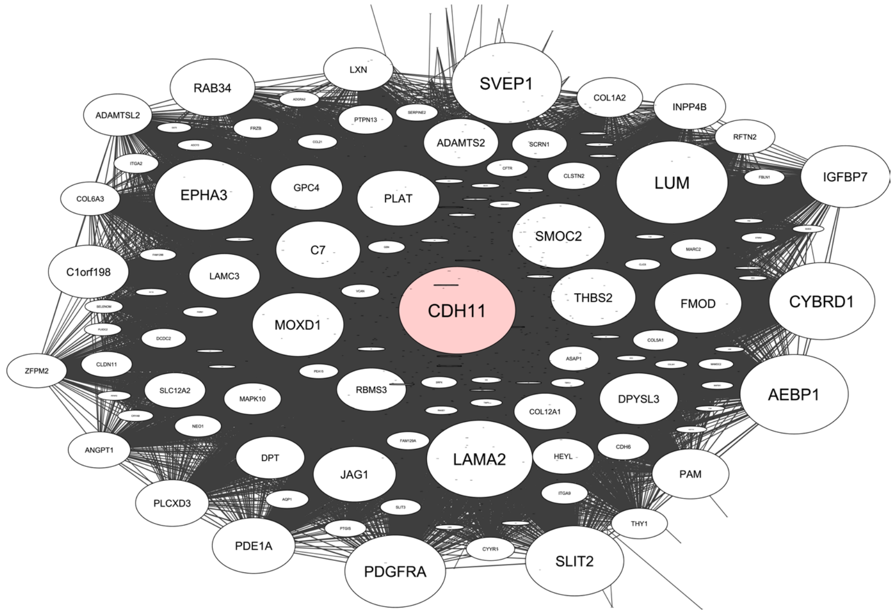

Module Skyblue

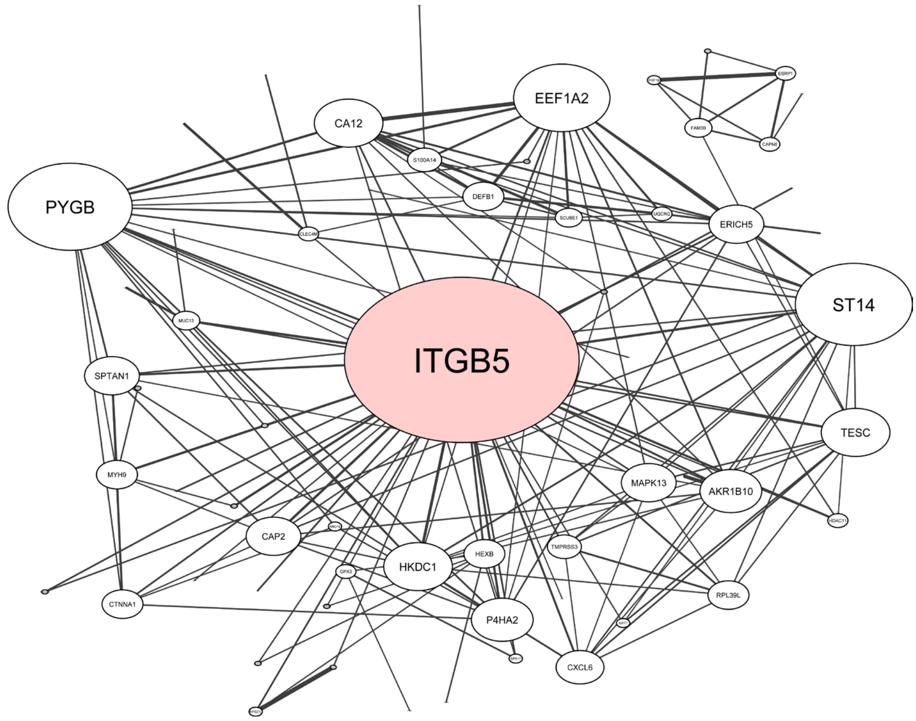

Cano et al., Fig. S15

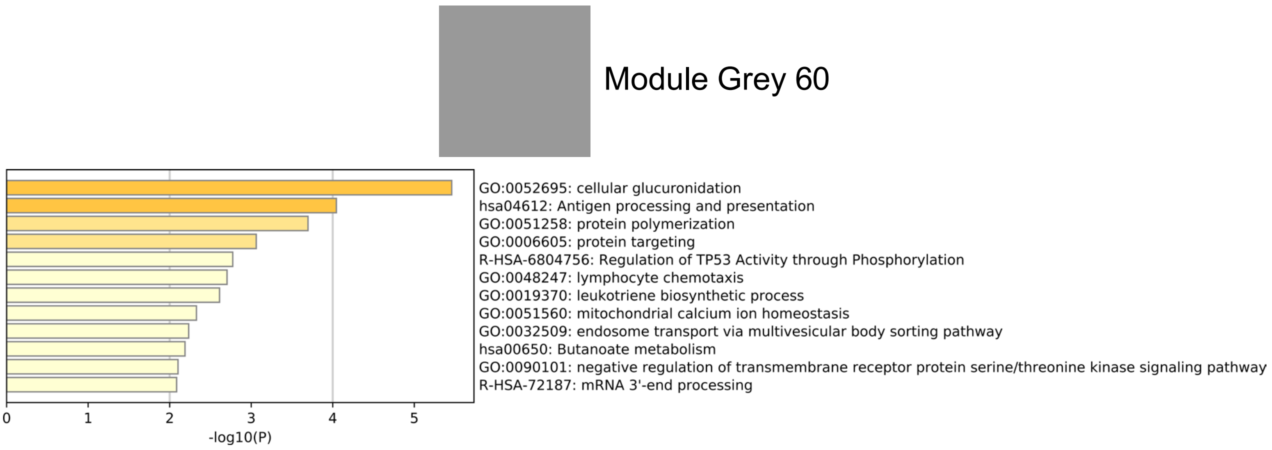

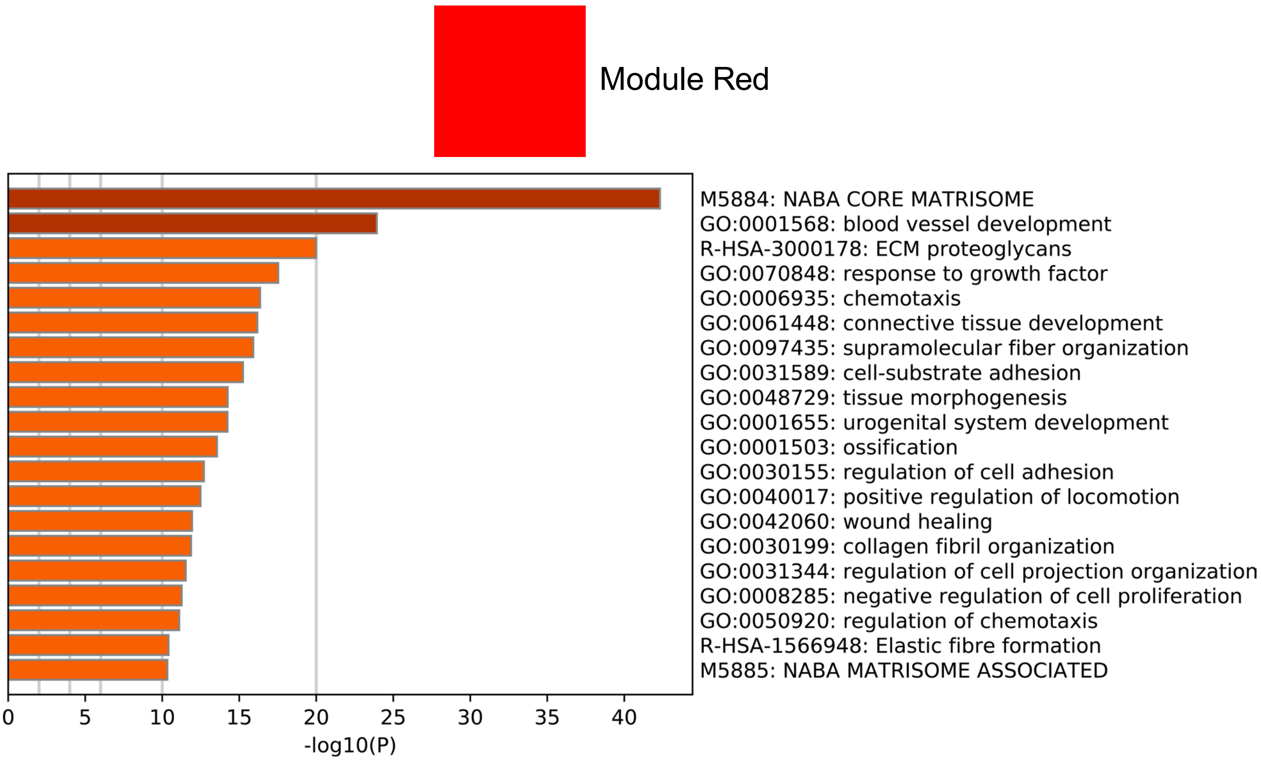

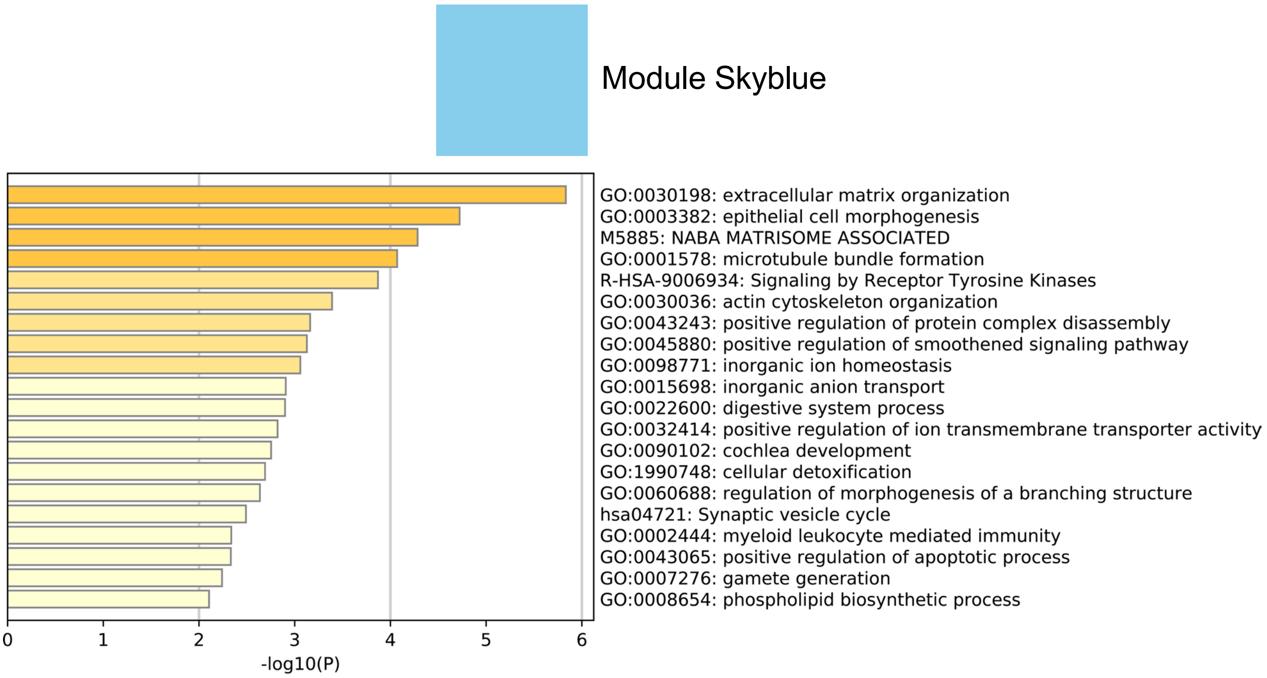

Cano et al., Fig. S16

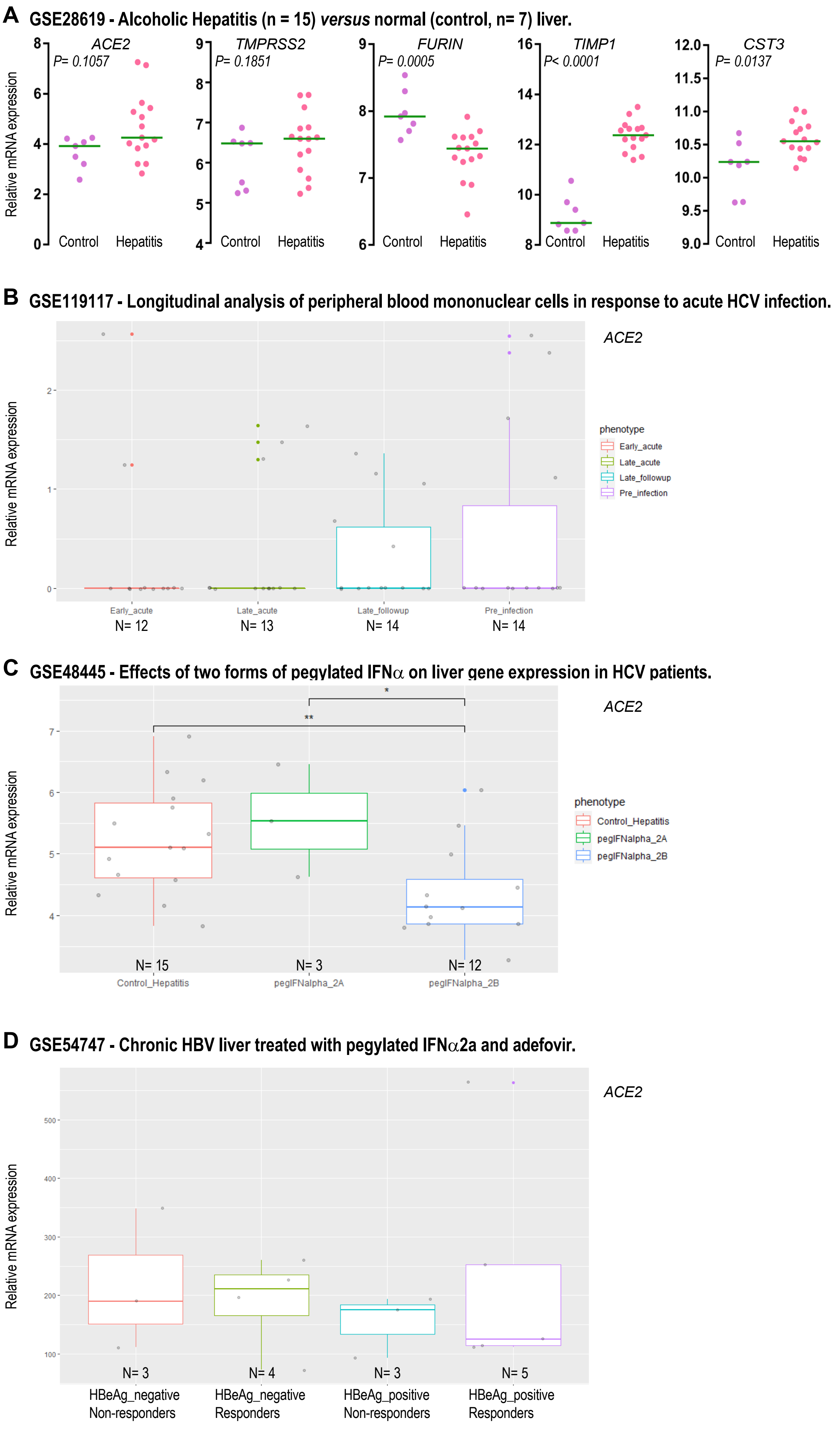

Cano et al., Fig. S17

Primary human hepatocytes & cytokines *in vitro*

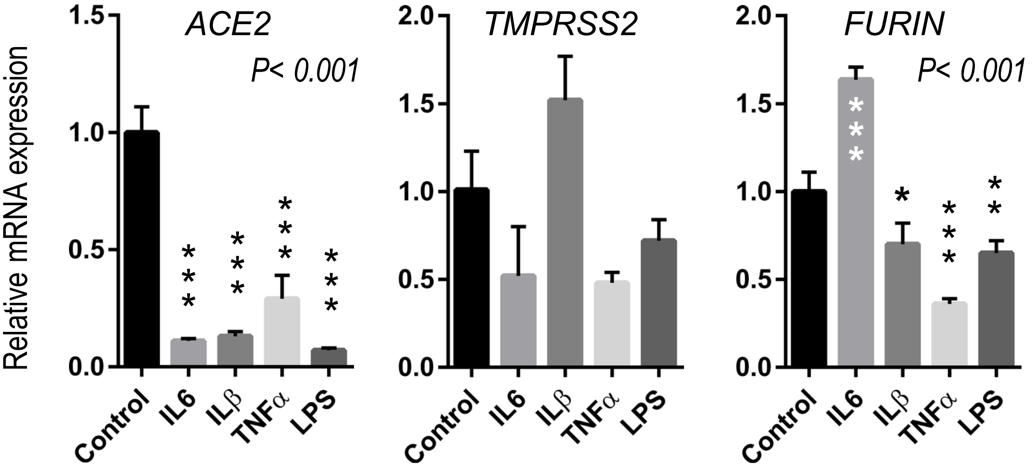

Cano et al., Fig. S18

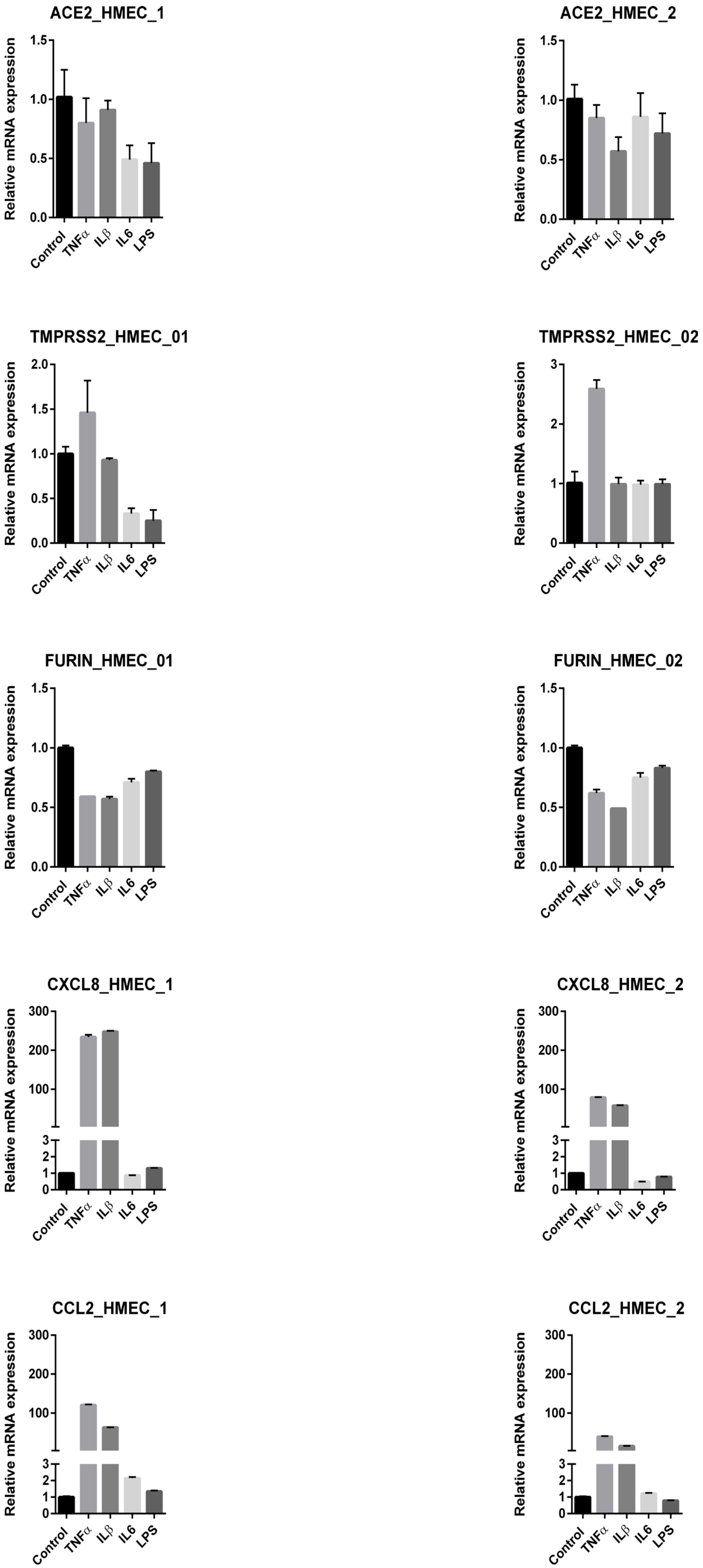

Cano et al., Fig. S19

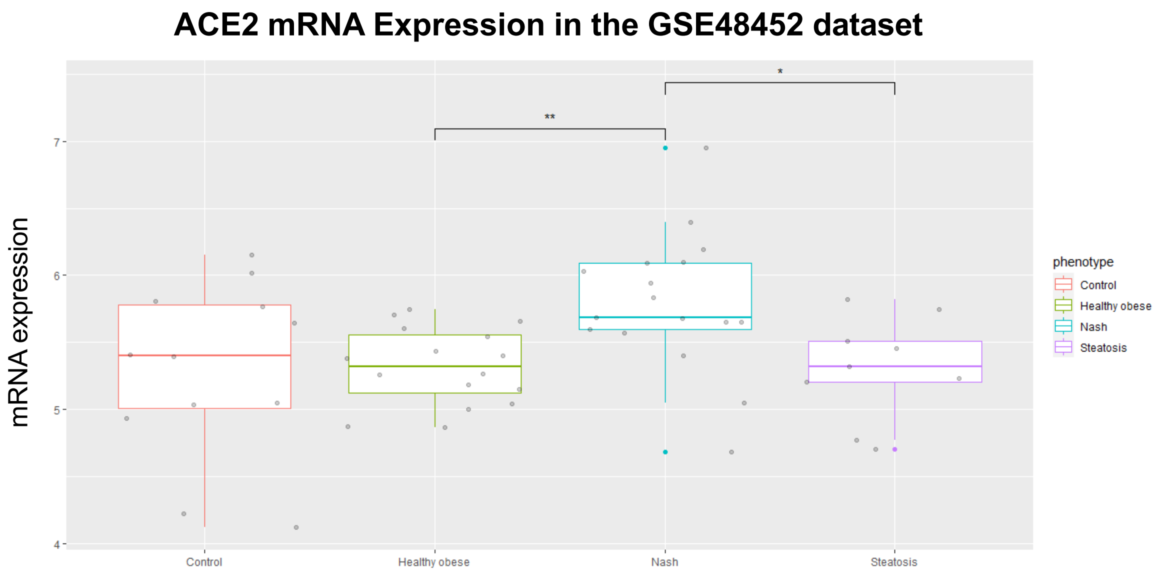

Cano et al., Fig. S20

Supplementary Tables

Table S4

	GROUPS				
**GENE SYMBOL**	**Control**	**Obesity**	**Steatosis**	**MASH**	**Steatohepatitis**
ACE2	27	27	33	18	12
COL1A1	27	27	33	18	12
COL3A1	27	27	33	18	12
VCAN	27	27	33	18	12
COL4A1	27	27	33	144	12
LAMC1	27	27	33	144	12

Table S5

Immunogenicity functional families			
**Major Histocompatibility Complex (MHC)**	**Effector Cells (EC)**	**Suppressor Cells (SC)**	**Check Points (CP)**
B2M, Beta-2-Microglobulin	Act CD4, Activated CD4 T cells	T reg, T regulatory cells	PD-1, Programmed cell death 1
TAP1, Transporter 1, ATP Binding Cassette Subfamily B Member	Act CD8, Activated CD8 T cells	MDSC, Myeloid-derived suppressor cells	CTLA4, Cytotoxic T-Lymphocyte Associated Protein 4
TAP2, Transporter 2, ATP Binding Cassette Subfamily B Member	Tem CD4, T effector memory CD4 cells		LAG3, Lymphocyte Activating 3
HLA-A, Major Histocompatibility Complex, Class I, A	Tem CD8, T effector memory CD8 cells		TIGIT, T Cell Immunoreceptor With Ig And ITIM Domains
HLA-B, Major Histocompatibility Complex, Class I, B			TIM3, Hepatitis A Virus Cellular Receptor 2 (HAVCR2)
HLA-C, Major Histocompatibility Complex, Class I, C			PD-L1, Programmed Death Ligand 1 (CD274)
HLA-DPA1, Major Histocompatibility Complex, Class II, DP Alpha 1			PD-L2, Programmed Cell Death 1 Ligand 2 (PDCD1LG2)
HLA-DPB1, Major Histocompatibility Complex, Class II, DP Beta 1			CD27, T-Cell Activation Antigen CD27
HLA-E, Major Histocompatibility Complex, Class I, E			ICOS, Inducible T Cell Costimulator
HLA-F, Major Histocompatibility Complex, Class I, F			IDO1, Indoleamine 2,3-Dioxygenase 1

Table S6

Category	Control Livers (C)		Steatosis (S)		Steatohepatitis (ST)		Kruskall Wallis	Dunn Test
	Mean	SD	Mean	SD	Mean	SD		
**Treg**	7,33	0,11	7,26	0,18	7,30	0,11	0,25	
**MDSC**	7,61	0,22	7,68	0,19	7,80	0,19	0,06	
**Tem CD8**	7,46	0,11	7,40	0,13	7,49	0,15	0,15	
**Tem CD4**	6,78	0,03	6,77	0,04	6,78	0,05	0,62	
**Act CD8**	7,21	0,09	7,19	0,11	7,31	0,14	0,08	
**Act CD4**	6,68	0,09	6,73	0,14	6,82	0,15	**0,04**	**0.03 (C-ST)**
**IDO1**	5,98	0,22	5,90	0,22	5,94	0,14	0,56	
**ICOS**	5,93	0,07	6,06	0,13	6,31	0,44	**< 0.01**	**<0.01 (C-ST)**<br>**0.02 (C-S)**<br>**0.04 (S-ST)**
**CD27**	5,78	0,09	5,75	0,14	5,98	0,29	**< 0.01**	
**PD-L2**	5,62	0,13	5,55	0,12	5,53	0,08	0,14	
**PD-L1**	5,78	0,09	5,85	0,16	5,77	0,06	0,27	
**TIM3**	9,28	0,36	8,92	0,56	8,89	0,44	0,08	
**TIGIT**	5,95	0,07	5,86	0,10	5,87	0,10	**0,03**	**0.03 (C-S)**
**LAG3**	7,28	0,52	7,11	0,47	6,87	0,41	0,11	
**CTLA 4**	5,94	0,13	5,84	0,14	5,90	0,21	0,21	
**PD-1**	5,69	0,05	5,68	0,08	5,71	0,16	0,91	
**HLA-F**	7,29	0,38	7,56	0,42	7,76	0,40	**0,02**	**0.01 (C-ST)**
**HLA-E**	8,17	0,50	8,10	0,46	8,39	0,37	0,18	
**HLA-DPB1**	6,80	0,43	6,74	0,45	6,94	0,47	0,41	
**HLA-DPA1**	10,90	0,69	10,52	0,80	11,27	0,75	**0,02**	**0.01 (S-ST)**
**HLA-C**	7,13	0,67	7,16	0,54	7,13	0,56	0,96	
**HLA-B**	11,15	0,42	11,36	0,69	11,90	0,48	**< 0.01**	**<0.01 (C-ST)**<br>**0.04 (S-ST)**
**HLA-A**	9,96	0,53	10,25	0,50	10,81	0,33	**< 0.01**	**<0.01 (C-ST)**<br>**0.02 (S-ST)**
**TAP2**	5,91	0,17	6,12	0,30	6,23	0,25	**< 0.01**	**<0.01 (C-ST)**
**TAP1**	9,16	0,45	9,32	0,46	9,61	0,43	0,07	
**B2M**	12,67	0,29	12,26	0,37	12,47	0,26	**0,01**	**< 0.01(C-S)**

Table S9

	Immune cell subsets								
**GENE SYMBOL**	**$CD8^+T$**	**$CD4^+T$**	**NK**	**B Cells**	**IgG/A m_eB**	**IgM m_eB**	**Plasma Cells**	**Monocytes**	**Neutrophiles**
ACE2	3	10	4	7	4	4	7	13	5
TMPRSS2	2	11	6	7	4	2	0	22	3
DPP4	4	20	8	0	0	0	0	0	0
