# Supplementary material for: SARS-CoV-2 receptor ACE2 is upregulated by fatty acids in human MASH
Source: JHEP Rep. 2023 Oct 13;6(1):100936. doi: 10.1016/j.jhepr.2023.100936 (PMC10698276; doi:10.1016/j.jhepr.2023.100936)
Supplement: Multimedia component 7 [file mmc7.docx]

**Journal of Hepatology**

**CTAT methods**

Tables for a “Complete, Transparent, Accurate and Timely account” (CTAT) are now mandatory for all revised submissions. The aim is to enhance the reproducibility of methods.

- Only include the parts relevant to your study
- Refer to the CTAT in the main text as ‘Supplementary CTAT Table’
- Do not add subheadings
- Add as many rows as needed to include all information
- Only include one item per row

**If the CTAT form is not relevant to your study, please outline the reasons why:**

| **Patients, materials and methods** addressed in this study have been described in the relevant parts of the manuscript so as to make them readily accessible to the readers.  **Antibodies and immunohistochemichal procedures** are detailed in Supplementary Table 2.  **Cell lines**: we didn’t use cell lines, but primary human hepatocyte cultures from 6 patients. These are described in the Supplementary Methods section.  **Patients** are described in the Patients and Methods section of the main manuscript. Microarray, RNA-seq and scRNA-seq datasets used in this study are publicly available and have been referred to by the their GSE ID and bibliographic reference.  **Clinical data from patients** are shown in Supplementary Tables 1 and 8 and referred to when appropriate. Their presentation in an excel (.xlsx) format is more user friendly if readers wish to export and reanalyse these data with commonly used software, such as R, while exports from Word tables can be rather tricky.  **Sequence based reagents, such as PCR primers** are detailed in Supplementary Table 3.  **Software** is detailed in the Supplementary Methods section.  **Recombinant cytokines, LPS and long chain fatty acids** are described in the Supplementary Methods section. |
| --- |

- 1. **Antibodies**

| **Name** | **Citation** | **Supplier** | **Cat no.** | **Clone no.** |
| --- | --- | --- | --- | --- |
|  |  |  |  |  |

- 1. **Cell lines**

| **Name** | **Citation** | **Supplier** | **Cat no.** | **Passage no.** | **Authentication test method** |
| --- | --- | --- | --- | --- | --- |
|  |  |  |  |  |  |

- 1. **Organisms**

| **Name** | **Citation** | **Supplier** | **Strain** | **Sex** | **Age** | **Overall n number** |
| --- | --- | --- | --- | --- | --- | --- |
|  |  |  |  |  |  |  |

- 1. **Sequence based reagents**

| **Name** | **Sequence** | **Supplier** |
| --- | --- | --- |
|  |  |  |

- 1. **Biological samples**

| **Description** | **Source** | **Identifier** |
| --- | --- | --- |
|  |  |  |

- 1. **Deposited data**

| **Name of repository** | **Identifier** | **Link** |
| --- | --- | --- |
|  |  |  |

- 1. **Software**

| **Software name** | **Manufacturer** | **Version** |
| --- | --- | --- |
|  |  |  |

- 1. **Other (e.g. drugs, proteins, vectors etc.)**

|  |  |  |
| --- | --- | --- |
|  |  |  |

- 1. **Please provide the details of the corresponding methods author for the manuscript:**

| The corresponding methods author is the corresponding author: orlando.musso@inserm.fr |
| --- |

**2.0 Please confirm for randomised controlled trials all versions of the clinical protocol are included in the submission. These will be published online as supplementary information.**

| **Not applicable.** |
| --- |
