# Supplementary material for: SARS-CoV-2 receptor ACE2 is upregulated by fatty acids in human MASH
Source: JHEP Rep. 2023 Oct 13;6(1):100936. doi: 10.1016/j.jhepr.2023.100936 (PMC10698276; doi:10.1016/j.jhepr.2023.100936)
Supplement: Multimedia component 9 [file mmc9.pdf]

Research article

JHEP|Reports

# SARS-CoV-2 receptor ACE2 is upregulated by fatty acids in human MASH

**Luis Cano**,[1,†] **Lise Desquilles**,[1,†] Gevorg Ghukasyan,[2] Gaëlle Angenard,[1] Clémence Landreau,[1] Anne Corlu,[1] Bruno Clément,[1] Bruno Turlin,[1] Eric Le Ferrec,[3] Caroline Aninat,[1] Julie Massart,[1] Orlando Musso[1,*]

*[1]INSERM, INRAE, Univ Rennes 1, Nutrition Metabolisms and Cancer, Rennes, France; [2]Univ Rennes 1, CNRS, INSERM, UMS Biosit, Core Facility H2P2, Rennes, France; [3]Univ Rennes 1, INSERM, EHESP, IRSET (Institut de Recherche en Santé Environnement et Travail) UMR_S 1085, Rennes, France*

JHEP Reports **2024.** https://doi.org/10.1016/j.jhepr.2023.100936

**Background & Aims:** Metabolic dysfunction-associated steatotic liver disease (MASLD) results in steatosis, inflammation (steatohepatitis), and fibrosis. Patients with MASLD more likely develop liver injury in coronavirus disease 2019 (COVID-19), caused by the severe acute respiratory syndrome coronavirus 2 (SARS-CoV-2). As viral RNA has been identified in liver tissues, we studied expression levels and cellular sources of the viral receptor angiotensin-converting enzyme 2 (ACE2) and coreceptors in MASLD and fibroinflammatory liver diseases.
**Methods:** We built a transcriptomic MASLD meta-dataset (N = 243) to study SARS-CoV-2 receptor expression and verified results in 161 additional cases of fibroinflammatory liver diseases. We assessed the fibroinflammatory microenvironment by deconvoluting immune cell populations. We studied the cellular sources of ACE2 by multiplex immunohistochemistry followed by high-resolution confocal microscopy (N = 9 fatty livers; N = 7 controls), meta-analysis of two single-cell RNA sequencing datasets (N = 5 cirrhotic livers; N = 14 normal livers), and bulk transcriptomics from 745 primary cell samples. *In vitro*, we tested *ACE2* mRNA expression in primary human hepatocytes treated with inflammatory cytokines, bacterial lipopolysaccharides, or long-chain fatty acids.
**Results:** We detected ACE2 at the apical and basal poles of hepatocyte chords, in CLEC4M$^+$ liver sinusoidal endothelial cells, the lumen of ABCC2$^+$ bile canaliculi, HepPar-1$^+$-TMPRSS2$^+$ hepatocytes, cholangiocytes, and CD34$^+$ capillary vessels. ACE2 steeply increased between 30 and 50 years of age; was related to liver fat area, inflammation, high immune reactivity, and fibrogenesis; and was upregulated in steatohepatitis. Although *ACE2* mRNA was unmodified in alcoholic or viral hepatitis, it was upregulated in fibroinflammatory livers from overweight patients. *In vitro*, treatment of primary human hepatocytes with inflammatory cytokines alone downregulated but long chain fatty acids upregulated *ACE2* mRNA expression.
**Conclusions:** Lipid overload in fatty liver disease leads to an increased availability of ACE2 receptors.
**Impact and implications:** COVID-19 can be a deadly disease in vulnerable individuals. Patients with fatty liver disease are at a higher risk of experiencing severe COVID-19 and liver injury. Recent studies have indicated that one of the reasons for this vulnerability is the presence of a key cell surface protein called ACE2, which serves as the main SARS-CoV-2 virus receptor. We describe the cellular sources of ACE2 in the liver. In patients with fatty liver disease, ACE2 levels increase with age, liver fat content, fibroinflammatory changes, enhanced positive immune checkpoint levels, and innate immune reactivity. Moreover, we show that long chain fatty acids can induce ACE2 expression in primary human hepatocytes. Understanding the cellular sources of ACE2 in the liver and the factors that influence its availability is crucial. This knowledge will guide further research and help protect potentially vulnerable patients through timely vaccination boosters, dietary adjustments, and improved hygiene practices.
© 2023 The Author(s). Published by Elsevier B.V. on behalf of European Association for the Study of the Liver (EASL). This is an open access article under the CC BY license (http://creativecommons.org/licenses/by/4.0/).

## Introduction

Steatotic liver disease affects 25% of the population, with a prevalence of 1 billion people worldwide. Western diet and lifestyle, and genetic and environmental background lead to common pathological changes, namely, steatosis, inflammation (steatohepatitis), fibrosis, and pre-neoplastic foci.[1] The diagnoses of non-alcoholic fatty liver disease (NAFLD) and non-alcoholic steatohepatitis (NASH) exclude alcohol as an aetiological agent. However, in everyday clinical practice, steatotic liver disease may coexist with viral hepatitis, autoimmune diseases, and alcohol. Thus, the term *metabolic associated fatty liver disease* was proposed in 2020.[1,2] However, a consensus among experts from 56 countries has been reached on the nomenclature for fatty liver disease. The vast majority agreed on the terms metabolic dysfunction-associated steatotic liver disease (MASLD) and metabolic dysfunction-associated steatohepatitis (MASH). These terms were

Keywords: Metabolism; DC-SIGNR; CLEC4M; TMPRSS2; DPP4; SARS-CoV-2; MAFLD; NAFLD; NASH; Metabolic syndrome; Oleic acid; Stearic acid.
*Received 24 February 2021; received in revised form 8 September 2023; accepted 15 September 2023; available online 13 October 2023*
† Equal contributions.
* Corresponding author. Address: INSERM, INRAE, Univ Rennes 1, Nutrition Metabolisms and Cancer, Rennes, France. Tel.: +33-(0)2-23-23-45-65. Fax: +33-(0)2-99-54-01-37.
*E-mail address:* orlando.musso@inserm.fr (O. Musso).

ELSEVIER

EASL The Home of Hepatology

chosen because they are considered less stigmatising and are expected to improve the identification of patients with steatotic liver conditions. MASLD diagnosis is based on steatosis plus at least one of five cardiometabolic criteria, including BMI, insulin resistance, blood pressure, plasma triglycerides, and HDL-cholesterol levels.[3] MASLD severity is assessed in a continuum across a grading of inflammatory activity and the stage of fibrosis.[1–3]

Recent studies showed that patients with metabolic syndrome, MASLD, or MASH are at higher risk of liver injury when infected with the severe acute respiratory syndrome coronavirus 2 (SARS-CoV-2), which causes coronavirus disease 2019 (COVID-19).[4,5] SARS-CoV-2 is an enveloped (60- to 120-nm particle) RNA betacoronavirus phylogenetically similar to SARS-CoV and the Middle East respiratory syndrome coronavirus (MERS-CoV). Approximately 50% of hospitalised SARS-CoV-2-infected individuals present comorbidities, including hypertension, metabolic syndrome, and coronary heart disease. Patients developing severe disease present acute respiratory distress syndrome, immune dysregulation, and a ‘cytokine storm’ with disseminated intravascular coagulation and blood vessel lesions.[6]

Cell entry of SARS-CoV-2 depends on the binding of the viral spike protein to its specific receptor, namely, the angiotensin-converting enzyme 2 (ACE2), and to co-receptors, such as the serine proteases transmembrane serine protease 2 (TMPRSS2) and FURIN for spike protein fusion[7,8] and the C-type lectin domain family 4 member M (CLEC4M) for cell surface adhesion.[9] In patients dying from COVID-19, viral particles have been detected in hepatocytes,[10] and viral RNA has been detected in portal vein endothelial cells.[11] Moreover, HuH7 human hepatocellular carcinoma and HepG2 hepatoblastoma cells express ACE2 and can be infected with SARS-CoV-2 at high titres,[7] and ACE2 downregulation reduces virus infection in human livers perfused *ex situ*.[12] However, both the cellular localisation and expression patterns of the SARS-CoV-2 cell receptor ACE2 and co-receptors remain unclear, in particular in MASLD.

ACE2 is a key player of the renin–angiotensin (Ang)–aldosterone system. Renin, which is secreted by the juxtaglomerular cells of the afferent kidney arterioles, cleaves liver-derived angiotensinogen into angiotensin 1 (Ang I), which is hydrolysed into Ang II by ACE1 and exerts potent vasopressor effects. The carboxypeptidase ACE2 cleaves Ang II into $Ang_{1-7}$, which activates the MAS1 proto-oncogene, G protein-coupled receptor (MAS1), leading to smooth muscle relaxation, hypotension, and cardioprotection. ACE2 cleaves other substrates including the inflammatory peptide des-$Arg_9$-bradykinin and apelin-13; the latter is involved in glucose uptake and energy metabolism.[13] Activation of the ACE2/$Ang_{1-7}$/MAS axis downregulates hepatic lipid uptake and lipogenesis, favouring lipid oxidation, and mitochondrial function, improving glucose metabolism[14,15] and reducing oxidative stress, inflammation, and liver fibrosis in murine models of NAFLD.[16]

Liver sinusoidal endothelial cells (LSECs) have the highest endocytosis capacity of human cells. Together with Kupffer cells, LSECs remove large amounts of antigens, mediate strong inflammatory responses, and recruit leucocytes, which amplify local inflammation.[17] LSECs are CD34 negative and CLEC4M positive (also known as L-SIGN, DC-SIGNR), whereas endothelial cells form CD34-positive capillaries. LSECs capture viruses via lectins at their surface and, in turn, can transfer them to hepatocytes.[18] CLEC4M recognises a wide range of pathogens, among which are SARS-CoV-2, HCV, and Ebola virus.[9] In SARS-CoV, CLEC4M recognises N-glycans on the spike protein and transfers the virus to permissive cells.[19]

We searched for the expression and localisation of ACE2, TMPRSS2, and CLEC4M in normal human liver and MASLD. To this end, we constructed a transcriptomic fatty liver disease meta-dataset of 243 samples including normal liver, steatosis, steatohepatitis, and MASH. To assess whether the results were specific to MASLD or related to unspecific fibroinflammatory liver changes, we explored five additional transcriptomic datasets totalling 161 patients with fibroinflammatory liver diseases. Receptor and co-receptor localisation and cellular sources were studied by high-resolution confocal microscopy scanning after multiplex immunohistochemistry in seven histologically normal liver controls and nine fatty liver disease cases. Cellular sources were also explored by meta-analysis of single-cell RNA sequencing data from two human studies[20,21] totalling 14 normal and five cirrhotic livers and bulk transcriptomics from 745 primary cell samples.[22] Regulation of ACE2 expression was studied on primary human hepatocyte cultures treated with inflammatory cytokines, bacterial lipopolysaccharides (LPS), and long-chain fatty acids.

We show that steatohepatitis was consistently associated with increased expression of ACE2 in LSECs and hepatocytes. In 243 patients with fatty liver disease, *ACE2* mRNA expression progressively increased with age but plateaued between 50 and 80 years and correlated with liver fat area, inflammation, enhanced immune reactivity, and fibrogenesis. In 41 patients with fibroinflammatory liver diseases, *ACE2* expression was upregulated in a background of patient overweight. These findings were supported by upregulation of *ACE2* expression by long-chain fatty acids in an *in vitro* model of steatohepatitis in primary human hepatocytes. By contrast, inflammatory cytokines alone were unable to upregulate *ACE2* expression. Altogether, these data suggest that ACE2 upregulation is a physiological compensatory mechanism in response to a liver overload in fatty acids. However, the co-occurrence of high immune reactivity and increased availability of ACE2 receptors in fatty liver disease may promote viral infection, amplified inflammation, and patient decompensation.

## Materials and methods

### Patients, samples, and datasets

For fatty liver disease samples, routine formalin-fixed, paraffin-embedded non-tumour liver tissues were obtained from the Anatomic Pathology Laboratory, Rennes University Hospital, after informed consent of nine patients with MASLD and five control patients undergoing partial hepatectomy (Table S1). Sample collection, exploitation, and data analysis were performed within the framework of the INSERM’s Institutional Review Board Approval No. 19-630. Routine cases were reviewed and selected by an experienced liver pathologist (BT). Digital slides were viewed using NDP.view (Hamamatsu Photonics). The signal was independently read by two observers (LC and OM), who were blinded to any information. Discrepancies were resolved by consensus reading. Scoring of MASLD and MASH activities was done as described on a four-point scale.[23,24]

A human fatty liver disease transcriptomic meta-dataset of 243 samples was constructed by merging three microarray datasets: GSE33814[25] (12 normal livers, 19 steatoses, and 12 steatohepatitides), GSE48452[26] (14 normal livers, 27 livers from

obese patients, 14 steatoses, and 18 non-alcoholic steatohepatitides [MASHs]), and GSE83452[27] (231 samples from which 126 MASHs were extracted for study). Altogether, the meta-dataset includes 27 normal livers, 27 livers from obese patients, 33 steatoses, 12 steatohepatitides, and 144 MASHs. Batch effect was removed using the ComBat algorithm (*sva* R package), as we previously described.[28] Raw expression data were quantile-normalised and $log_2$-transformed.

### Antibodies and oligonucleotides

Primary and secondary antibodies, antigen retrieval procedures, stainer kits, signal amplification methods, incubation conditions, and dilutions are summarised in Table S2. Real-time PCR primers are detailed in Table S3.

See Supplementary information for a detailed description of standard benchwork procedures such as human primary hepatocyte isolation and culture, immunohistochemistry, cell culture and treatments, RNA extraction and real-time PCR, deconvolution of immune cell populations, and statistical analyses.

## Results

### High expression of ACE2 protein in liver sinusoids in steatohepatitis

We previously showed that ACE2 is an attribute of normal adult hepatocyte identity.[29] Recent studies consistently showed that human hepatocytes[10,12,15,30] and cholangiocytes[12] can be infected with SARS-CoV-2. Of note, when human cholangiocyte organoids lose their identity *in vitro,* they no longer express ACE2.[12] In fatty liver disease, the mRNA expression of *ACE2* and that of the SARS-CoV-2 co-receptor *TMPRSS2* are upregulated.[31,32] However, their precise cellular sources and the factors that trigger their upregulation in the clinical setting remain elusive. By immunohistochemistry, the primary antibodies and protocols used (Table S2) confirmed the expected high levels of ACE2 in the human kidney (Fig. S1A), the intestine (Fig. S1B), and cholangiocytes (Fig. 1A–D) and of TMPRSS2 in the human prostate (Fig. S1C and D). Of note, our analysis of a transcriptomic microarray meta-dataset assembling 745 primary cell samples from more than 100 studies[22] revealed that *ACE2* and *TMPRSS2* mRNAs were detected in hepatocytes and the bronchial epithelium at similar levels (Fig. S2A). These findings were consistent with the reported detection of ACE2 protein in human livers[33] and the detection of low but homogenously distributed ACE2 enzymatic activity by *in situ* autoradiography in normal rat livers.[34]

We analysed ACE2 and TMPRSS2 expression and localisation in nine cases of fatty liver disease and five controls by immunohistochemistry. Available clinical and biological data, as well as scores for steatosis, ballooning, inflammation, MASLD, and MASH are shown in Table S1. In normal livers, we did not detect TMPRSS2 protein (not shown) but confirmed ACE2 in bile ducts, capillary blood vessels within portal tracts, and sinusoidal cells (Fig. S2B and C). In steatohepatitis, we detected ACE2 (Fig. 1A and B) in hepatocytes within parenchymal nodules outlined by inflammatory infiltrates. ACE2 was also detected in sinusoidal cells (Fig. 1C and D) and in capillary blood vessels within inflamed portal tracts (Fig. 1E). Image analysis revealed higher levels of ACE2 in nine cases of fatty liver disease *vs.* five controls (Fig. 1F). Although TMPRSS2 protein was detected at lower levels than

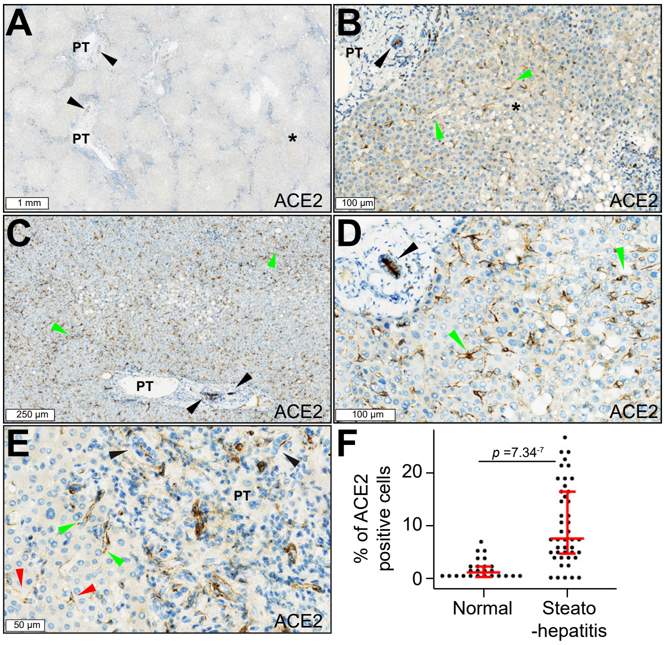


**Fig. 1. Increased expression of ACE2 in steatohepatitis.** (A) ACE2-positive bile ducts (brown signal, arrowheads), enlarged PTs with inflammatory infiltrates, and incomplete fibrous septa outlining parenchymal nodules with variable levels of ACE2 staining (asterisk). (B) Steatohepatitis with predominantly ACE2-positive hepatocytes: ACE2-positive bile ducts (black arrowhead), macrovesicular steatosis with predominantly ACE2-positive hepatocytes (asterisk), and sparsely ACE2-positive sinusoidal cells (green arrowheads). (C and D) Macrovesicular steatosis with predominantly ACE2-positive staining in sinusoidal cells (green arrowheads) and bile ducts (black arrowheads). (E) PT with inflammatory infiltration and ductular reaction (black arrowheads). ACE2-positive cord-like structures resembling blood vessels (green arrowheads) are seen at the interface between the limiting plate and the parenchyma. Sinusoids are lined by ACE2-positive cells (red arrowheads). Digital slides were acquired in a microscope scanner using a 40× objective. (F) Percentage of ACE2-positive cells in normal liver controls (n = 5) and steatohepatitis (n = 9). From each patient, five ACE2-positive 1-$mm^2$ hotspots were selected for image analysis. Each dot represents one hotspot; whisker bars show median plus first and third quartiles. The Mann–Whitney *U* test was used to assess statistical significance. Clinical data, and MASLD and MASH scores are shown in Table S1. ACE2, angiotensin-converting enzyme 2; PT, portal tract.

was ACE2, both were co-expressed in hepatocytes in steatohepatitis samples (Fig. 2D–G).

### Detection of ACE2 in sinusoidal endothelial cells and bile canaliculi in steatohepatitis

Immunohistochemistry confirmed colocalisation of ACE2 and the LSEC marker CLEC4M (also known as DC-SIGNR) in normal liver and steatohepatitis (Fig. 2A–I). Colocalisation with CD34-confirmed ACE2 in capillary endothelial cells within inflamed portal tracts (Fig. 3A–C). Moreover, ACE2 colocalised with the bile canaliculi marker ATP binding cassette subfamily C member 2 (ABCC2, also known as multidrug resistance-associated protein 2 [MRP2]) (Fig. 3D–F), which is consistent with the abundance of ACE2 in the human bile proteome.[35] Altogether, these findings showed that the basal and apical poles of hepatocyte chords display ACE2 receptors in patients with steatohepatitis.

We also searched for ACE2 protein expression in resident liver immune cells. ACE2 was not detected at significant levels

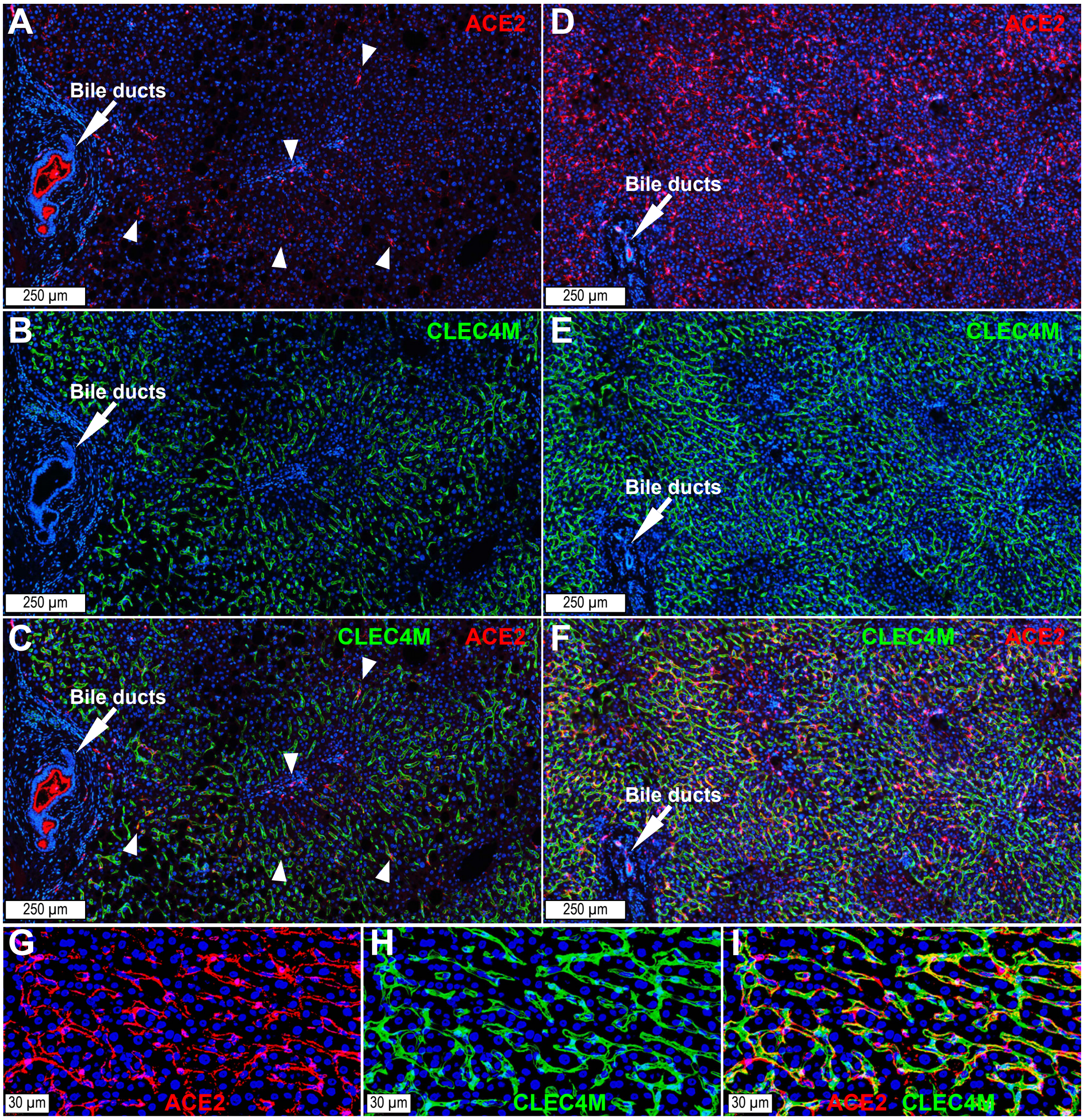


**Fig. 2. ACE2 in sinusoidal endothelial cells in (A–C) normal liver and (D–F) steatohepatitis.** ACE2-positive signal (red) colocalises with the liver sinusoidal **endothelial cell marker CLEC4M (also known as DC-SIGNR) (green) lining the** sinusoidal endothelium. In normal liver, sparse ACE2-positive sinusoidal cell spots are seen at low power (white arrowheads). Bile ducts show strong ACE2 signal (white arrows). In steatohepatitis, a high density of ACE2-positive sinusoidal cells is observed. (G–I) Higher power view of ACE2 expression in the sinusoidal endothelium in steatohepatitis. Nuclei are seen in blue (DAPI). Digital slides were acquired using a 40× objective in a confocal scanner. The images show a Z-stack of four 500-nm focusing steps. ACE2, angiotensin-converting enzyme 2; CLEC4M, C-type lectin domain family 4 member M.

in $CD45^+$ leucocytes (Fig. S3). Frequent images of $CD68^+$ Kupffer cell membrane extensions in close contact with $ACE2^+$ endothelial cells and thus overlapping within the same focal plane were seen (Fig. S4A and D). In this case, digital deconvolution of high-power confocal images across 500-nm steps in the Z axis showed that $CD68^+$ Kupffer cell membrane extensions lined $ACE2^+$ sinusoidal endothelial cells (Fig. S4E and G). ACE2 was not detected at significant levels in $CD3^+$ T lymphocytes or in Actin alpha 2, smooth muscle (ACTA2)-positive myofibroblasts (Fig. S5).

Meta-analysis of single-cell sequencing data of histologically normal liver samples from nine patients[20] confirmed that *ACE2* mRNA levels are globally low in all cell populations tested. Scattered *ACE2*-positive cells were detected in clusters containing sinusoidal endothelial cells, periportal and perivenous hepatocytes, and cholangiocytes (Fig. S6). In addition, single-cell RNA sequencing data of five normal and five cirrhotic human livers (including NAFLD, alcohol-related liver disease, and primary biliary cirrhosis)[21] revealed low levels of *ACE2* and *TMPRSS2* in hepatocytes, cholangiocytes, and mesenchymal and endothelial cells (Fig. S7A–C). Taken together, our experimental and meta-analyses data showed low general levels of *ACE2* and *TMPRSS2* in all liver cell subsets tested, with upregulation of *ACE2* mRNA[31,32] and protein in fatty liver disease. ACE2 was located

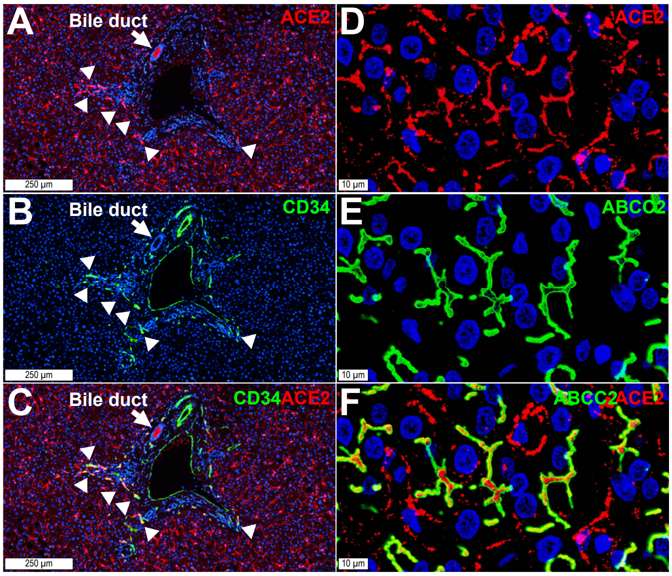


**Fig. 3. ACE2 in (A–C) CD34-positive capillary endothelial cells and (D–F) bile canaliculi.** (A–C) Steatohepatitis. CD34-positive endothelial cells (green, arrowheads) within portal tracts and at the interface between the limiting plate and the parenchyma are ACE2-positive (red, arrowheads). (D–F) Bile canaliculi, identified with the specific marker ABCC2 (also known as MRP2) (green), contain ACE2 (red). Digital slides were acquired using a 40× objective in a confocal scanner. The image shows a Z-stack of four 500-nm focusing steps. ABCC2, ATP binding cassette subfamily C member 2; ACE2, angiotensin-converting enzyme 2; MRP2, multidrug resistance-associated protein 2.

mainly at the sinusoidal and biliary poles of hepatocyte cords. In summary, cholangiocytes, sinusoidal endothelial cells, and hepatocytes are the major cell sources of ACE2 protein in the liver. Resident liver immune cells do not appear to express detectable levels of ACE2 protein in steatohepatitis, as observed using the highly sensitive tyramide signal amplification method.

### Liver *ACE2* mRNA expression increases proportionally with age, inflammation, and fibrogenesis in patients with fatty liver disease

We assessed the regulation of *ACE2* and *TMPRSS2* expression in MASLD after merging three independent microarray datasets[25–27] into a meta-dataset consisting of 243 liver samples and 20,941 mRNA transcripts. Batch effect correction and normalisation were applied to minimise experimental bias (Fig. S8A). The expression of *TMPRSS2* remained unchanged in MASLD, whereas the MERS-CoV receptor dipeptidyl peptidase 4 (*DPP4*) and the SARS-CoV-2 coreceptor CLEC4M were lower in steatosis and steatohepatitis, with respect to controls (Fig. S8B). By contrast, *ACE2* mRNA reached higher levels in MASH and steatohepatitis (Fig. 4A). An expression profile similar to that of *ACE2* was detected for the extracellular matrix components *COL1A1, COL3A1, VCAN, COL4A1, and LAMC1* (Fig. 4A), which suggests that *ACE2* expression is upregulated concomitantly with fibrogenesis. *ACE2* expression was also correlated with patient age, fat area, and inflammation score in liver biopsies (Fig. 4B). Remarkably, the ACE2/age relationship drew a steep slope between 30 and 50 years of age and plateaued afterwards. Fat area was also related to the expression of the major extracellular matrix components *COL1A1, COL3A1*, and *VCAN* (Fig. 4C). In turn, the mRNA level of these extracellular matrix components was directly proportional to the inflammation scores (Fig. 4D). Altogether, these data reveal upregulation of *ACE2* mRNA expression in MASLD.

### The expression of *ACE2*, *TMPRSS2*, and *DPP4* mRNAs in fatty liver tissues correlates with markers of immune cell infiltration

The absence of detectable ACE2 protein in liver immune cells by immunohistochemistry, despite the use of the highly sensitive tyramide-based signal amplification technology, led us to search for mRNA expression of *ACE2*, *TMPRSS2*, and *DPP4* in immune cell subsets isolated from circulating leucocytes. To this end, we analysed the *Immune Response in Silico* (IRiS) microarray dataset, composed of 228 immune cell samples that had been isolated by cell-specific antibody pull-down before transcriptomic analyses.[36] First, we confirmed detection of immune cell subset marker mRNAs specific to the cell types analysed (Fig. S9). Next, we detected *ACE2* in B lymphocytes (naive B cells, and IgA and IgM/G memory and plasma cells) and neutrophils. In turn, *TMPRSS2* was detected at variable levels in all cell types except in plasma cells. By contrast, DPP4 was restricted to $CD4^+$ T, $CD8^+$ T, and natural killer lymphocytes (Fig. S10A). Of note, although *ACE2* and *TMPRSS2* mRNAs were detected in purified immune cells from circulating leucocytes, the mRNA abundance was 100-fold lower than that of cell surface cluster differentiation immune cell markers (compare Figs. S9 and S10A).

To infer whether expression of *ACE2, TMPRSS2,* and *DPP4* mRNAs in fatty liver disease tissues was associated with inflammatory infiltration, we explored the GSE33814 dataset composed of 13 normal livers, 19 steatoses, and 12 steatohepatitides[25] and searched for correlations with immune cell subset-specific markers, according to the IRiS immune cell repository[36] (Fig. S10B). Liver *ACE2* mRNA expression was most highly correlated with liver infiltration by T and B lymphocytes, macrophages, and dendritic cells. *TMPRSS2* mRNA was associated with macrophage and neutrophil markers, whereas *DPP4* was associated with macrophage, neutrophil, and T-cell markers. Validating the above findings, we found correlation of *ACE2* with T-cell, B-cell, and dendritic cell markers; *TMPRSS2* with macrophage and neutrophil markers; and *DPP4* with T-cell markers in an independent dataset (GSE48452; Fig. S11).[26]

These results show that upregulation of *ACE, TMPRSS2*, and *DPP4* mRNA expression correlates with immune cell infiltration in steatohepatitis. They match with the higher levels of ACE2 protein expression seen in steatohepatitis with respect to control livers and with the association of *ACE2* mRNA levels with fibroinflammation in fatty liver disease. Taken together, these results show an association between the upregulation of the expression of SARS-CoV-2 entry points in the liver and inflammation. Although the virus could use circulating immune cells as shuttles to colonise the liver, resident liver immune cells do not exhibit significant levels of the major SARS-CoV-2 receptor ACE2.

### Steatohepatitis shows enhanced immune reactivity

To appraise the functional significance of immune cell infiltrates in MASLD, we constructed immunophenograms *(i.e.* phenetic diagrams) that classify immune functions into four families, namely, major histocompatibility complex, effector cells, suppressor cells, and checkpoint inhibitors and immune modulators (Fig. 5A), applying deconvolution algorithms.[37] Immunophenograms were obtained from 13 controls, 19 steatoses, and 12 steatohepatitides (Fig. S12) from the GSE33814 transcriptomic

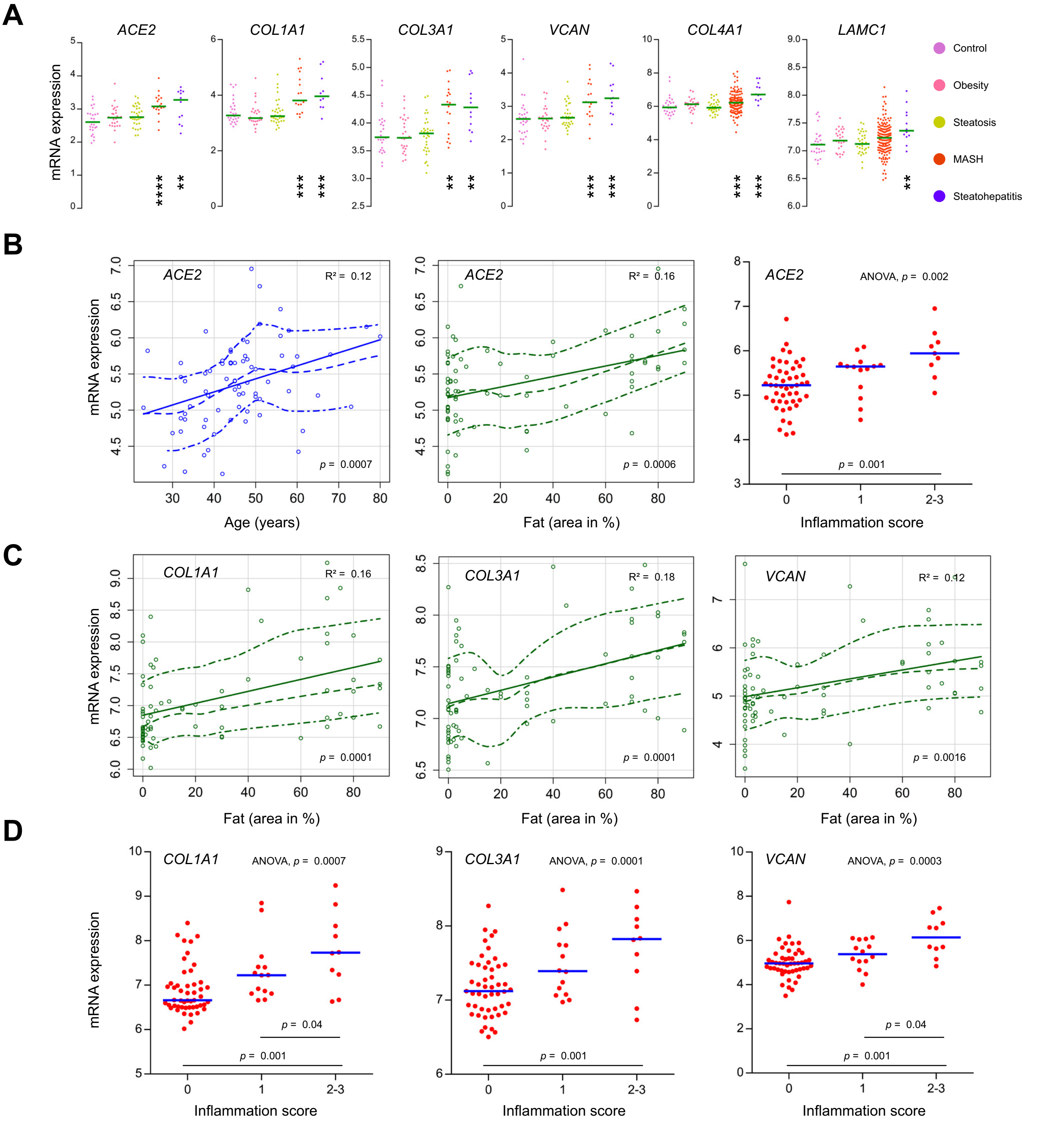


**Fig. 4.** ***ACE2* mRNA expression increases proportionally with age, fat content, inflammation, and fibrogenesis in 243 patients with fatty liver disease.** Meta-dataset constructed from three independent datasets (GSE33814, GSE48452, and GSE83452). After quantile normalisation and batch effect suppression, it was composed 243 liver samples (27 normal controls, 27 obese, 33 steatoses, 144 MASHs, and 12 steatohepatitides) and 20,941 different RNA transcripts. (A) *ACE2* is associated with the fibrogenesis markers *COL1A1*, *COL3A1*, and *VCAN,* and basement membrane remodelling markers *COL4A1* and *LAMC1.* ANOVA followed by Tukey's *post hoc* test was used (***p* <0.01, ****p* <0.001, *****p* <0.0001; asterisks compare pathological conditions with controls). For each gene, the numbers of available observations are indicated in Table S4. (B) *ACE2* mRNA expression is associated with increasing age, liver biopsy fat area, and inflammation score in 72 patients from the GSE48452 dataset. (C and D) The fibrogenesis markers *COL1A1*, *COL3A1*, and *VCAN* are associated with increasing liver fat area (C) and inflammation (D). ACE2, angiotensin-converting enzyme 2; MASH, metabolic dysfunction-associated steatohepatitis.

dataset.[25] Data are summarised in a spider web chart (Fig. 5A). We observed enhanced immune reactivity in steatohepatitis, with increase in major histocompatibility complex markers involved in antigen processing and presentation (*HLA-F*, *HLA-DPA1*, *HLA-B*, *HLA-A*, and *TAP2*) and positive immunomodulation (*CD27* and *ICOS*), with downregulation of the negative

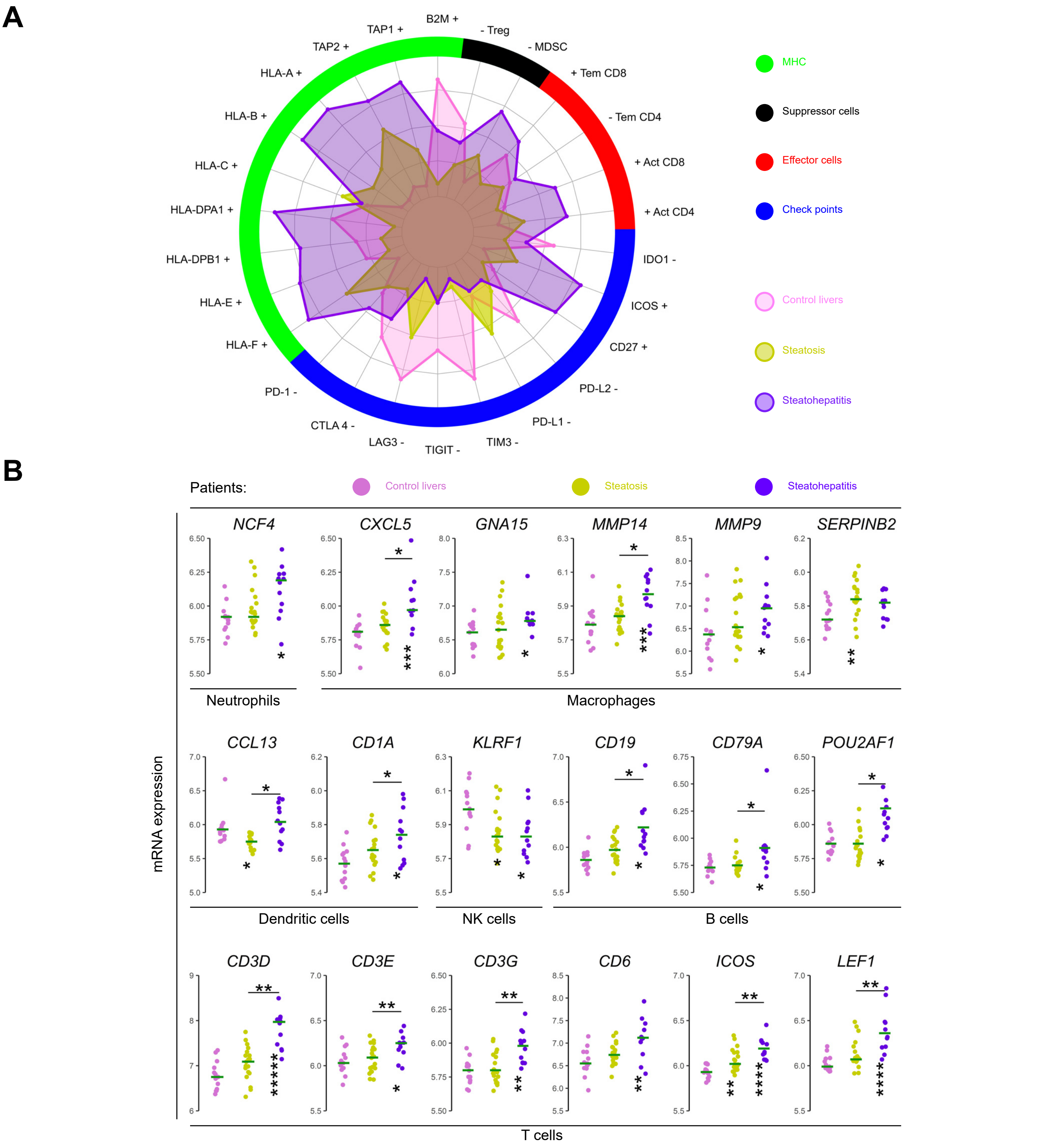


**Fig. 5. Enhanced immune reactivity in steatohepatitis: antigen presentation, CD4 T-cell activation, and checkpoint modulation.** (A) Immune cell subsets in 44 human liver samples from the GSE33814 dataset (13 controls, 19 steatoses, and 12 steatohepatitides) are classified into four immunogenicity functional families: MHC, effector cells, suppressor cells, and checkpoints. Enhancement and suppression of immune reactivity are indicated by$^+$ and $^-$, respectively. Parameters' full names and gene symbols are indicated in Table S5. Steatohepatitis shows enhancement of MHC (*HLA-F*, *HLA-DPA1*, *HLA-B*, *HLA-A*, and *TAP2*) and two positive immunomodulators (*CD27* and *ICOS*), with moderate activation of CD4 T cells and downregulation of the checkpoint inhibitor *TIGIT*. Statistical significances are shown in Table S6. (B) Fatty liver disease samples from the GSE33814 dataset show increased expression of molecular markers for neutrophils, macrophages, dendritic cells, NK cells, and B and T cells. Decreased expression of *KLRF1* indicates NK cell activation.[36] ANOVA was followed by Tukey's *post hoc* test to assess the statistical significance of the difference between *control* and *steatosis* or *steatohepatitis* (*$p$ <0.05, **$p$ <0.01, ***$p$ <0.001, ****$p$ <0.0001). MHC, major histocompatibility complex; NK, natural killer.

checkpoint *TIGIT* and activation of CD4 T cells. We confirmed these data using two different approaches. First, using the IRiS tool,[36] we found that steatohepatitides were enriched in neutrophils, macrophages, dendritic cells, natural killer lymphocytes, B lymphocytes, and T lymphocytes (Fig. 5B). Second, we constructed an unsupervised gene co-expression network to identify densely connected sub-networks or modules relating biological functions with the patient groups. Applying weighted gene co-expression network analysis to the meta-dataset consisting of 243 human liver samples, we identified three modules highly associated with steatohepatitis (Figs. S13–16 and Table S7). Gene ontology analysis confirmed functions involved in antigen processing and presentation, lymphocyte chemotaxis, inflammation, fibrogenesis, and typical hepatocyte metabolism. These findings confirm an enhanced immune reactivity in steatohepatitis leading to a chronic fibroinflammatory microenvironment.

### *ACE2* expression is upregulated in chronic fibroinflammatory liver diseases in a context of patient overweight

We tested whether the upregulation of ACE2 was specific to steatohepatitis or whether this was also seen in other fibroinflammatory liver diseases. Analysis of four transcriptomic datasets totalling 120 patients did not reveal significant *ACE2* mRNA changes in the liver in alcoholic hepatitis (GSE28619), blood mononuclear cells (GSE119117), and the liver in HCV infection (GSE48445) or in the liver in HBV infection (GSE54747), except that treatment of patients with HCV with pegylated interferon alpha 2 (IFNA2) was associated with decreased *ACE2* expression with respect to untreated patients (Fig. S17).

We then asked whether recombinant cytokines could upregulate *ACE2* mRNA expression in hepatocytes. Isolation of primary normal human hepatocytes from three patients followed by *in vitro* treatment with IL-6, IL-1B, TNFA, or LPS decreased *ACE2* mRNA expression by several folds, without significant changes in *TMPRSS2* expression. In turn, IL-1B, TNFA and LPS decreased, but IL6 increased *FURIN* mRNA levels. (Fig. S18). These findings are consistent with downregulation of or no effect on *ACE2* mRNA expression of IL-1B, IL-6, IL-10, IL-18, or IFNA2 in combination with IL-1B, IL-6, and TNFA.[38] They also agree with the notion that inflammatory cytokines lead to a loss of the differentiated hepatocyte identity.[39] Of note, as we previously showed, *ACE2* is preferentially expressed in differentiated hepatocytes.[29]

Treatment of human microvascular endothelial cells with IL-6, IL-1B, TNFA, or LPS did not significantly change *ACE2*, *TMPRSS2*, or *FURIN* mRNA expression (Fig. S19). Thus, we hypothesised that ACE2 upregulation could result from the synergy between chronic inflammation and fibrogenesis in a context of patient overweight. We analysed the mRNA expression of *ACE2* and the markers of fibrogenesis *COL1A1, COL3A1,* and *VCAN* in 41 non-tumour livers from TCGA dataset according to the BMI and the staging of inflammatory activity and fibrosis, as assessed by METAVIR scoring[40] on digital slides. Table S8 shows METAVIR scores, as well as anonymised IDs; BMI; *ACE2*, *COL1A1*, *COL3A1*, and *VCAN* mRNA levels; and aetiologies. METAVIR inflammation scores were dichotomised as *low* (0–1) and *high* (2–3). METAVIR fibrosis scores were dichotomised as *low* (0–1–2) and *high* (3–4). BMI was available for 30/41 patients, 60% of whom (18/30) had a BMI >23 (overweight). High mRNA expression of *ACE2*, *COL1A1*, *COL3A1*, and *VCAN* correlated with the histological grading of inflammation and fibrosis (Fig. 6A). Discriminant function analysis showed the percent of variance in *ACE2* mRNA levels explained by fibrosis (*METAVIR F*), inflammation (*METAVIR A*), and BMI in 41 patients (Fig. 6B). Inclusion of BMI increased the discriminatory ability of the model. The fact that *ACE2* mRNA levels correlated with the markers of hepatocyte metabolism *OTC* and *GLS2* in 30 patients with steatohepatitis confirms our previous findings of *ACE2* expression in metabolically competent hepatocytes.[29] Taken together, these findings suggested that ACE2 expression was upregulated in chronic fibroinflammatory liver diseases in a context of patient overweight.

### Oleic and stearic acids induce steatosis in normal human hepatocytes and lead to increased *ACE2* mRNA expression *in vitro*

The above results led us to compare the impact of two statistical models of the variance of the MASLD meta-dataset by combining *ACE2* mRNA expression with either inflammatory cytokines or markers of fatty liver disease. Both models had similar impact on Wilk's Lambda scores and confirmed the differences in *ACE2* expression between 27 control patients and 30 patients with steatohepatitis (Fig. 7A and B). These results are in line with a hypothetical interaction between fatty liver disease and chronic inflammation on ACE2 upregulation. To experimentally test this hypothesis, we challenged primary cultures of normal human hepatocytes from three patients by *in vitro* modelling the metabolic effects of steatohepatitis (Fig. 7C).[41] Treatment of hepatocytes with the long-chain fatty acids oleic acid or oleic + stearic acids led to steatosis as shown by Bodipy staining (Fig. 7D) and increased expression of the steatosis marker perilipin 2 (*PLIN2*) mRNA (Fig. 7E). Under these conditions, *ACE2* expression was upregulated, unlike *TMPRSS2* and *FURIN*.

In summary, we conducted a multiparametric study of ACE2 expression and cellular sources in fatty liver diseases. We analysed liver bulk transcriptomes from eight independent sets totalling 404 patients with chronic fibroinflammatory liver diseases of different aetiologies. ACE2 protein was detected *in situ* at the sinusoidal and biliary poles of hepatocyte cords in nine patients with steatohepatitis and five controls. Single-cell liver RNA sequencing meta-analyses were done in 19 patients including those with NAFLD and alcohol-related liver disease. Finally, we *in vitro* modelled steatohepatitis in primary human hepatocyte cultures. In conclusion, converging evidence concurs to indicate that fibroinflammatory liver disease in a background of fatty liver leads to increased expression of the SARS-CoV-2 receptor ACE2.

## Discussion

We show that the expression of ACE2 is upregulated in chronic fibroinflammatory liver diseases in a context of patient overweight. In particular, the basal and apical poles of hepatocyte chords display an increased density of ACE2 receptors in patients with steatohepatitis. High liver ACE2 expression is related to the percentage of fat area, enhanced liver immune reactivity, and fibrogenesis. *In vitro* modelling of steatohepatitis by treating primary normal human hepatocytes with the long-chain fatty acids oleic and stearic acids leads to increased *ACE2* mRNA expression, confirming the molecular data obtained from clinical samples.

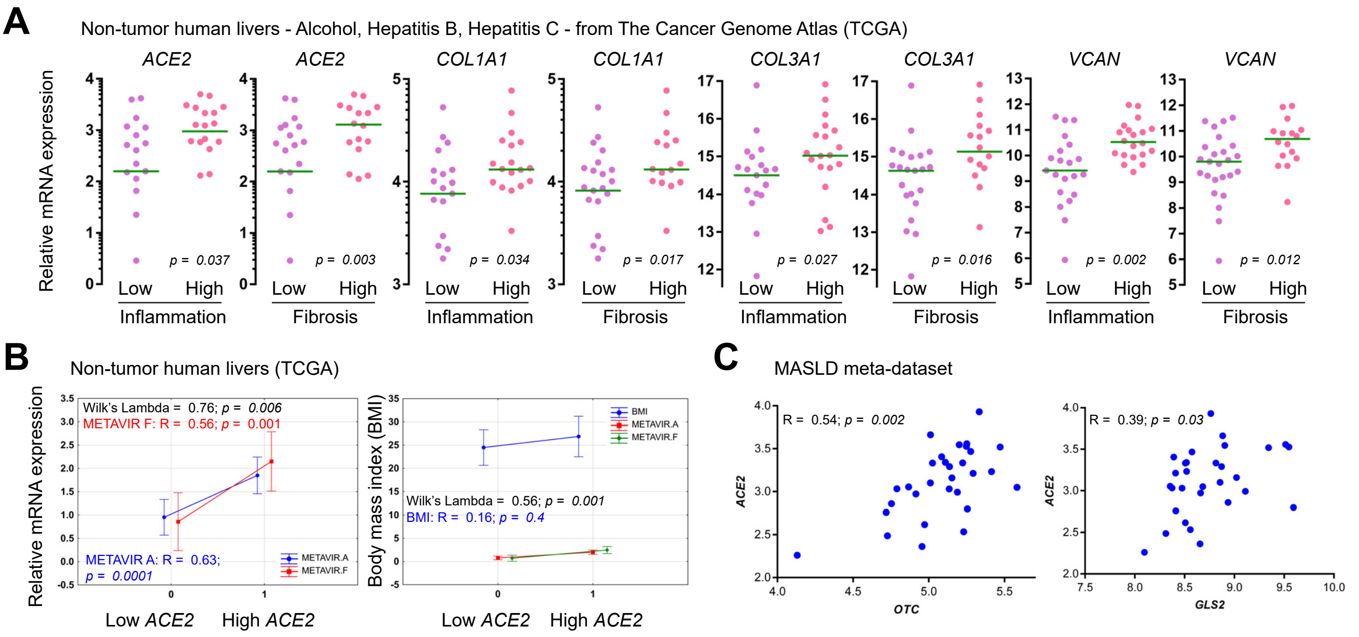


**Fig. 6. *ACE2* mRNA expression is upregulated in chronic fibroinflammatory liver diseases resulting from alcohol abuse or viral hepatitis in a context of patient overweight.** (A) *ACE2* in 41 non-tumour livers from TCGA dataset according to the grading of inflammatory activity and fibrosis, as assessed by METAVIR scoring. Inflammation scores: *low* (0–1) and *high* (2–3). Fibrosis scores: *low* (0–1–2) and *high* (3–4). BMI was available for 30/41 patients; 60% of patients (18/30) had a BMI >23 (overweight). The indicated markers of fibrogenesis correlate with *ACE2* expression. Case scoring is shown in Table S8. Statistical significance of the differences between *low* and *high* were assessed using the Mann–Whitney *U* test. (B) Discriminant function analysis shows the percent of variance in *ACE2* mRNA levels explained by fibrosis (*METAVIR F*), inflammation (*METAVIR A*), and BMI in 41 patients. Smaller values of Wilk's lambda indicate greater discriminatory ability of the function. Inclusion of BMI increases the discriminatory ability of the model. (C) *ACE2* mRNA levels are correlated with the markers of hepatocyte metabolism *OTC* and *GLS2* in 30 patients with steatohepatitis. ACE2, angiotensin-converting enzyme 2; MASLD, metabolic dysfunction-associated steatotic liver disease; TCGA, The Cancer Genome Atlas.

ACE2 was also detected in CLEC4M$^+$ sinusoidal endothelial cells and in CD34$^+$ portal capillaries in steatohepatitis. CLEC4M may facilitate the transfer of viruses from the sinusoidal endothelium to ACE2-positive cells.[19] Although TMPRSS2 protein was not detected in normal liver, it was co-detected with ACE2 in hepatocytes in steatohepatitis. Therefore, ACE2, TMPRSS2, and CLEC4M could support viral infection through the space of Disse. In consistency with these findings, SARS-CoV-2 has been detected within endothelial cells in portal veins[42] and sinusoidal microthrombi[43] and associated with vascular dysfunction, such as occlusive thrombosis, in patients who died from COVID-19.[11,44]

In contrast with our findings, a recent report[45] concluded that there was no evidence for an increased *ACE2* expression in MASLD. The authors found no difference in *ACE2* mRNA expression between MASLD, steatosis, and MASH after analysis of the GSE48452[26] transcriptomic microarray dataset. First, the GSE48452 dataset (n = 54) is included within our meta-dataset consisting of 243 patients with fatty liver disease. Second, we agree that, in this dataset, there is no difference between lean patients without MASLD and the other groups. However, *ACE2* mRNA levels were higher in patients with MASH than in obese patients without MASLD and in patients with simple steatosis (Wilcoxon test adjusted for multiple testing using Benjamini and Hochberg's correction; Fig. S20). In consistency with our results, *ACE2* and *TMPRSS2* upregulation in MASH correlates with activity score.[32] Moreover, international studies including over 60 million patients revealed a higher risk for COVID-19 in metabolic syndrome. In particular, patients with MASH,[5] MASLD,[46] and NAFLD[4] are at high risk of severe COVID-19 and liver injury.

Our data suggested that a background of fatty liver disease is important for upregulation of ACE2 to occur. Several lines of evidence substantiate this hypothesis. First, patients with alcoholic, HBV, or HCV hepatitis did not present increased *ACE2* mRNA levels. By contrast, in overweight patients, *ACE2* mRNA was correlated with fibroinflammatory activity. Discriminant function analysis showed that the variance in *ACE2* mRNA levels was better explained by a model including BMI than not.

Second, our model of the metabolic effects of steatohepatitis in primary normal human hepatocytes using long-chain fatty acids revealed that steatosis upregulates *ACE2* expression *in vitro*. In consistency with our results, primary human hepatocytes where steatosis had been induced by methionine and choline deprivation are more vulnerable to SARS-CoV-2 infection.[15] However, induction of steatosis in HepG2 hepatoblastoma cells with palmitic acid (PA) has led to contrasting results. PA alone was reported to increase[47] and reduce[48] ACE2 expression. In the latter report, it was the association of PA with the glucagon-like peptide liraglutide that activated the Ang$_{1-7}$/Mas axis downstream ACE2, reducing inflammation in fatty liver disease in mice. Similarly, a murine model of Ang II-dependent hypertension supplemented with omega-3 fatty acids increased *ACE2* and reduced inflammation.[49]

Third, treatment of primary human hepatocytes with recombinant IL-6, IL-1B, TNFA, or LPS decreased *ACE2* mRNA

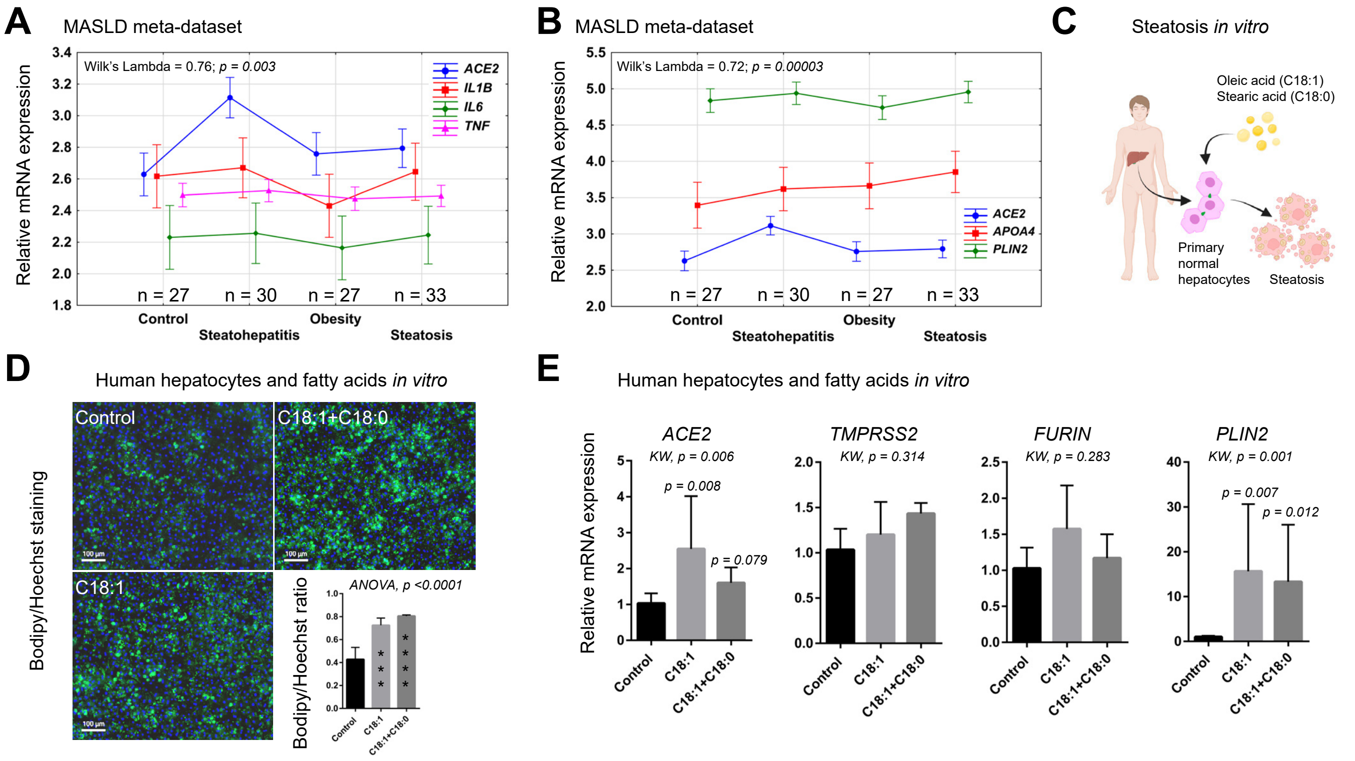


**Fig. 7.** ***ACE2* expression is induced by fatty acids in primary human hepatocytes *in vitro*.** (A) Multiple discriminant analysis showing the proportion of variance in the MASLD meta-dataset explained by *ACE2* and the markers of inflammation *IL-1B, IL-6*, and *TNF*. The curve slopes show that *ACE2* accounts for the highest variance in steatohepatitis. Vertical bars indicate 95% CIs. (B) Multiple discriminant analysis showing the percent of variance in the MASLD meta-dataset explained by *ACE2* and the markers of steatosis *APOA4* and *PLIN2*. Vertical bars indicate 95% confidence intervals. (C) Schematic outline of *in vitro* induction of steatosis with fatty acids in primary human hepatocytes. Oleic acid alone is used to induce steatosis, whereas oleic and stearic acid together are used to model MASH-like metabolic effects.[41] Cartoon constructed using BioRender (https://biorender.com/). (D) Representative images of primary human hepatocytes treated with 150 μM oleic acid (C18:1) or oleic plus stearic acids (C18:1 + C18:0; 150 μM each) for 1 week and labelled with the lipid stain Bodipy 493/503 (green) and the nuclear stain Hoechst (blue). The Bodipy/Hoechst ratio indicates fatty acid accumulation in hepatocytes. Asterisks indicate statistical differences with control (****p* <0.001, *****p* <0.0001). (E) Primary human hepatocytes from three patients were treated with 150 μM oleic acid (C18:1) or oleic plus stearic acids (C18:1 + C18:0; 150 μM each) for 1 week. Hepatocytes were seeded and treated in duplicate wells. RNA from each well was analysed in duplicate by real-time RT-PCR using the $2^{-\Delta\Delta Ct}$ method. Bars show means ± SD from three patients. Statistical intergroup differences were assessed using KW ANOVA as indicated, followed by Dunn's *post hoc* multiple comparison test between control and fatty acid-treated wells. High *PLIN2*, a protein that coats lipid droplets, confirms the presence of steatosis. ACE2, angiotensin-converting enzyme 2; KW, Kruskal–Wallis; MASLD, metabolic dysfunction-associated steatotic liver disease; MASH, metabolic dysfunction-associated steatohepatitis; *PLIN2*, perilipin 2; TCGA, The Cancer Genome Atlas.

expression. Similar results were reported by other authors.[38] Indeed, inflammatory cytokines lead to a loss of hepatocyte identity.[39] Hepatocyte[29] or cholangiocyte[12] dedifferentiation results in loss of ACE2 expression. These findings agree with our observation that *ACE2* mRNA levels correlate with the markers of hepatocyte differentiation *OTC* and *GLS2*.

An alternative mechanism leading to ACE2 upregulation could result from the antiviral IFN response. Type 1 IFNs, specifically IFNA2, whose levels are increased in patients with severe COVID-19, increase ACE2 expression and viral copy number *in vitro*.[38] The same mechanism could explain higher ACE2 expression in primary hepatocytes infected with HCV *in vitro*.[30] However, our meta-analysis of 30 HCV-infected patients from GSE48445 (Fig. S17) revealed that treatment with pegylated IFNA2 resulted in decreased *ACE2* expression. This raises questions about the interactions between HCV viral load and the expression of endogenous IFNA2 and ACE2 across the timeline of antiviral treatment in the era of direct antiviral agents against HCV.

Fourth, by digital sectioning of bile canaliculi using confocal microscopy, we found that the lumen of bile canaliculi in steatohepatitis contained ACE2. Indeed, ACE2 is shed from the cell surface by ADAM17[50] and is thus found abundantly in the human bile proteome.[35] Moreover, high serum levels of ACE2 are found in cholestasis because bile acids control *ACE2* gene expression through farnesoid X receptor (FXR) response elements in its promoter.[12] Fatty acids can also activate FXR, and inflammation and hepatocyte ballooning in steatohepatitis can induce cholestasis. Therefore, FXR and ACE2 could be involved in a regulatory loop in fatty liver disease. In support of this hypothesis, FXR downregulates liver lipogenesis, thus protecting against NAFLD,[51] and ACE2 contributes to postprandial amino acid absorption,[13] thus improving hepatocyte homoeostasis.

Altogether, this body of evidence raises the hypothesis that high ACE2 expression in response to an excess in fatty acids could protect hepatocytes from inflammation and activate fatty acid oxidation through the $Ang_{1-7}$/Mas axis. In turn, SARS-CoV-2 evolution may have exploited this regulatory loop, which

increases susceptibility to infection. The concomitance of enhanced innate immune reactivity with increased availability of ACE2 receptors in a backdrop of fatty liver disease may promote viral infection, endocytosis of virus-receptor complexes, rapid cell surface ACE2 depletion, and finally metabolic decompensation and amplified inflammation.

## Abbreviations

ABCC2, ATP binding cassette subfamily C member 2; ACE2, angiotensin-converting enzyme 2; ACTA2, Actin alpha 2, smooth muscle; Ang, angiotensin; CLEC4M, C-type lectin domain family 4 member M; COVID-19, coronavirus disease 2019; DPP4, dipeptidyl peptidase 4; FXR, farnesoid X receptor; IFNA2, pegylated interferon alpha 2; IRiS, Immune Response in Silico; LPS, lipopolysaccharides; LSEC, liver sinusoidal endothelial cell; MASH, metabolic dysfunction-associated steatohepatitis; MASLD, metabolic dysfunction-associated steatotic liver disease; MERS-CoV, Middle East respiratory syndrome coronavirus; MRP2, multidrug resistance-associated protein 2; NAFLD, non-alcoholic fatty liver disease; PA, palmitic acid; SARS-CoV-2, severe acute respiratory syndrome coronavirus 2; TCGA, The Cancer Genome Atlas; TMPRSS2, transmembrane serine protease 2.

## Financial support

This work was supported by Inserm; Univ Rennes 1; Ministère de l'Enseignement Supérieur; Institut National Du Cancer, grant no. INCA_12688; and Ligue Nationale Contre le Cancer 2018, Comités d'Ille-et-Vilaine et Vendée.

## Conflicts of interest

The authors of this study declare that they do not have any conflict of interest.

Please refer to the accompanying ICMJE disclosure forms for further details.

## Authors' contributions

Study design: OM, LD, LC, AC, BC, JM, CA. Cell culture experiments and molecular biology analyses: GA, ELF, CA, JM. Meta-datasets merging and pretreatment: LD, RD. Database search: LD, LC, CL, OM. Anatomic pathology analysis: BT, LC, OM. Immunohistochemistry: GG. Statistics: LD, LC, OM. Image analysis: LC, OM. Data analysis: LD, LC, OM. Manuscript preparation: LD, LC, OM. Manuscript editing: all authors.

## Data availability statement

Data associated with this study are available in nine Supplementary tables. Transcriptomics datasets and digital histological slides are publicly available from the Gene Expression Omnibus (GEO-NCBI), TCGA, and the Digital Slide Archive website. The MASLD meta-dataset results from the pretreatment, merging, and batch effect correction of raw transcriptomic data from publicly available datasets from different technical platforms. Meta-dataset construction has been previously described.[28] The merged user-friendly dataset can be made available from the authors upon request.

## Acknowledgements

We thank Latifa Bousarghin, Pierre-Jean Ferron, and Olivier Loréal for insightful discussions and Michèle Le Guennec, Patricia Jouas, Adina Pascu, and Thomas Poussou for logistics and administrative support.

## Supplementary data

Supplementary data to this article can be found online at https://doi.org/10.1016/j.jhepr.2023.100936.

## References

*Author names in bold designate shared co-first authorship*

[1] Eslam M, Sanyal AJ, George J. MAFLD: a consensus-driven proposed nomenclature for metabolic associated fatty liver disease. Gastroenterology 2020;158:1999–2014.e1991.
[2] Eslam M, Newsome PN, Sarin SK, et al. A new definition for metabolic dysfunction-associated fatty liver disease: an international expert consensus statement. J Hepatol 2020;73:202–209.
[3] Rinella ME, Lazarus JV, Ratziu V, et al. A multi-society Delphi consensus statement on new fatty liver disease nomenclature. J Hepatol 2023. in press.
[4] Huang R, Zhu L, Wang J, et al. Clinical features of COVID-19 patients with non-alcoholic fatty liver disease. Hepatol Commun 2020;4:1758–1768.
[5] Ghoneim S, Butt MU, Hamid O, et al.. The incidence of COVID-19 in patients with metabolic syndrome and non-alcoholic steatohepatitis: a population-based study. Metabol Open 2020;8:100057.
[6] **Hu B**, **Guo H**, Zhou P, et al. Characteristics of SARS-CoV-2 and COVID-19. Nat Rev Microbiol 2021;19:141–154.
[7] Hoffmann M, Kleine-Weber H, Schroeder S, et al. SARS-CoV-2 cell entry depends on ACE2 and TMPRSS2 and is blocked by a clinically proven protease inhibitor. Cell 2020;181:271–280 e278.
[8] Zhou P, Yang X-L, Wang X-G, et al. A pneumonia outbreak associated with a new coronavirus of probable bat origin. Nature 2020;579:270–273.
[9] Rahimi N. C-type lectin CD209L/L-SIGN and CD209/DC-SIGN: cell adhesion molecules turned to pathogen recognition receptors. Biology (Basel) 2020;10:1.
[10] **Wang Y**, **Liu S**, **Liu H**, et al. SARS-CoV-2 infection of the liver directly contributes to hepatic impairment in patients with COVID-19. J Hepatol 2020;73:807–816.
[11] Sonzogni A, Previtali G, Seghezzi M, et al. Liver histopathology in severe COVID 19 respiratory failure is suggestive of vascular alterations. Liver Int 2020;40:2110–2116.
[12] Brevini T, Maes M, Webb GJ, et al. FXR inhibition may protect from SARS-CoV-2 infection by reducing ACE2. Nature 2023;615:134–142.
[13] Arendse LB, Danser AHJ, Poglitsch M, et al. Novel therapeutic approaches targeting the renin–angiotensin system and associated peptides in hypertension and heart failure. Pharmacol Rev 2019;71:539–570.
[14] Nunes-Souza V, Alenina N, Qadri F, et al. CD36/sirtuin 1 axis impairment contributes to hepatic steatosis in ACE2-deficient mice. Oxid Med Cell Longev 2016;2016:6487509.
[15] Mercado-Gómez M, Prieto-Fernández E, Goikoetxea-Usandizaga N, et al. The spike of SARS-CoV-2 promotes metabolic rewiring in hepatocytes. Commun Biol 2022;5:827.
[16] Rajapaksha IG, Gunarathne LS, Asadi K, et al. Liver-targeted angiotensin converting enzyme 2 therapy inhibits chronic biliary fibrosis in multiple drug-resistant gene 2-knockout mice. Hepatol Commun 2019;3:1656–1673.
[17] Cai J, Zhang XJ, Li H. The role of innate immune cells in nonalcoholic steatohepatitis. Hepatology 2019;70:1026–1037.
[18] **Poisson J**, **Lemoinne S**, Boulanger C, et al. Liver sinusoidal endothelial cells: physiology and role in liver diseases. J Hepatol 2017;66:212–227.
[19] **Jeffers SA**, **Tusell SM**, Gillim-Ross L, et al. CD209L (L-SIGN) is a receptor for severe acute respiratory syndrome coronavirus. Proc Natl Acad Sci U S A 2004;101:15748–15753.
[20] Aizarani N, **Saviano A**, **Sagar**, et al. A human liver cell atlas reveals heterogeneity and epithelial progenitors. Nature 2019;572:199–204.
[21] Ramachandran P, Dobie R, Wilson-Kanamori JR, Dora EF, et al. Resolving the fibrotic niche of human liver cirrhosis at single-cell level. Nature 2019;575:512–518.
[22] Mabbott NA, Baillie JK, Brown H, et al. An expression atlas of human primary cells: inference of gene function from coexpression networks. BMC Genomics 2013;14:632.
[23] Kleiner DE, Brunt EM, Van Natta M, et al. Design and validation of a histological scoring system for nonalcoholic fatty liver disease. Hepatology 2005;41:1313–1321.
[24] Brunt EM, Janney CG, Di Bisceglie AM, et al. Nonalcoholic steatohepatitis: a proposal for grading and staging the histological lesions. Am J Gastroenterol 1999;94:2467–2474.
[25] Starmann J, Fälth M, Spindelböck W, et al. Gene expression profiling unravels cancer-related hepatic molecular signatures in steatohepatitis but not in steatosis. PLoS One 2012;7:e46584.

[26] Ahrens M, Ammerpohl O, von Schönfels W, et al. DNA methylation analysis in nonalcoholic fatty liver disease suggests distinct disease-specific and remodeling signatures after bariatric surgery. Cell Metab 2013;18:296–302.
[27] Lefebvre P, Lalloyer F, Baugé E, et al. Interspecies NASH disease activity whole-genome profiling identifies a fibrogenic role of PPARα-regulated dermatopontin. JCI Insight 2017;2:e92264.
[28] Désert R, Rohart F, Canal F, et al. Human hepatocellular carcinomas with a periportal phenotype have the lowest potential for early recurrence after curative resection. Hepatology 2017;66:1502–1518.
[29] Desquilles L, Cano L, Ghukasyan G, et al. Well-differentiated liver cancers reveal the potential link between ACE2 dysfunction and metabolic breakdown. Sci Rep 2022;12:1859.
[30] **Domovitz T**, **Ayoub S**, **Werbner M**, et al. HCV infection increases the expression of ACE2 receptor, leading to enhanced entry of both HCV and SARS-CoV-2 into hepatocytes and a coinfection state. Microbiol Spectr 2022;10:e0115022.
[31] Meijnikman AS, Bruin S, Groen AK, et al. Increased expression of key SARS-CoV-2 entry points in multiple tissues in individuals with NAFLD. J Hepatol 2021;74:748–749.
[32] Fondevila MF, Mercado-Gómez M, Rodríguez A, et al. Obese patients with NASH have increased hepatic expression of SARS-CoV-2 critical entry points. J Hepatol 2021;74:469–471.
[33] Paizis G, Tikellis C, Cooper ME, et al. Chronic liver injury in rats and humans upregulates the novel enzyme angiotensin converting enzyme 2. Gut 2005;54:1790–1796.
[34] Paizis G, Cooper ME, Schembri JM, et al. Up-regulation of components of the renin-angiotensin system in the bile duct-ligated rat liver. Gastroenterology 2002;123:1667–1676.
[35] Kristiansen TZ, Bunkenborg J, Gronborg M, et al. A proteomic analysis of human bile. Mol Cell Proteomics 2004;3:715–728.
[36] Abbas AR, Baldwin D, Ma Y, et al. Immune response in silico (IRIS): immune-specific genes identified from a compendium of microarray expression data. Genes Immun 2005;6:319–331.
[37] **Charoentong P**, **Finotello F**, **Angelova M**, et al. Pan-cancer immunogenomic analyses reveal genotype-immunophenotype relationships and predictors of response to checkpoint blockade. Cell Rep 2017;18:248–262.
[38] **Stebbing J**, **Sánchez Nievas G**, **Falcone M**, et al. JAK inhibition reduces SARS-CoV-2 liver infectivity and modulates inflammatory responses to reduce morbidity and mortality. Sci Adv 2021;7:eabe4724.
[39] Dubois-Pot-Schneider H, Fekir K, Coulouarn C, et al. Inflammatory cytokines promote the retrodifferentiation of tumor-derived hepatocyte-like cells to progenitor cells. Hepatology 2014;60:2077–2090.
[40] Poynard T, Bedossa P, Opolon P. Natural history of liver fibrosis progression in patients with chronic hepatitis C. The OBSVIRC, METAVIR, CLINIVIR, and DOSVIRC groups. Lancet 1997;349:825–832.
[41] Michaut A, Le Guillou D, Moreau C, et al. A cellular model to study drug-induced liver injury in nonalcoholic fatty liver disease: application to acetaminophen. Toxicol Appl Pharmacol 2016;292:40–55.
[42] **Varga Z**, **Flammer AJ**, Steiger P, et al. Endothelial cell infection and endotheliitis in COVID-19. Lancet 2020;395:1417–1418.
[43] Lagana SM, Kudose S, Iuga AC, et al. Hepatic pathology in patients dying of COVID-19: a series of 40 cases including clinical, histologic, and virologic data. Mod Pathol 2020;33:2147–2155.
[44] **Bradley BT**, **Maioli H**, Johnston R, et al. Histopathology and ultrastructural findings of fatal COVID-19 infections in Washington State: a case series. Lancet 2020;396:320–332.
[45] Biquard L, Valla D, Rautou PE. No evidence for an increased liver uptake of SARS-CoV-2 in metabolic-associated fatty liver disease. J Hepatol 2020;73:717–718.
[46] **Hegyi PJ**, **Váncsa S**, Ocskay K, et al. Metabolic associated fatty liver disease is associated with an increased risk of severe COVID-19: a systematic review with meta-analysis. Front Med (Lausanne) 2021;8:626425.
[47] **Cao X**, **Song LN**, Zhang YC, et al. Angiotensin-converting enzyme 2 inhibits endoplasmic reticulum stress-associated pathway to preserve nonalcoholic fatty liver disease. Diabetes Metab Res Rev 2019;35:e3123.
[48] **Yang M**, **Ma X**, Xuan X, et al. Liraglutide attenuates non-alcoholic fatty liver disease in mice by regulating the local renin–angiotensin system. Front Pharmacol 2020;11:432.
[49] Ulu A, Harris TR, Morisseau C, et al. Anti-inflammatory effects of omega-3 polyunsaturated fatty acids and soluble epoxide hydrolase inhibitors in angiotensin-II-dependent hypertension. J Cardiovasc Pharmacol 2013;62:285–297.
[50] Joshi S, Wollenzien H, Leclerc E, et al. Hypoxic regulation of angiotensin-converting enzyme 2 and Mas receptor in human $CD34^+$ cells. J Cell Physiol 2019;234:20420–20431.
[51] **Clifford BL**, **Sedgeman LR**, Williams KJ, et al. FXR activation protects against NAFLD via bile-acid-dependent reductions in lipid absorption. Cell Metab 2021;33:1671–1684 e1674.

Journal of Hepatology, Volume *6*

## Supplemental information

# SARS-CoV-2 receptor ACE2 is upregulated by fatty acids in human MASH

**Luis Cano, Lise Desquilles, Gevorg Ghukasyan, Gaëlle Angenard, Clémence Landreau, Anne Corlu, Bruno Clément, Bruno Turlin, Eric Le Ferrec, Caroline Aninat, Julie Massart, and Orlando Musso**

# SARS-CoV-2 receptor ACE2 is upregulated by fatty acids in human MASH

Luis Cano, Lise Desquilles, Gevorg Ghukasyan, Gaëlle Angenard, Clémence Landreau, Anne Corlu, Bruno Clément, Bruno Turlin, Eric Le Ferrec, Caroline Aninat, Julie Massart, Orlando Musso

Table of contents

Supplementary Methods……………………………………………………………………….. 2

Supplementary Figure Legends…………………………………………………………………. 7

Supplementary Table Legends…………………………………………………………………16

Supplementary References…………………………………………………………………...19

Supplementary Figures………………………………………………………………………..23

Supplementary Tables………………………………………………………………………...46

## Supplementary Methods

**Human primary cells***:* Gene expression was explored in the *Primary Cell Atlas*,[1] a transcriptomic microarray meta-dataset that assembles 745 samples (GSE49910). Expression data were quantile-normalized and top ten differentially expressed samples across tissues were plotted.

## Immunohistochemistry

Routine formalin-fixed, paraffin-embedded tissue blocks from the Anatomic Pathology laboratory, Rennes University Hospital, were used to prepare 5 µm sections mounted on Super Frost Plus slides (VWR International). Antibodies used, incubation conditions, horseradish peroxidase and tyramide-based signal amplification are summarized in Table S2. Digital images were obtained with either a single focus (Nanozoomer, Hamamatsu Photonics) or confocal (3D-Histech) slide scanner. Image analysis quantitation was performed with the HALO Spatial Analysis software (Indica Labs). Immunohistochemistry, digital imaging and data acquisition were performed in an ISO-9001-certified core facility (High Precision Histopathology, H2P2, CNRS 3840, INSERM 018, Rennes 1 University). Data analysis was performed with R 3.6.2 and the R package dplyr 0.8.5 for data cleaning and transformation. The Mann-Whitney U test was used to assess the differences between means.

## Cell culture and treatments

The Human Macrovascular Endothelial cell line (HMEC-1) was obtained and cultured as previously described.[2] Briefly, cells were routinely cultured in MCDB-131 medium

with hydrocortisone (1 μg/ml), epidermal growth factor (10 ng/ml), penicillin (50 units/ml), streptomycin (50 units/ml), L-glutamine (10 mM) and with 10% foetal bovine serum. Cells were treated with the indicated cytokines or LPS at 90% confluence after overnight culture in serum-free medium.

**Primary human hepatocyte isolation and culture**

Primary human adult hepatocytes from non-tumor histologically normal liver fragments were obtained from Biopredic International (Saint-Grégoire, France). Donors were patients undergoing resection of primary or liver secondary tumors. Prior to liver tissue perfusion and dissociation for hepatocyte isolation, the hepatectomy specimen was examined by an anatomic pathologist. Written informed consent was obtained from the patients. The procedure, approved by INSERM's Review Board and the local Ethics Committee, was performed as described.[3]

**Primary human hepatocyte treatments**

*Treatment of primary human hepatocytes with cytokines and LPS:*

Hepatocytes were seeded at a density of 250,000 cells/cm$^2$ in 24-well plates and fed with Williams' E medium supplemented with 10 % foetal calf serum (FCS), 100 units/ml penicillin, 100 μg/ml streptomycin, 5 μg/ml insulin, 2 mM glutamine, and 50 μM hydrocortisone hemisuccinate. One day after seeding, the medium was complemented with 2% DMSO and renewed daily. Human hepatocytes were used three to five days after seeding. Treatments with cytokines (IL-6, 20 ng/ml; IL-1B, 10 ng/ml; TNFA, 100 ng/ml) or LPS (10 ng/ml) were performed in FCS- and DMSO-deprived medium. Cells

were harvested after 12 hr of treatment. Human recombinant IL-6, IL1B and TNFA were purchased from Biotechne (R&D Systems) and LPS *(E. coli* 055:B5) *was* from Sigma.

*Treatment of primary human hepatocytes with fatty acids:*

Cells were seeded in collagen-coated 24-well plates at a density of $0.4.10^6$ cells/well and cultured at 37°C with 5% CO2 and saturating humidity in William's E medium supplemented with 5% FCS, 100 U/ml penicillin, 100 µg/ml streptomycin, 2 mM glutamine, 5 µg/ml insulin and 1 µM hydrocortisone hemisuccinate. After cell adherence to the culture plates (24 hr), hepatocytes were treated every other day for 7 days with 150 µM oleic acid (C18:1) or 150 µM oleic acid + 150 µM stearic acid (C18:1+C18:0). Fatty acid stock solutions (5 mM) were prepared in heated NaOH 0.1 M and then diluted in prewarmed 10% fatty acid-free BSA solution in serum-free William's E medium. Control cell medium was supplemented with the same amount of NaOH/FFA-free BSA mixture.

**Neutral lipid staining**

Cells were incubated for 15 min at 37°C with 2 µM BODIPY 493/503 (Molecular Probes, ThermoFisher Scientific), fixed with formalin (Sigma-Aldrich) for 1 hr and stained with 1 µg/ml Hoechst 33342 (ThermoFisher Scientific) for 20 min.

**RNA extraction and Real-time PCR**

For primary human hepatocytes seeded in plastic (see cytokine and LPS treatments above), total RNA was extracted with the Nucleospin RNA isolation kit from Macherey-Nagel (Düren, Germany), with DNAse treatment, followed by analysis of RNA concentration and 260/280 and 230/260 ratios with a Nanodrop 1000 (Thermo Fisher Scientific, Waltham, MA). RNA was reverse-transcribed using the High-Capacity cDNA Reverse Transcription kit (Applied Biosystems, Woolston, UK). Gene expression was measured by real-time PCR using SYBR Green PCR Master Mix (Applied Biosystems) and a 384-well-QuantStudio 7 Flex Real-Time PCR System (Thermo Fisher). Primer sequences are shown in Table S3. *HPRT1* or *TBP* were used as housekeeping genes and results analyzed by the $2^{-\Delta\Delta Ct}$ method using control conditions as calibrators.

For primary human hepatocytes seeded in collagen (see fatty acid treatments above), total RNA was isolated with Trizol according to the manufacturer's recommendations (Life Technologies). Total RNA concentration was quantified spectrophotometrically (NanoDrop ND-1000 Spectrophotometer, ThermoFisher Scientific, Waltham, MA). RNA was reverse-transcribed to cDNA using the High Capacity cDNA RT kit. Gene expression was determined by real-time PCR using SYBR Green (Life Technologies), applying the the $2^{-\Delta\Delta Ct}$ method and using *HPRT1* gene expression as an internal standard. Primer sequences are shown in Table S3.

**Deconvolution of immune cell populations from transcriptomic data in fatty liver disease**

Deconvolution of immune cell populations to construct phenetic diagrams (i.e., immune phenotypic scoring or immunophenoscoring) was done by applying previously

described algorithms.[4] Data were processed using the R script provided by the authors (https://github.com/icbi-lab/Immunophenogram). An alternative approach to infer the importance of leucocyte cell populations infiltrating fatty liver disease samples was to search for expression of immune cell subset marker RNA using the Immune Response in Silico (IRiS)[5] microarray dataset.

**Statistical analysis**

Correlations between genes were calculated using either the Pearson's or Spearman's R methods. Continuous variables were compared by parametric testing (Student's *t*-test and ANOVA) or non-parametric testing (Mann-Whitney U test); whereas categorical data were compared with Fisher's exact test. *P* values were adjusted for multiple testing using the Benjamini-Hochberg procedure.

Weighted Gene Correlation Network Analysis (WGCNA) was performed using the *WGCNA[6]* R package, as we previously described.[7] The network file was then exported to Cytoscape.[8] Functional analysis and GO enrichment were conducted after gene module detection and results were summarized using Express Analysis from Metascape[9] or REVIGO,[10] as indicated.

**Supplementary Figure Legends**

**Fig. S1.** Positive controls for ACE2 (A, B) and TMPRSS2 (C, D) antibodies. Immunoperoxidase staining *(brown)* and light hematoxylin counterstaining *(blue),* in kidney (A), intestine (B) and prostate (C, D). In the kidney, ACE2 positive signal is seen in the parietal cell layer of the Bowman's capsule and the convoluted tubules (A). ACE2 is detected in the apical pole of the intestinal epithelium (B). TMPRSS2 is detected in the apical pole of the glandular epithelium and in surrounding smooth muscle (C, D). Digital slides were acquired in a slide scanner with a X20 objective (Nanozoomer, Hamamatsu Photonics).

**Fig. S2.** (A) *ACE2* and *TMPRSS2* mRNA expression data extracted from the Primary Cell Atlas meta-database,[1] which assembles 745 primary cell samples from over 100 studies, exploited as described.[11, 12] Both *ACE2* and *TMPRSS2* mRNAs are detected in primary human hepatocytes. (B, C) ACE2 expression in normal human liver. (B) In a portal tract, ACE2 is detected in bile ducts at low magnification *(arrows).* (C) Higher power view of the region of interest framed in *red* in *B.* ACE2 signal in a bile duct *(red arrowhead),* in vessels within the portal tract and along the limiting plate *(green arrowheads)*, as well as in cells lining the sinusoids *(black arrowheads).* (D-G) Co-expression of ACE2 and TMPRSS2 in hepatocytes. ACE2 *(red, TRITC),* TMPRSS2 *(orange, Cy5)* and the hepatocyte marker HepPar-1 *(green, FITC)* were detected with a microscope scanner and a X40 objective. Images are Z-stacks of four 500 nm focusing steps.

**Fig. S3. ACE2 is not detected in CD45 positive leukocytes.** Co- staining of ACE2 *(red, TRITC)* and CD45 *(green, FITC)* in the liver parenchyma. (A-C) At high magnification, signal in *yellow* corresponds to overlapping endothelial and leukocyte cell membranes. Immunofluorescence digital images were acquired with a microscope scanner using a X40 objective (Nanozoomer, Hamamatsu Photonics). Images are Z-stacks of four 500 nm focusing steps.

**Fig. S4A-D. ACE2 and CD68 in liver sinusoids.** Co-staining of ACE2 *(red, TRITC)* and CD68 *(green, FITC)* in the liver parenchyma. (A-D) Overlaps of both epitopes are seen in *yellow* in some cells (shown in *D).* Immunofluorescence digital images were acquired with a confocal microscope scanner and a X40 objective. Images are Z-stacks of four 500 nm focusing steps.

**Fig. S4E-G. ACE2 and CD68 in liver sinusoids.** Co-staining of ACE2 *(red, TRITC)* and CD68 *(green, FITC)* in the liver parenchyma. (A-D) Overlaps of both epitopes are seen in *yellow* in some cells (shown in *G).* Image deconvolution across 500 nm steps in the Z axis shows that CD68 (+) Kupffer cell membrane extensions line ACE2 (+) sinusoidal endothelial cells. Immunofluorescence digital images were acquired with a confocal microscope scanner and a X40 objective. Images are Z-stacks of four 500 nm focusing steps.

**Fig. S5. ACE2 is not detected in CD3 positive lymphocytes or in ACTA2-(a.k.a alpha smooth muscle actin)-positive myofibroblasts.** Co-staining of

ACE2 *(red, TRITC)* and CD3 *(green, FITC)* in the liver parenchyma (A-B) or ACE2 and ACTA2 *(green, FITC)* at the interface between the liver parenchyma and a fibrous septum (C). Immunofluorescence digital images were acquired with a microscope scanner using a X40 objective (Nanozoomer, Hamamatsu Photonics, in *A)* or with a confocal microscope scanner and a X40 objective. Images are Z-stacks of four 500 nm focusing steps.

**Fig. S6.** Single cell RNA sequencing data from histologically normal liver samples obtained from nine patients undergoing resection of colorectal cancer metastases or cholangiocarcinoma, without underlying liver disease. Data were obtained from Aizarani et al.,[13] through a dedicated web interface (http://human-liver-cell-atlas.ie-freiburg.mpg.de/). Arrows show *ACE2* mRNA expression in comparison with *TMPRSS2, FURIN, DPP4* and albumin *(ALB)* mRNAs; the sinusoidal endothelial cell *(SEC)* marker *CLEC4M;* the periportal *(PP)* markers *PCK1, ALDOB, HAL;* the perivenous *(PV)* marker *GLUL;* the cholangiocyte *(Cho)* and liver progenitor cell markers *EPCAM, KRT19, SOX9;* the capillary endothelial cell *(Endo)* marker *CD34;* the bile canaliculi *(Canaliculi)* marker *ABCC2*; the myofibroblast *(Myof)* marker *ACTA2*; the liver basement membrane *(BM)* markers *COL4A1 and LAMC1;* the monocyte *(Mo)* marker *CD163;* the Kupffer cell marker *ITGAM;* the B lymphocyte marker *CD37;* the T lymphocyte marker *CD3D;* the NK cell markers *CCL4* and *KLRF1* and the NKT cell marker *CCL5.* $Log_2$ expression intensities are indicated by a heat scale. Overall, *ACE2* mRNA levels are low in all cell populations tested, but detected in scattered cells in the clusters containing sinusoidal endothelial cells; periportal and perivenous hepatocytes and cholangiocytes.

**Fig. S7**. Single cell RNA sequencing data from five normal *(uninjured)* and five cirrhotic human livers (2x NAFLD, 2x alcohol-related liver disease, 1x primary biliary cirrhosis), extracted from the Ramachandran *et al.* dataset,[14] through a dedicated web interface (https://shiny.igmm.ed.ac.uk/livercellatlas/), where violin plots can be obtained from integrated Seurat functions (ggplot2, pheatmap and grid R packages).[14] (A) *ACE2,* the sinunoidal endothelial cell marker *CLECL4M, albumin (ALB)*, the hepatocyte marker *Cytokeratin 18 (KRT18)* and the myofibroblast marker *alpha smooth muscle actin (ACTA2)* in MP, mononuclear phagocytes; pDC, plasmocytoid dendritic cells; ILC, innate lymphoid cells; Tc, T lymphocytes; Bc, B lymphocytes; Plc, plasma cells; MC, mast cells; Endo, endothelial cells; Mes, mesenchymal cells; Meso, mesothelial cells; Hc, hepatocytes; Cho, cholangiocytes. (B) *ACE2,* the capillary endothelial cell marker *CD34* and the liver sinusoidal endothelial cell marker *CLEC4M* in the indicated cell populations. (C) *TMPRSS2, FURIN* and *DPP4* in the indicated cell populations.

**Fig. S8**. (A) Merging of three transcriptomic microarray datasets into a fatty liver disease meta-dataset of 243 human liver samples: GSE33814,[15] consists of 12 normal livers, 19 steatoses, and 12 steatohepatitides; GSE48452[16] consists of 14 normal livers, 27 livers from obese patients, 14 steatoses and 18 non-alcoholic steatohepatitides (MASH); GSE83452[17] consists of 231 samples from which 126 MASHs were extracted for study. Altogether, the meta-dataset includes 27 normal livers; 27 livers from obese patients; 33 steatoses; 12 steatohepatitides and 144 MASHs. Batch effect was corrected using the ComBat algorithm (*sva* R package). Raw expression data were quantile-normalized and log2-transformed. (B) Expression of the

SARS-CoV-2 co-receptor TMPRSS2, the MERS-CoV receptor DPP4 and the sinusoidal endothelial cell marker and SARS-CoV-2 co-receptor *CLEC4M* in the fatty liver disease meta-dataset. Statistical significance of the difference between groups was calculated with analysis of variance (ANOVA) followed by Tuckey post-hoc test *(*, p<0.05; **, p<0.01; ***, p<0.001)*.

**Fig. S9.** RNA expression of immune cell subset-specific markers in circulating immune cells isolated with cell-surface-specific antibodies and analyzed by cDNA microarrays. Raw data were extracted from the GSE22886 dataset.[5] Only values above the mean expression level for each probe were analyzed.

**Fig. S10**. ***ACE2, TMPRSS2* and *DPP4* mRNAs are detected at very low levels in circulating immune cells.** (A) Expression of *ACE2, TMPRSS2* and *DPP4* mRNAs in specifically isolated circulating immune cell subsets from the GSE22886[5] dataset. The number of observations in each cell subset is shown in Table S9. (B) The expression of *ACE2, TMPRSS2* and *DPP4* mRNAs in fatty disease liver tissues correlates with immune cell markers in 13 normal livers,

19 steatoses and 12 steatohepatites in the transcriptomic microarray dataset GSE33814. Immune cell populations in liver tissues were deconvoluted as shown in Fig. S10, according to the GSE22886 dataset.[5] The color scale for the heatmap of Pearson’s correlation coefficients and the color codes for the immune cell populations are shown on the right.

**Fig. S11.** (A) *ACE2, TMPRSS2* and *DPP4* mRNA levels correlate with markers of specific immune cell subsets in 14 normal livers, 27 livers from obese patients, 14 steatoses and 18 MASHs in the GSE48452[16] transcriptomic dataset. The color scale for the heatmap of Pearson's correlation coefficients and the color codes for immune cell populations are shown on the right. (B) MASH samples from the GSE48452[16] dataset show increased expression of molecular markers for T cells. ANOVA was followed by Tuckey post-hoc test to assess the statistical significance of the difference between *control* and *obesity* or *steatosis* or *MASH (*, p<0.05).*

**Fig. S12.** (A) Immunophenogram classifying immune reactivity parameters in four immunogenicity functional families: MHC, Major Histocompatibility Complex; EC, Effector Cells; SC, Suppressor Cells; CP, Check Points. Enhancement/suppression of immune reactivity are indicated by (+)/(–). Parameters' full names and gene symbols are indicated in Table S5. (B-D) Immunophenograms for each liver sample from the GSE33814 dataset. (E) Parameters' Z-scores and functional families are shown as red/blue heat map and grayscale, respectively.

**Fig. S13.** Relatedness dendrogram (A) and correlation heatmap (B) of modules identified by Weighted Gene Coexpression Network Analysis (WGCNA) in the fatty liver disease metadataset of 243 human liver samples, which includes 27 normal livers; 27 livers from obese patients; 33 steatoses; 12 steatohepatites and 144 MASHs. After network analysis with the *WGCNA* R package,[6] the dendrogram shows the eigengene relatedness between modules. Modules are sub-networks of highly

correlated genes. The eigengene represents the first principal component of each module transcriptome across samples. The heatmap shows the pairwise correlation coefficients of module eigengenes, with *red* representing highly positive; *blue* highly negative and *white* no pairwise correlations. The *blue box* indicates three highly correlated modules that are enriched in steatohepatitis *(see Fig. S14).* Color coding of module names is arbitrary and unrelated to phenotype.

**Fig. S14.** Matrix of module-trait relationship after analysis of 27 normal livers; 27 livers from obese patients; 33 steatoses; 12 steatohepatites and 144 MASHs by WGCNA. The purpose of this matrix is to identify the gene expression modules that are the most highly associated with at least one of the patient groups. Correlation coefficients and statistical significance are indicated within each cell of the matrix. They are color-coded according to the heatmap on the right, where *red* represents highly positive correlation; *blue* highly negative correlation and *white* no correlation. The *blue frame* highlights the three modules the most highly associated with steatohepatitis. The number of patients in each group is indicated below. Color coding of module names is arbitrary and unrelated to phenotype.

**Fig. S15.** Representation of the three most highly correlated gene network modules *(grey; red; sky blue).* The hub gene is colored in *pink* at the center of the module. It corresponds to the node with the highest number of connections *(black lines)*. Node size is proportional to the number of connections*.* The network files for each module (Table S7) were exported to Cytoscape[8] for graphical representation.

**Fig. S16.** Functional analysis and gene ontology enrichment of the three gene network modules *(grey; red; sky blue)* the most highly correlated with steatohepatitis. Results were summarized using *Express Analysis* from Metascape.[9]

**Fig. S17.** (A) Expression of the indicated genes in alcoholic hepatitis *versus* control livers in the GSE28619 transcriptomic dataset.[18] *TIMP1* and *CST3* are shown as markers of fibrosis and inflammation, respectively. (B) Expression of *ACE2* mRNA in peripheral blood mononuclear cells in response to acute HCV infection, according to the GSE119117 RNA sequencing dataset shows no significant variation in *ACE2* mRNA levels.[19] (C) Pegylated interferon alpha 2B –but not alpha 2A, decreases *ACE2* mRNA expression in 30 HCV patients, according to the GSE48445 microarray dataset.[20] (D) Pegylated interferon alpha 2A plus adefovir do not change *ACE2* mRNA levels in 15 HBV patients regardless of the HBeAg and responder/non responder status, according to the GSE54747 microarray dataset.[21]

**Fig. S18.** Primary hepatocytes from three patients were treated with IL6 (20 ng/ml), IL1B and LPS (10 ng/ml) and TNFA (100 ng/ml) without serum during 12 hr. Hepatocytes were seeded and treated in triplicate wells. RNA from each well was analyzed in duplicate by real time RT-PCR using the $2^{-\Delta\Delta Ct}$ method. Bars indicate means ± SD. Statistical intergroup differences were assessed by Kruskal-Wallis *(KW)* ANOVA as indicated, followed by the post-hoc Dunn's multiple comparison test comparing control with cytokine/LPS treatment (*, p<0.05; **, p<0.01; ***, p<0.001).

**Fig. S19.** Human microvascular endothelial cells (HMEC-1 cell line) were treated with IL6 (20 ng/ml), IL1B, LPS (10 ng/ml) or TNFA (100 ng/ml) without serum during 12 hr. Cells were seeded and treated in duplicate wells. RNA from each well was analyzed in duplicate by real time RT-PCR using the $2^{-\Delta\Delta Ct}$ method. Bars indicate means ± SD. *HMEC_1* and *HMEC_2* denote two separate experiments. *CXCL8* and *CCL2* mRNA expression are shown as positive controls of chemokine induction in response to the applied treatment.

**Fig. S20.** *ACE2* mRNA expression in the GSE48452 dataset consisting of transcriptomic microarray data from 12 controls (lean patients without MASLD), 16 obese patients without MASLD (healthy obese), 17 MASH and 9 patients with steatosis. Box-and-whisker plots represent median plus $1^{st}$ and $3^{rd}$ quartiles. Vertical bars represent [1.5 x interquartile range, above the $3^{rd}$ quartile] and [1.5 x interquartile, range below the $1^{st}$ quartile]. Outliers (present only in MASH and steatosis as colored dots) indicate values > 1.5 folds and < 3 folds the interquartile range above and below the boxes, respectively. The GSE48452 was explored using RMA-normalized expression data (*oligo* R package), as described.[22] Samples obtained after bariatric surgery were excluded from the analysis. All pairwise comparisons were performed using the non-parametric Wilcoxon's test (*stats* R package). *P* values were adjusted for multiple testing using the Benjamini-Hochberg's procedure.

**Supplementary Table Legends**

**Table S1.** Patient clinical and biological data; steatosis, ballooning, inflammation, NAFLD Activity Score (NAS) and MASH grading. NAS assesses active injury defined by the sum of the scores for steatosis (0-3), lobular inflammation (0-3) and ballooning (0-2); thus ranging from 0 to 8.[23] MASH grading assesses the necro- inflammatory activity for steatohepatitis as mild (grade 1); moderate (grade 2) and severe (grade 3), as described.[24] The control case n° U1241_10 could have been diagnosed as MASLD on the basis of the hepatic steatosis index[25], plus a BMI >25 kg/m$^2$ and type II diabetes. However, histological assessment of the partial hepatectomy specimen revealed <5% steatosis, mild portal fibrosis and no inflammation. Thus, liver histology for this case was not compatible with MASLD, NAFLD or MASH diagnostic criteria.[26, 27]

**Table S2.** Antibodies and immunohistochemical methods.

**Table S3.** Oligonucleotides used for real-time PCR.

**Table S4.** Numbers of available observations for the listed genes after meta-dataset pretreatment. The meta-dataset was constructed after merging the following datasets: GSE33814; GSE48452; GSE83452. Quantile normalization and batch effect correction were applied, as shown in Supplementary Figure 8A.

**Table S5.** Full names and gene symbols for immunogenicity functional families shown in Fig. 5A and Fig. S12. Positive and negative modulators are highlighted by red/green cell coloring, respectively.

**Table S6.** Statistical significance of the differences between mean expression levels of immunophenoscore parameters shown in Fig. 5A and Fig. S12. Positive and negative modulators are highlighted by red/green cell coloring, respectively.

**Table S7.** Excel file containing three spreadsheets named after the three color-coded names of the modules associated with steatohepatitis, as shown in Supplementary Figs. 13-16. Module names are: *Grey60*; *Red* and *Skyblue*. Spreadsheet columns headings: *"names",* gene symbols. *"Nb_edges",* measures the number of genes with which a given gene is correlated. *"Gene_Significance",* measures the biological significance of a given gene in a 0 to 1 range; the higher the absolute value, the more biologically significant a given gene is. Gene significance can take on positive or negative values. *"Module_Membership",* measures the correlation of a given gene with the first principal component of the module in a 0 to 1 absolute range. Module membership can take on positive or negative values. Highly connected hub genes will have high module membership values. Measures were obtained by Weighted Gene Correlation Network Analysis (WGCNA) in the fatty liver disease metadataset of 243 human liver samples, which includes 27 normal livers; 27 livers from obese patients; 33 steatoses; 12 steatohepatites and 144 MASHs.

**Table S8. METAVIR[28] liver fibrosis and inflammation scores in 41 non-tumor livers from the TCGA-LIHC dataset.** Digital hematoxylin-eosin-stained slides were accessed from the publicly available platform Cancer Digital Archive website (https://cancer.digitalslidearchive.org/). Histological analysis was performed by two observers trained in liver pathology (OM & LC), blinded to molecular profiling. Anonymized IDs are provided in the 1st column. Assessed features are indicated as column headings. Normalized mRNA expression values for *ACE2, COL1A1, COL3A1* and *VCAN* are indicated.

**Table S9.** Number of observations in each cell subset from the GSE22886 IRiS Immune Response in Silico dataset.

**Supplementary References**

[1] Mabbott NA, Baillie JK, Brown H, Freeman TC, Hume DA. An expression atlas of human primary cells: inference of gene function from coexpression networks. BMC Genomics 2013;14:632.

[2] Le Goff M, Lagadic-Gossmann D, Latour R, Podechard N, Grova N, Gauffre F, et al. PAHs increase the production of extracellular vesicles both in vitro in endothelial cells and in vivo in urines from rats. Environ Pollut 2019;255:113171.

[3] Guguen-Guillouzo C, Campion JP, Brissot P, Glaise D, Launois B, Bourel M, et al. High yield preparation of isolated human adult hepatocytes by enzymatic perfusion of the liver. Cell Biol Int Rep 1982;6:625-628.

[4] Charoentong P, Finotello F, Angelova M, Mayer C, Efremova M, Rieder D, et al. Pan-cancer Immunogenomic Analyses Reveal Genotype-Immunophenotype Relationships and Predictors of Response to Checkpoint Blockade. Cell Rep 2017;18:248-262.

[5] Abbas AR, Baldwin D, Ma Y, Ouyang W, Gurney A, Martin F, et al. Immune response in silico (IRIS): immune-specific genes identified from a compendium of microarray expression data. Genes & Immunity 2005;6:319-331.

[6] Langfelder P, Horvath S. WGCNA: an R package for weighted correlation network analysis. BMC Bioinformatics 2008;9:559.

[7] Desquilles L, Musso O. Metabolic Networks: Weighted Gene Correlation Network Analysis. Methods Mol Biol 2023;2675:317-325.

[8] Shannon P, Markiel A, Ozier O, Baliga NS, Wang JT, Ramage D, et al. Cytoscape: a software environment for integrated models of biomolecular interaction networks. Genome Res 2003;13:2498-2504.

[9] Zhou Y, Zhou B, Pache L, Chang M, Khodabakhshi AH, Tanaseichuk O, et al. Metascape provides a biologist-oriented resource for the analysis of systems-level datasets. Nat Commun 2019;10:1523.

[10] Supek F, Bosnjak M, Skunca N, Smuc T. REVIGO summarizes and visualizes long lists of gene ontology terms. PloS one 2011;6:e21800.

[11] Desert R, Mebarki S, Desille M, Sicard M, Lavergne E, Renaud S, et al. "Fibrous nests" in human hepatocellular carcinoma express a Wnt-induced gene signature associated with poor clinical outcome. Int J Biochem Cell Biol 2016;81(Pt A):195-207.

[12] Mebarki S, Desert R, Sulpice L, Sicard M, Desille M, Canal F, et al. De novo HAPLN1 expression hallmarks Wnt-induced stem cell and fibrogenic networks leading to aggressive human hepatocellular carcinomas. Oncotarget 2016;7:39026-39043.

[13] Aizarani N, Saviano A, Sagar, Mailly L, Durand S, Herman JS, et al. A human liver cell atlas reveals heterogeneity and epithelial progenitors. Nature 2019;572:199-204.

[14] Ramachandran P, Dobie R, Wilson-Kanamori JR, Dora EF, Henderson BEP, Luu NT, et al. Resolving the fibrotic niche of human liver cirrhosis at single-cell level. Nature 2019;575:512-518.

[15] Starmann J, Falth M, Spindelbock W, Lanz KL, Lackner C, Zatloukal K, et al. Gene expression profiling unravels cancer-related hepatic molecular signatures in steatohepatitis but not in steatosis. PloS one 2012;7:e46584.

[16] Ahrens M, Ammerpohl O, von Schönfels W, Kolarova J, Bens S, Itzel T, et al. DNA methylation analysis in nonalcoholic fatty liver disease suggests distinct disease-specific and remodeling signatures after bariatric surgery. Cell Metab 2013;18:296-302.

[17] Lefebvre P, Lalloyer F, Baugé E, Pawlak M, Gheeraert C, Dehondt H, et al. Interspecies NASH disease activity whole-genome profiling identifies a fibrogenic role of PPARα-regulated dermatopontin. JCI Insight 2017;2:6.

[18] Affo S, Dominguez M, Lozano JJ, Sancho-Bru P, Rodrigo-Torres D, Morales-Ibanez O, et al. Transcriptome analysis identifies TNF superfamily receptors as potential therapeutic targets in alcoholic hepatitis. Gut 2013;62:452-460.

[19] Rosenberg BR, Depla M, Freije CA, Gaucher D, Mazouz S, Boisvert M, et al. Longitudinal transcriptomic characterization of the immune response to acute hepatitis C virus infection in patients with spontaneous viral clearance. PLoS Pathog 2018;14:e1007290.

[20] Dill MT, Makowska Z, Trincucci G, Gruber AJ, Vogt JE, Filipowicz M, et al. Pegylated IFN-alpha regulates hepatic gene expression through transient Jak/STAT activation. J Clin Invest 2014;124:1568-1581.

[21] Jansen L, de Niet A, Makowska Z, Dill MT, van Dort KA, Terpstra V, et al. An intrahepatic transcriptional signature of enhanced immune activity predicts response to peginterferon in chronic hepatitis B. Liver Int 2015;35:1824-1832.

[22] Carvalho BS, Irizarry RA. A framework for oligonucleotide microarray preprocessing. Bioinformatics 2010;26:2363-2367.

[23] Kleiner DE, Brunt EM, Van Natta M, Behling C, Contos MJ, Cummings OW, et al. Design and validation of a histological scoring system for nonalcoholic fatty liver disease. Hepatology 2005;41:1313-1321.

[24] Brunt EM, Janney CG, Di Bisceglie AM, Neuschwander-Tetri BA, Bacon BR. Nonalcoholic steatohepatitis: a proposal for grading and staging the histological lesions. Am J Gastroenterol 1999;94:2467-2474.

[25] Lee JH, Kim D, Kim HJ, Lee CH, Yang JI, Kim W, et al. Hepatic steatosis index: a simple screening tool reflecting nonalcoholic fatty liver disease. Dig Liver Dis 2010;42:503-508.

[26] Eslam M, Sanyal AJ, George J. MAFLD: A Consensus-Driven Proposed Nomenclature for Metabolic Associated Fatty Liver Disease. Gastroenterology 2020;158:1999-2014.e1991.

[27] Eslam M, Newsome PN, Sarin SK, Anstee QM, Targher G, Romero-Gomez M, et al. A new definition for metabolic dysfunction-associated fatty liver disease: An international expert consensus statement. J Hepatol 2020;73:202-209.

[28] Poynard T, Bedossa P, Opolon P. Natural history of liver fibrosis progression in patients with chronic hepatitis C. The OBSVIRC, METAVIR, CLINIVIR, and DOSVIRC groups. Lancet 1997;349:825-832.

## Supplementary Figures

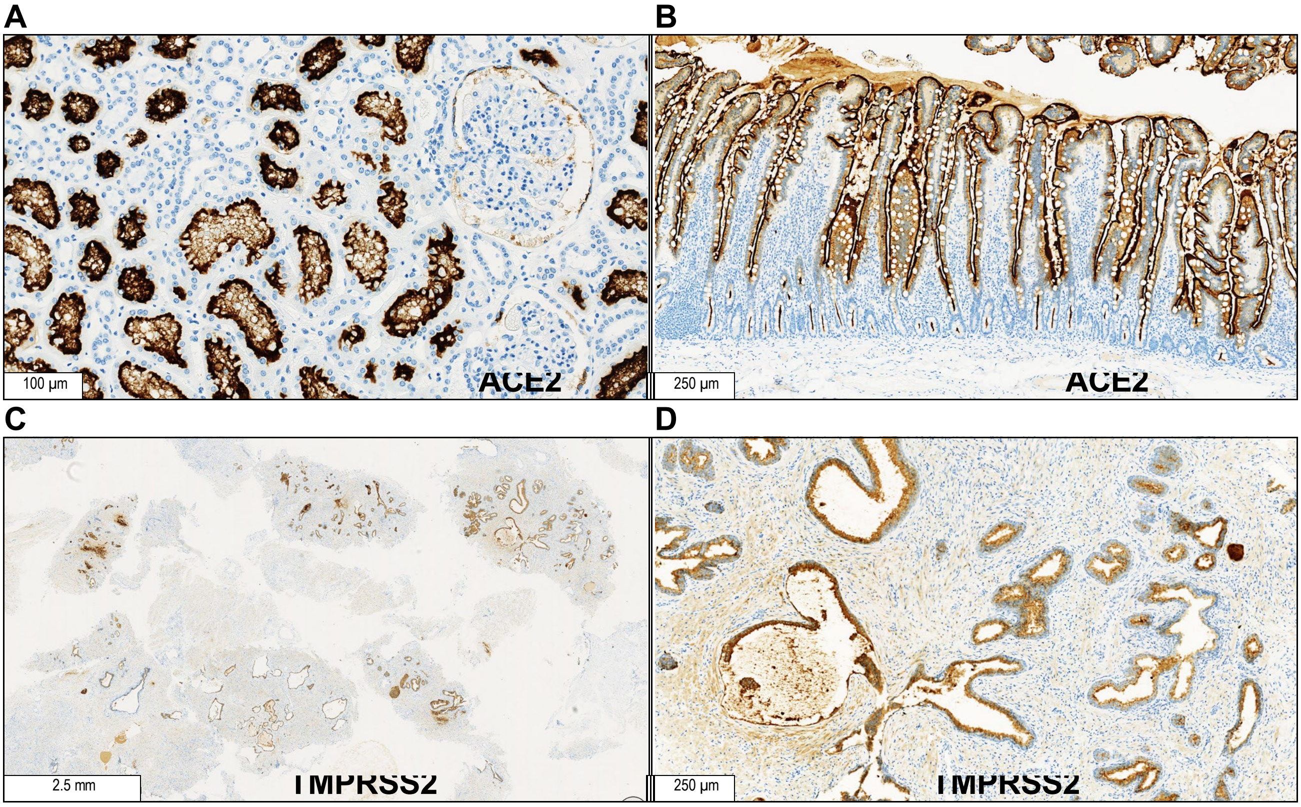


Cano et al., Fig. S1

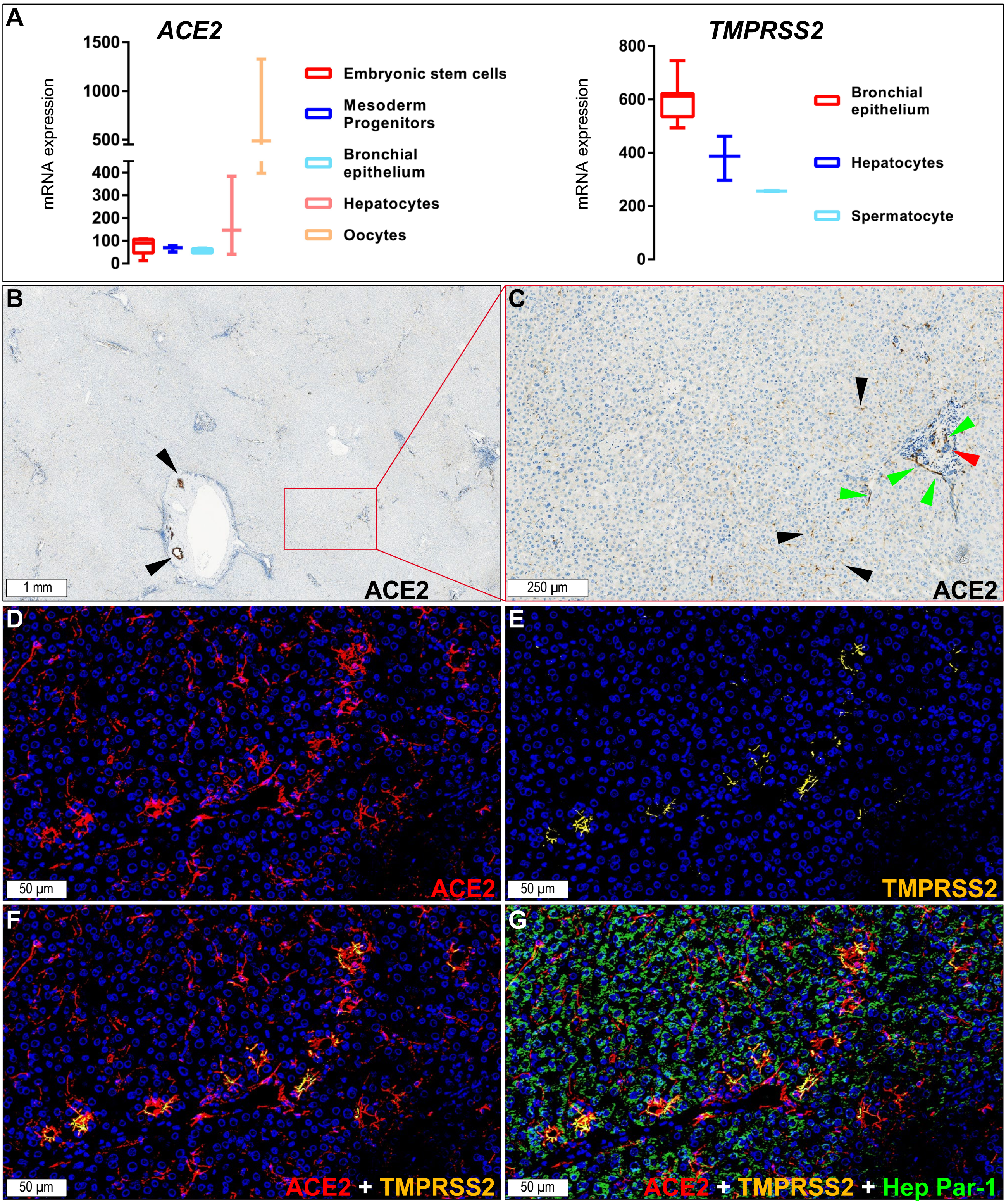


Cano et al., Fig. S2

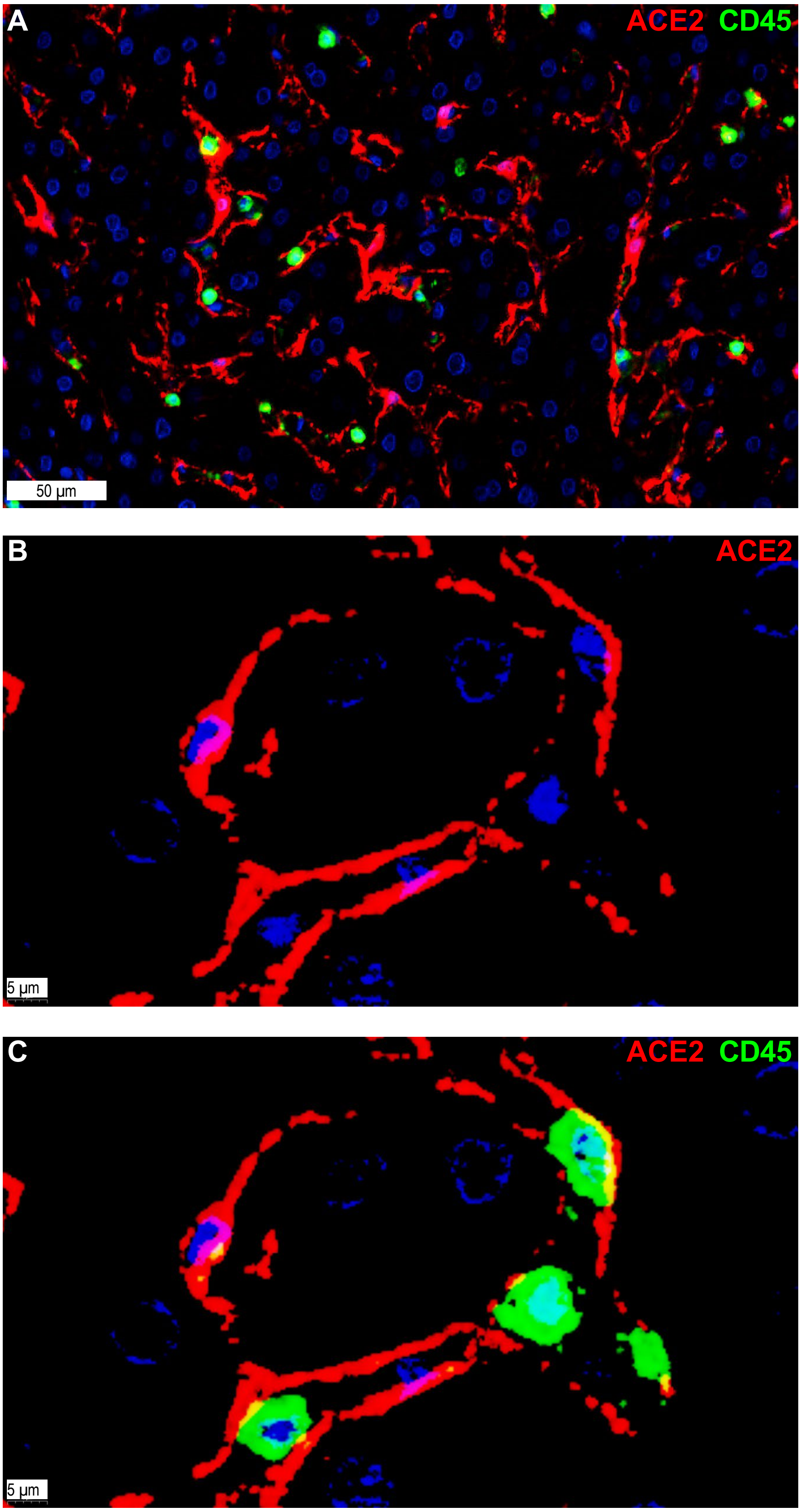


Cano et al., Fig. S3

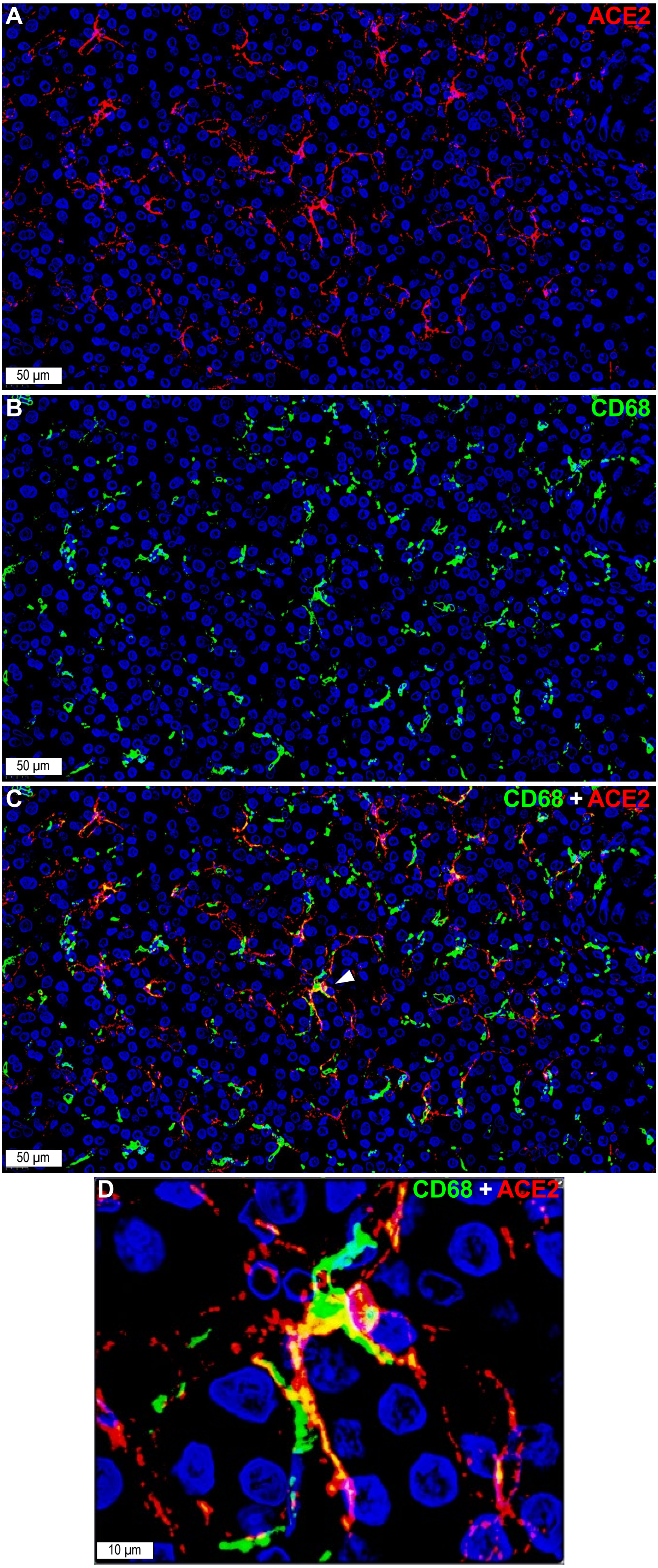


Cano et al., Fig. S4A-D

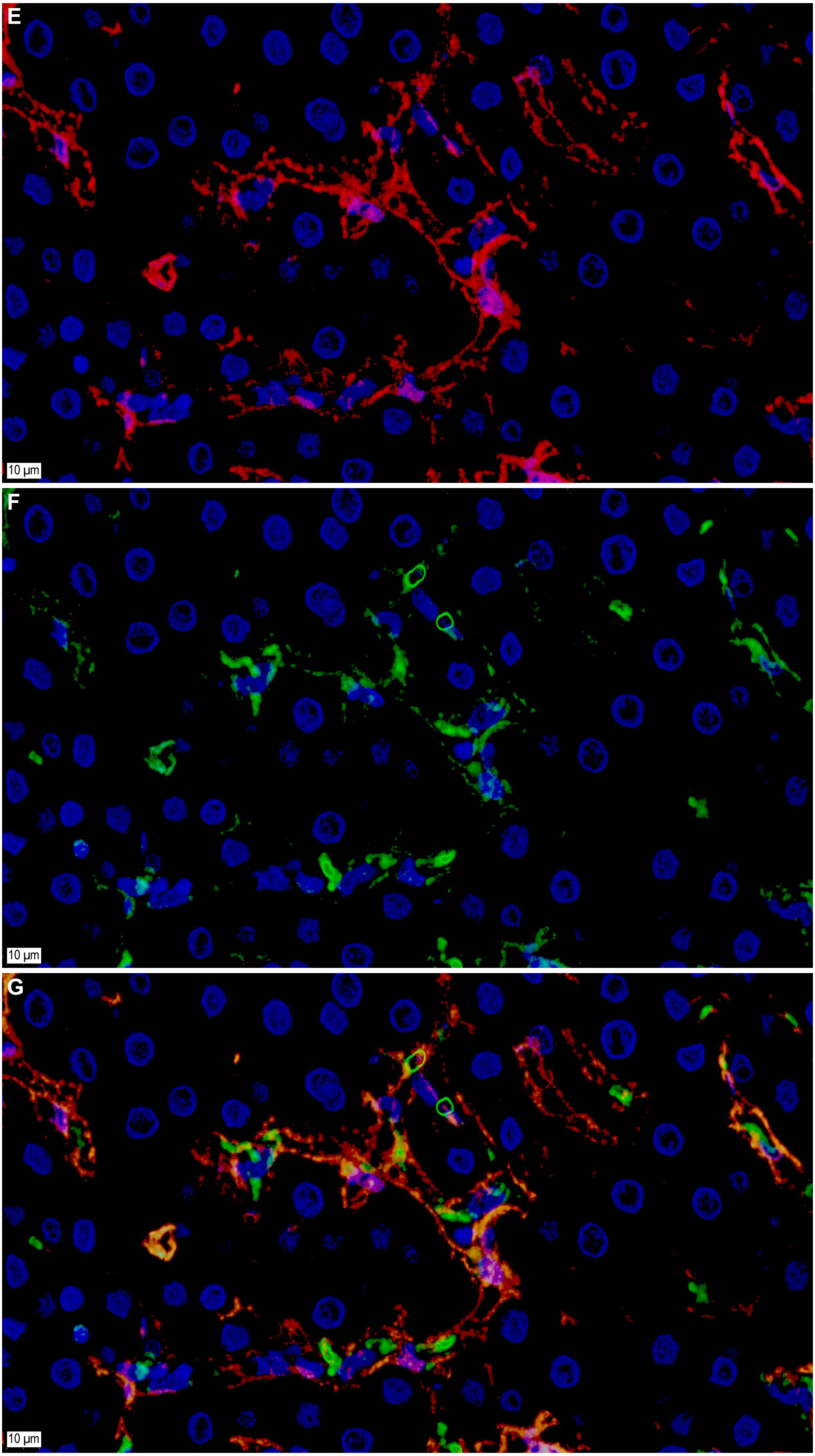


Cano et al., Fig. S4E-G

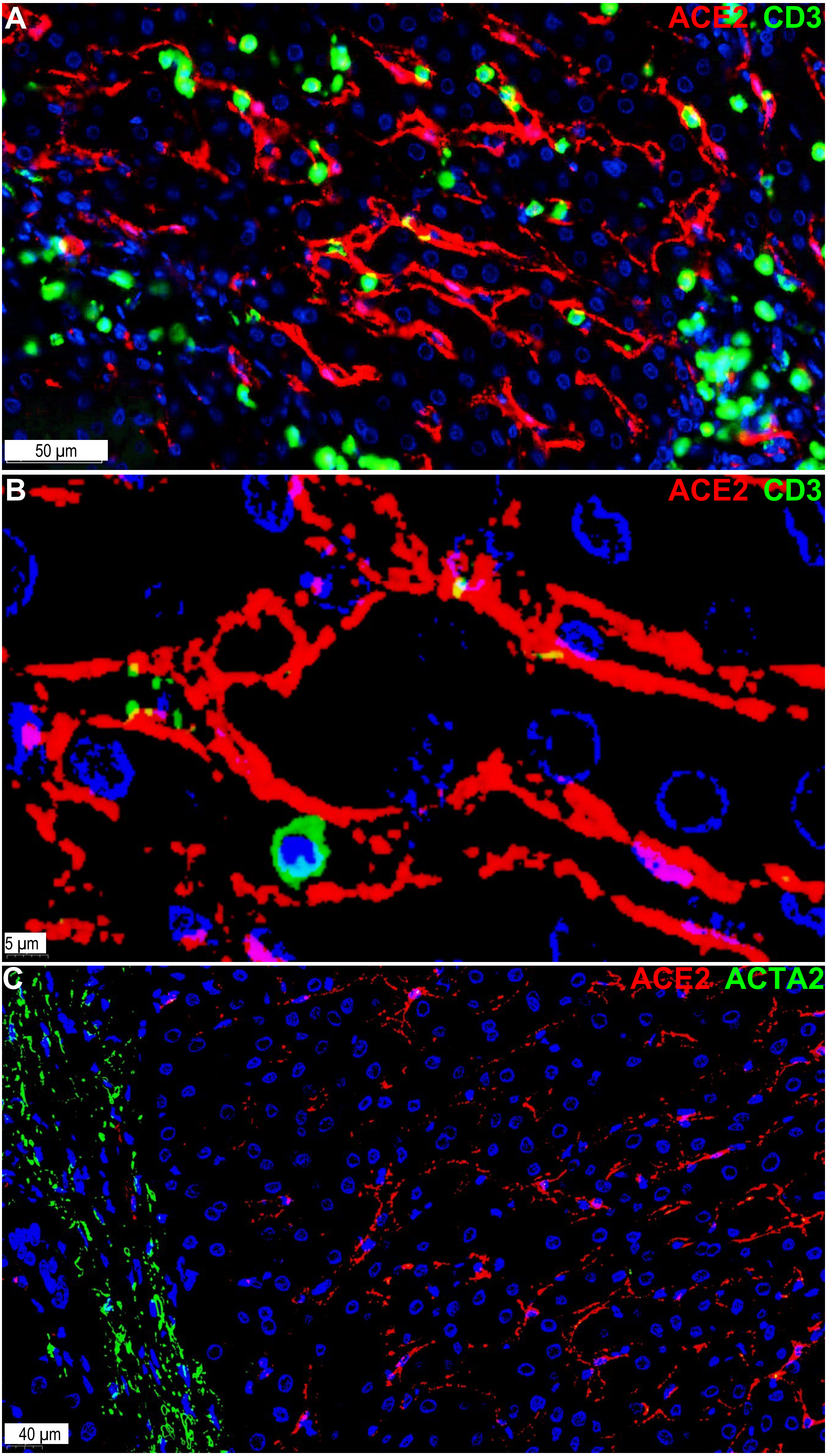


Cano et al., Fig. S5

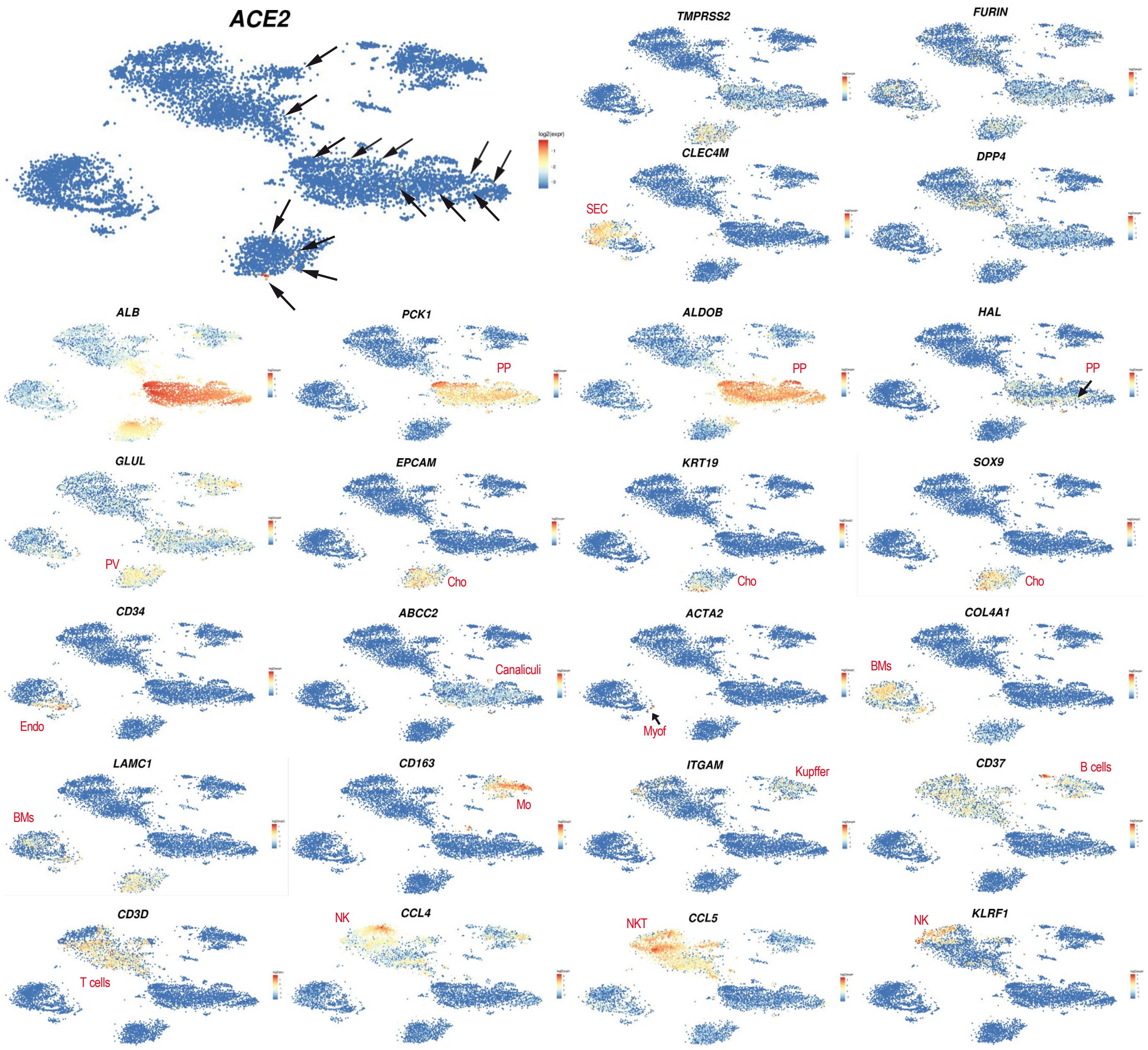


Cano et al., Fig. S6

# A

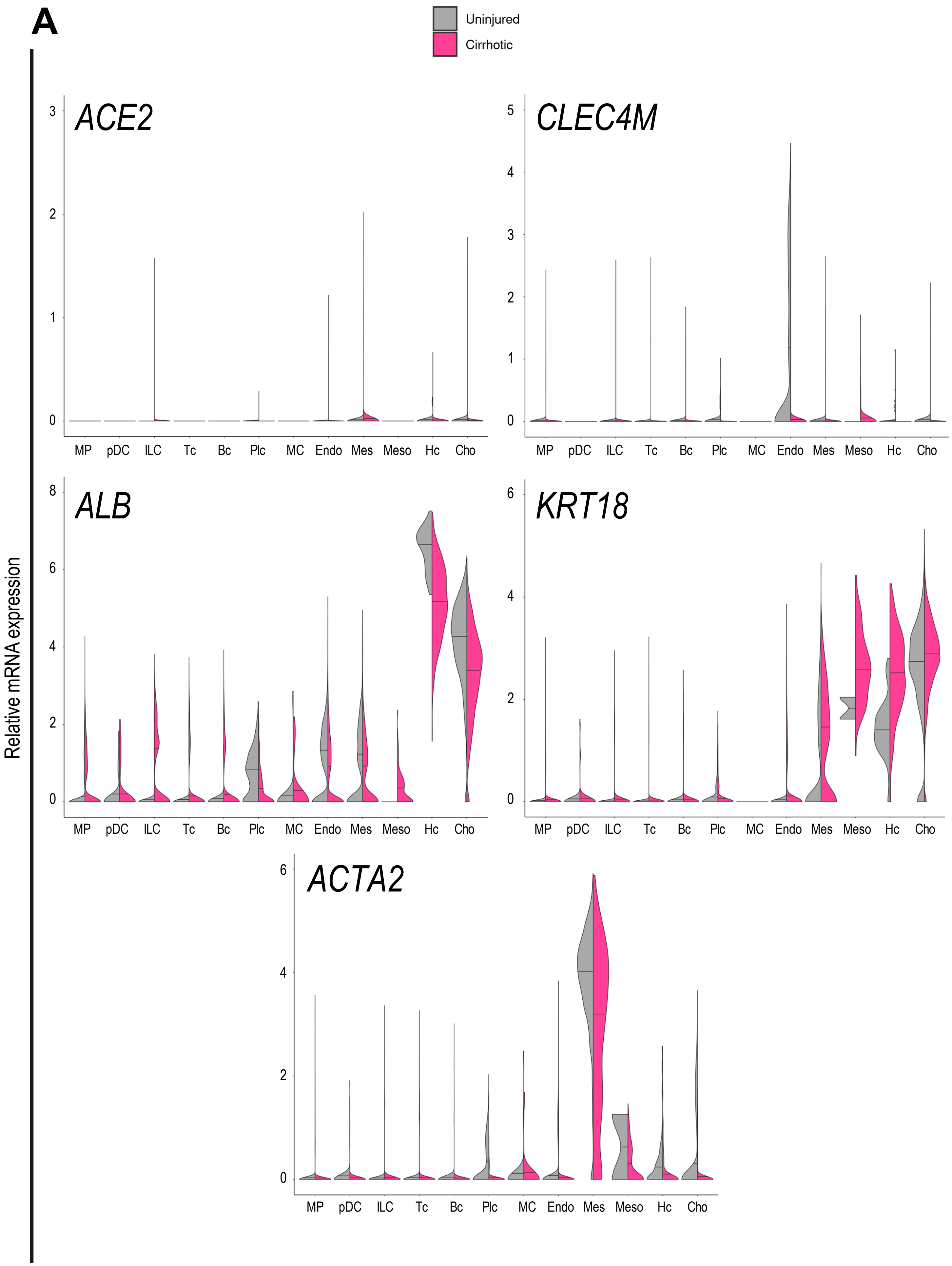


Cano et al., Fig. S7A

**B**

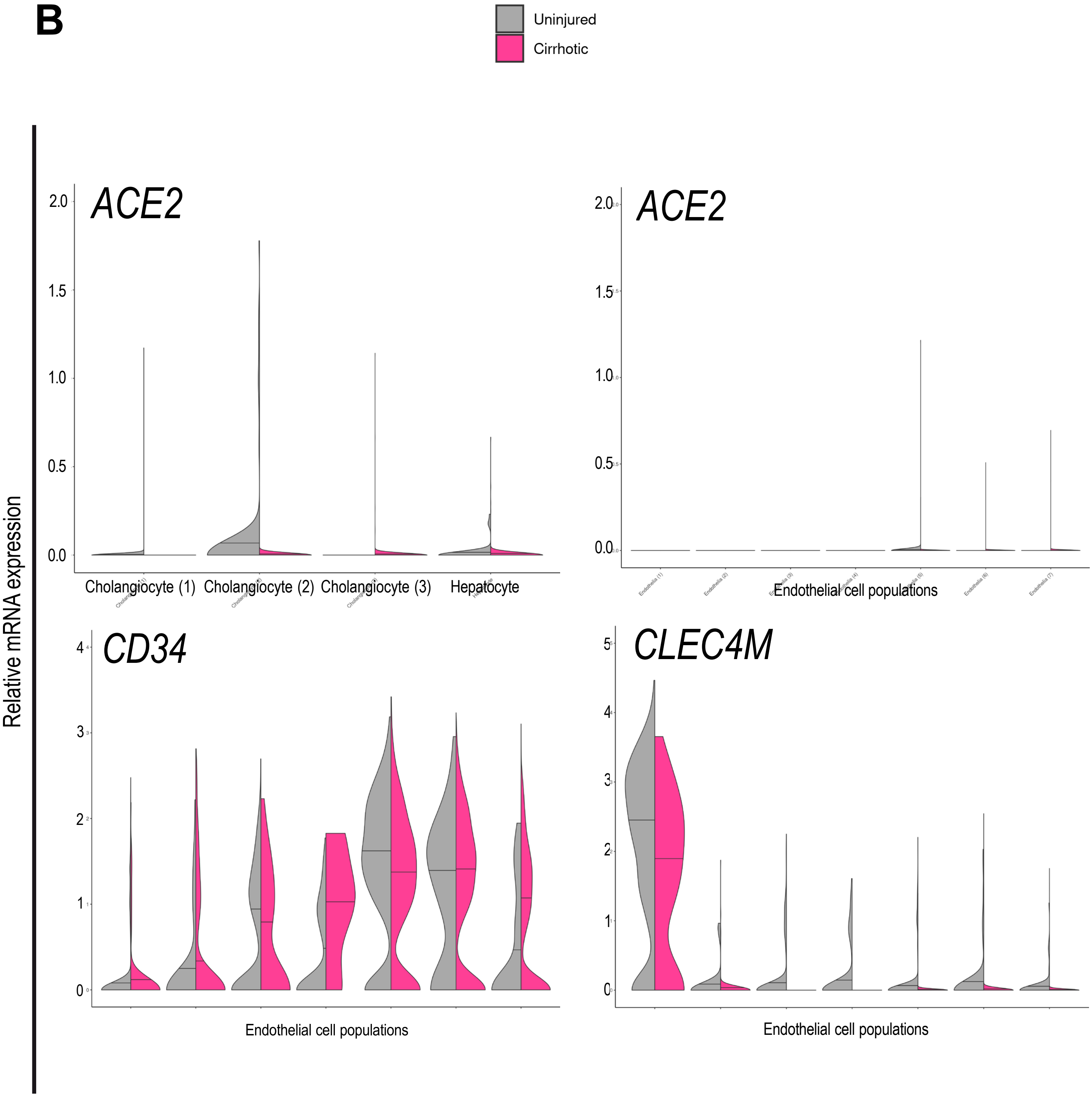


Cano et al., Fig. S7B

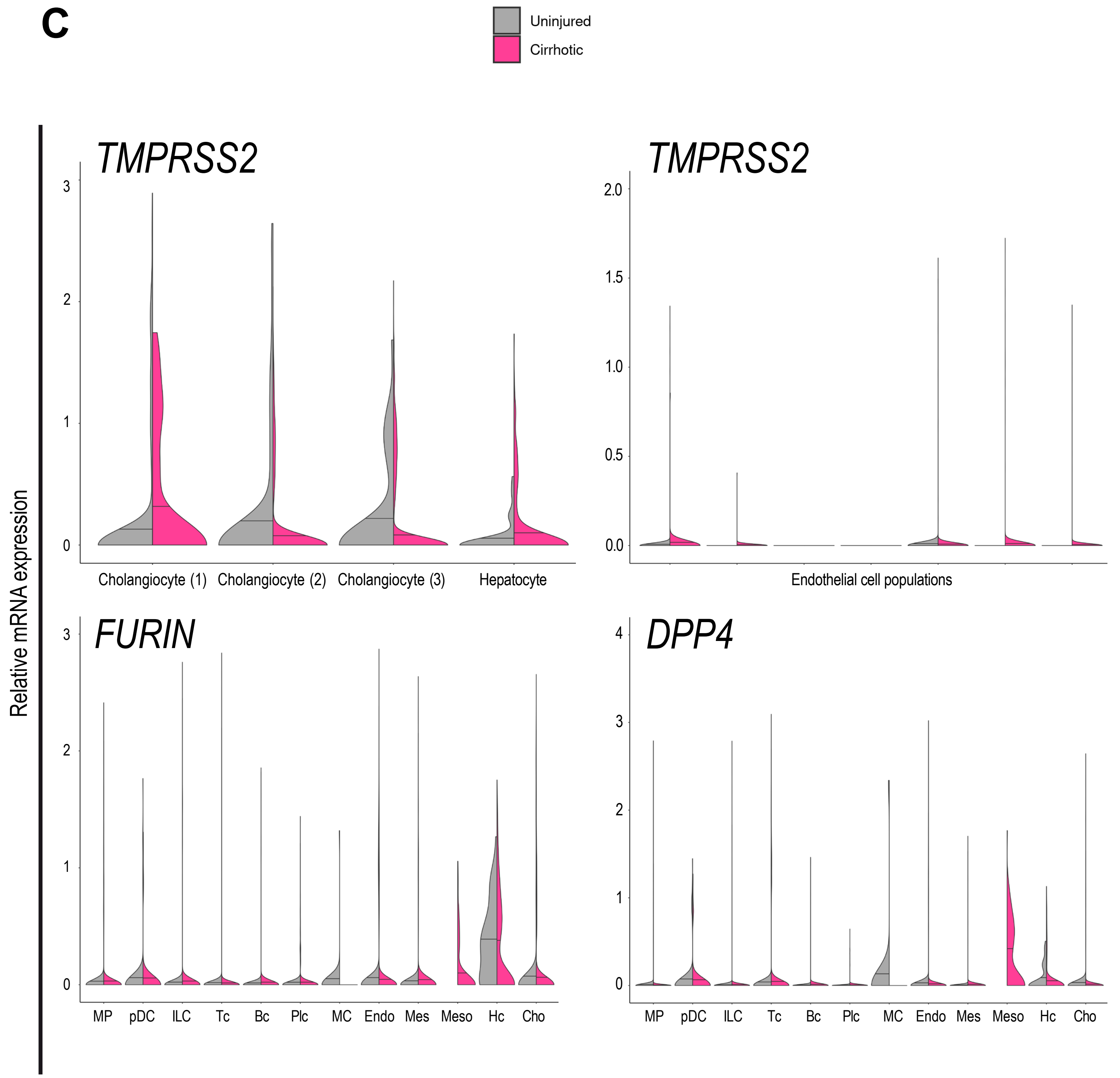


Cano et al., Fig. S7C

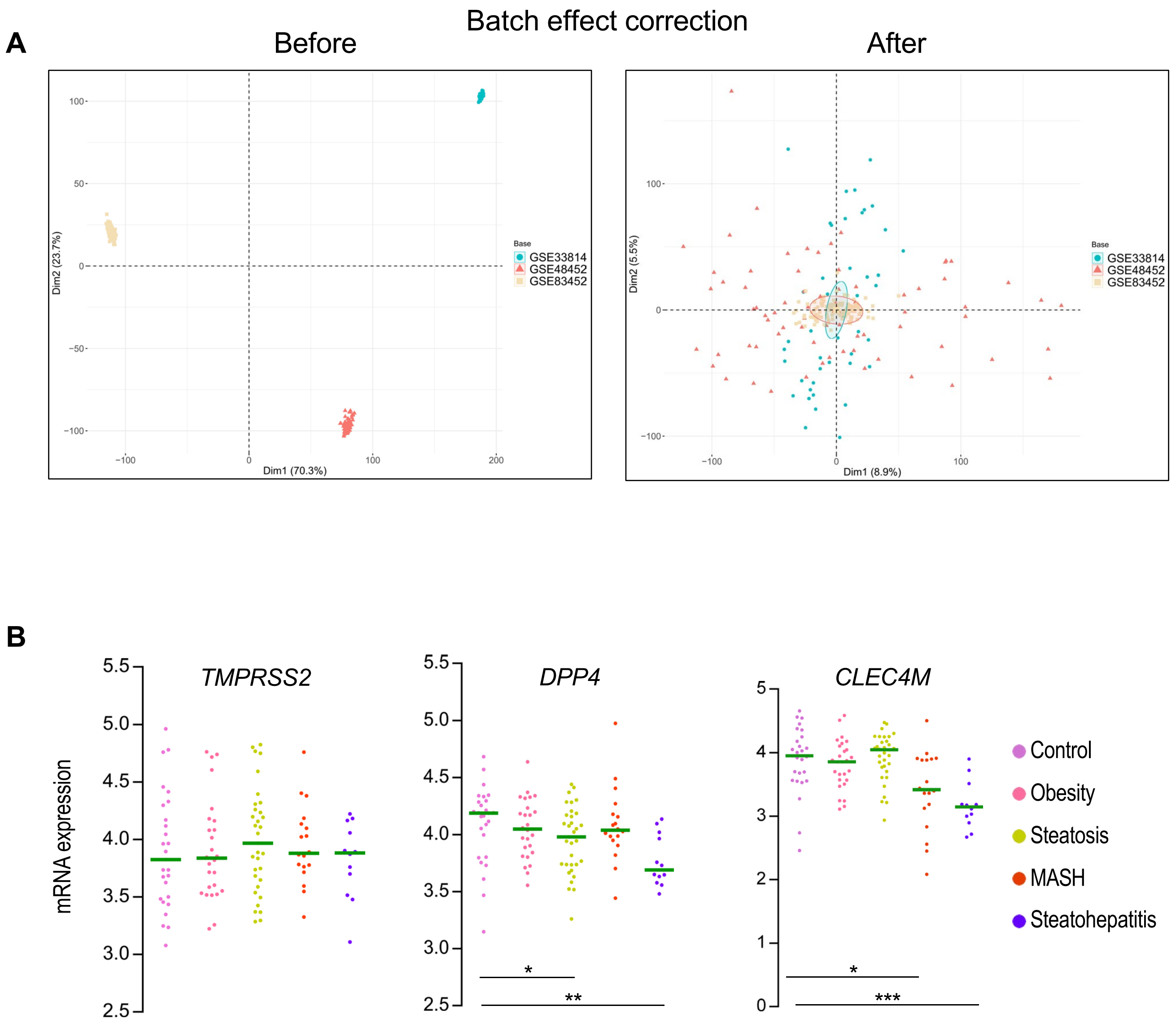


Cano et al., Fig. S8

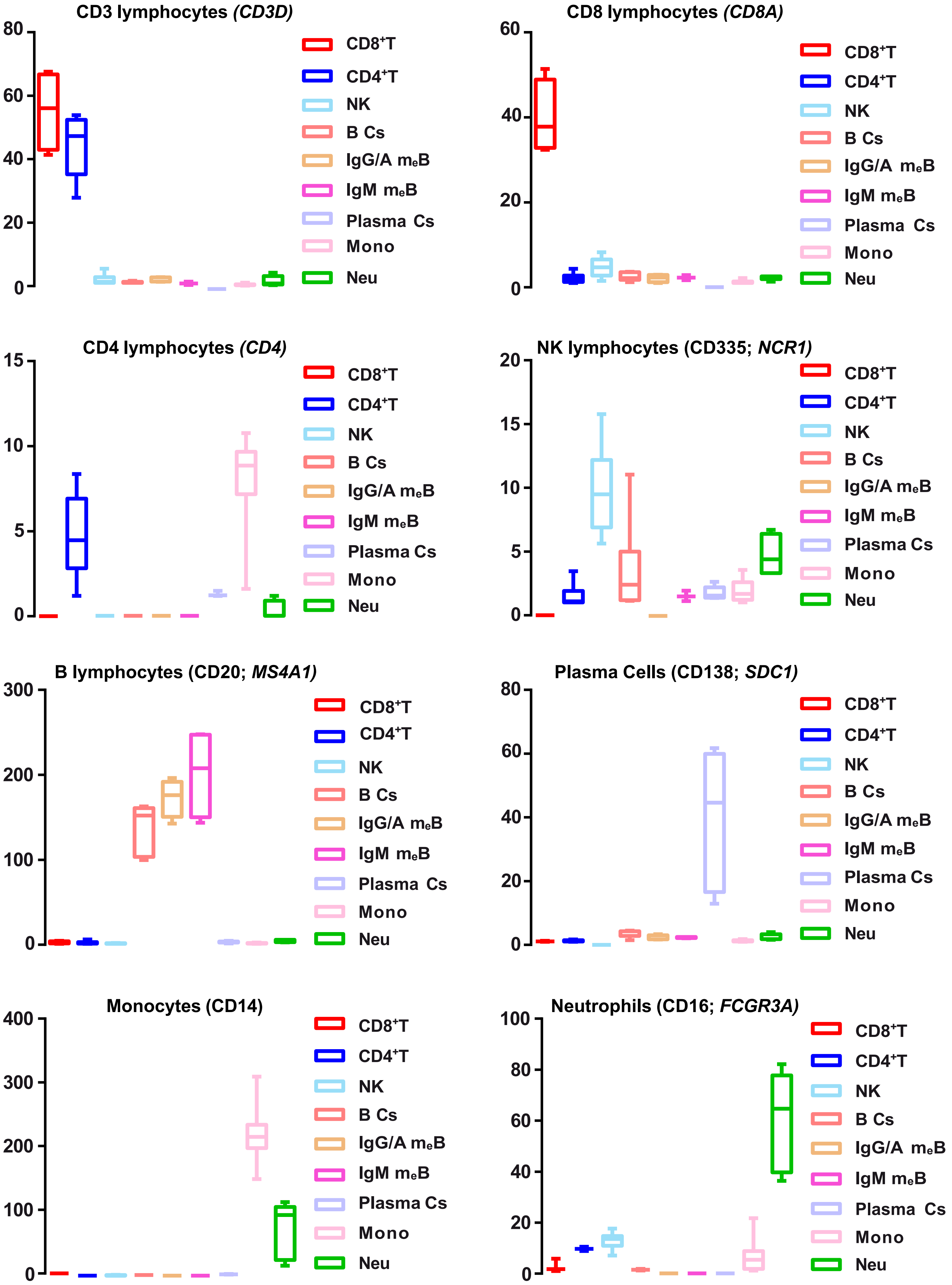


Cano et al., Fig. S9

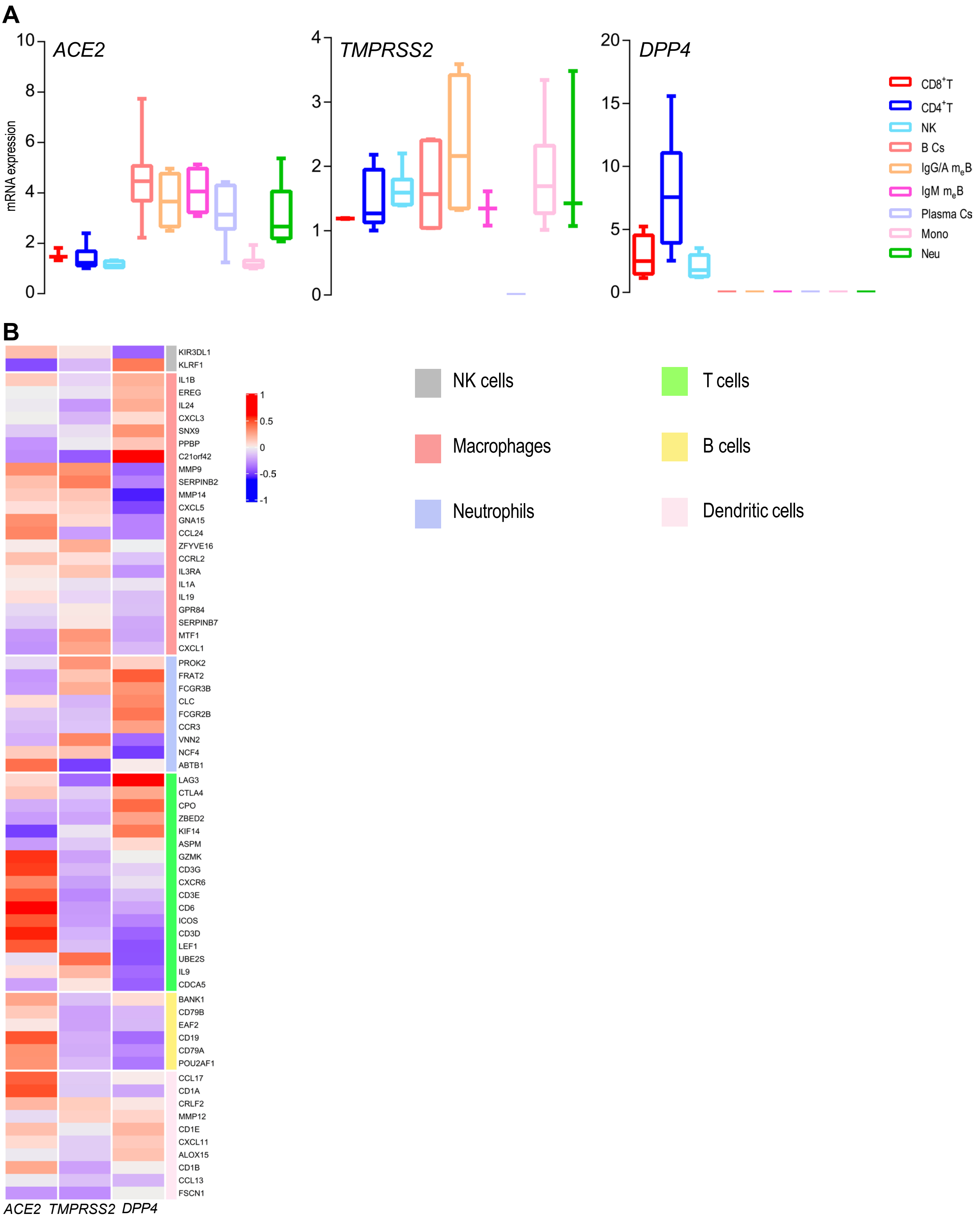


Cano et al., Fig. S10

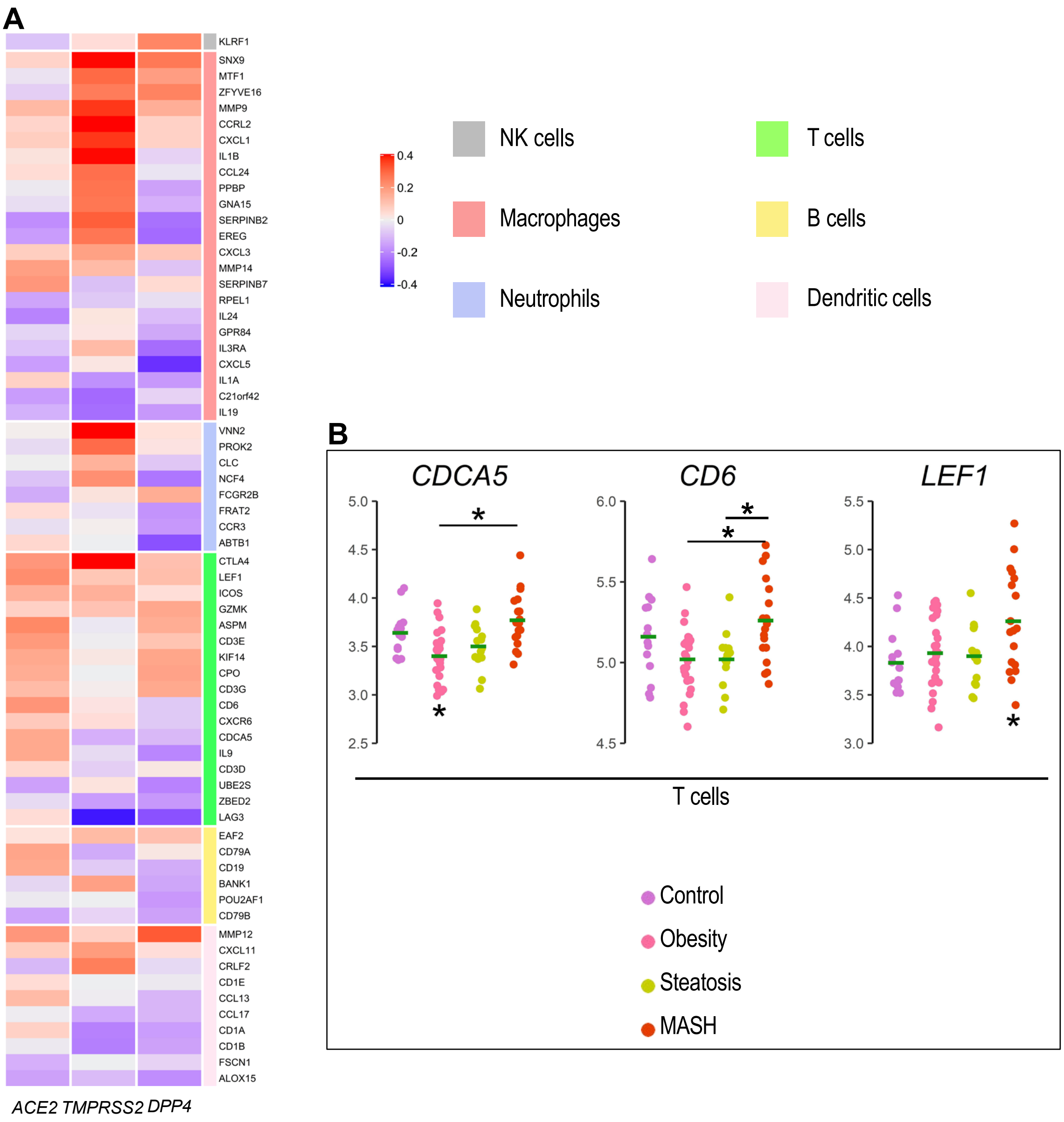


Cano et al., Fig. S11

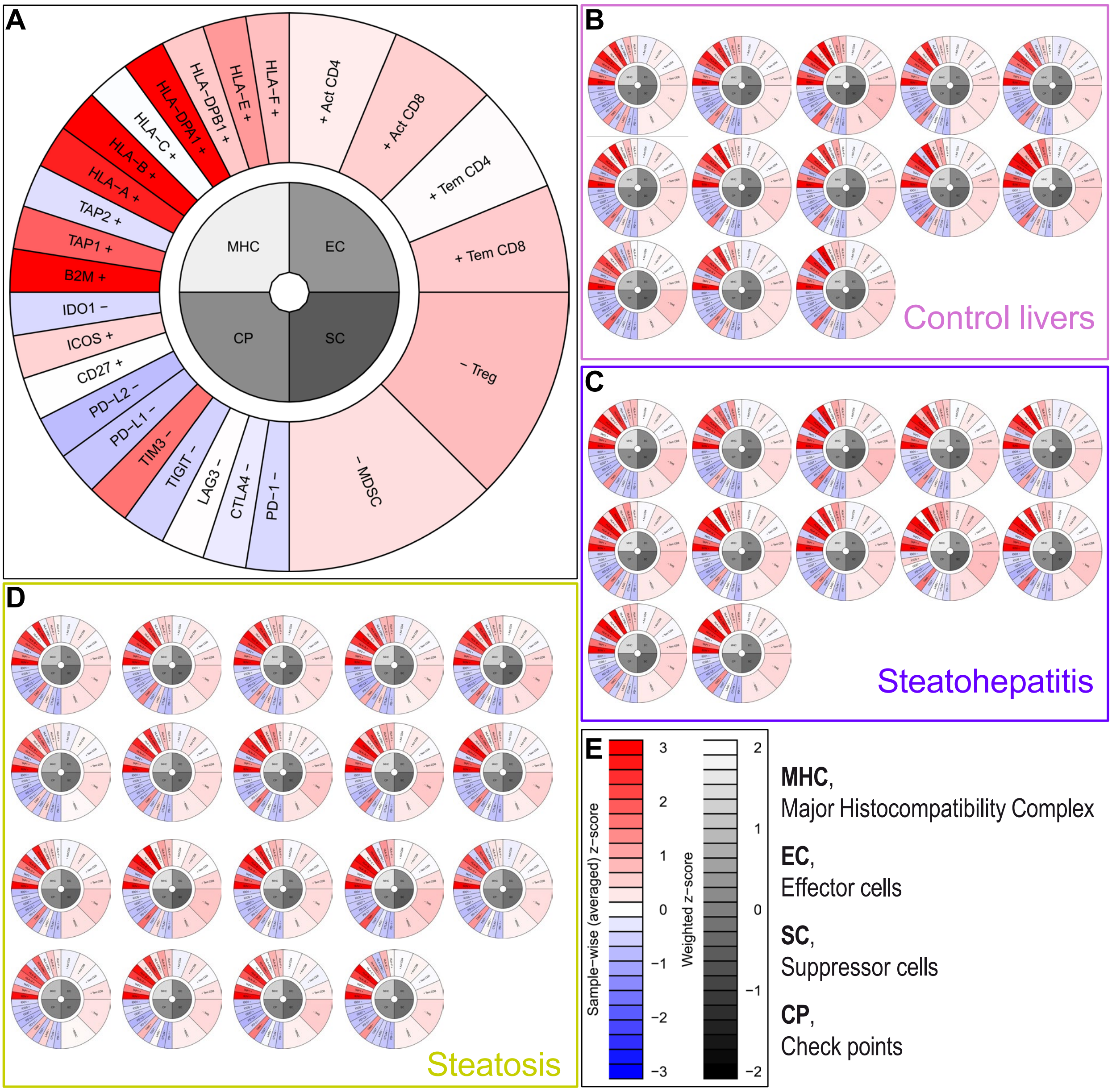


Cano et al., Fig. S12

# Eigengene Network

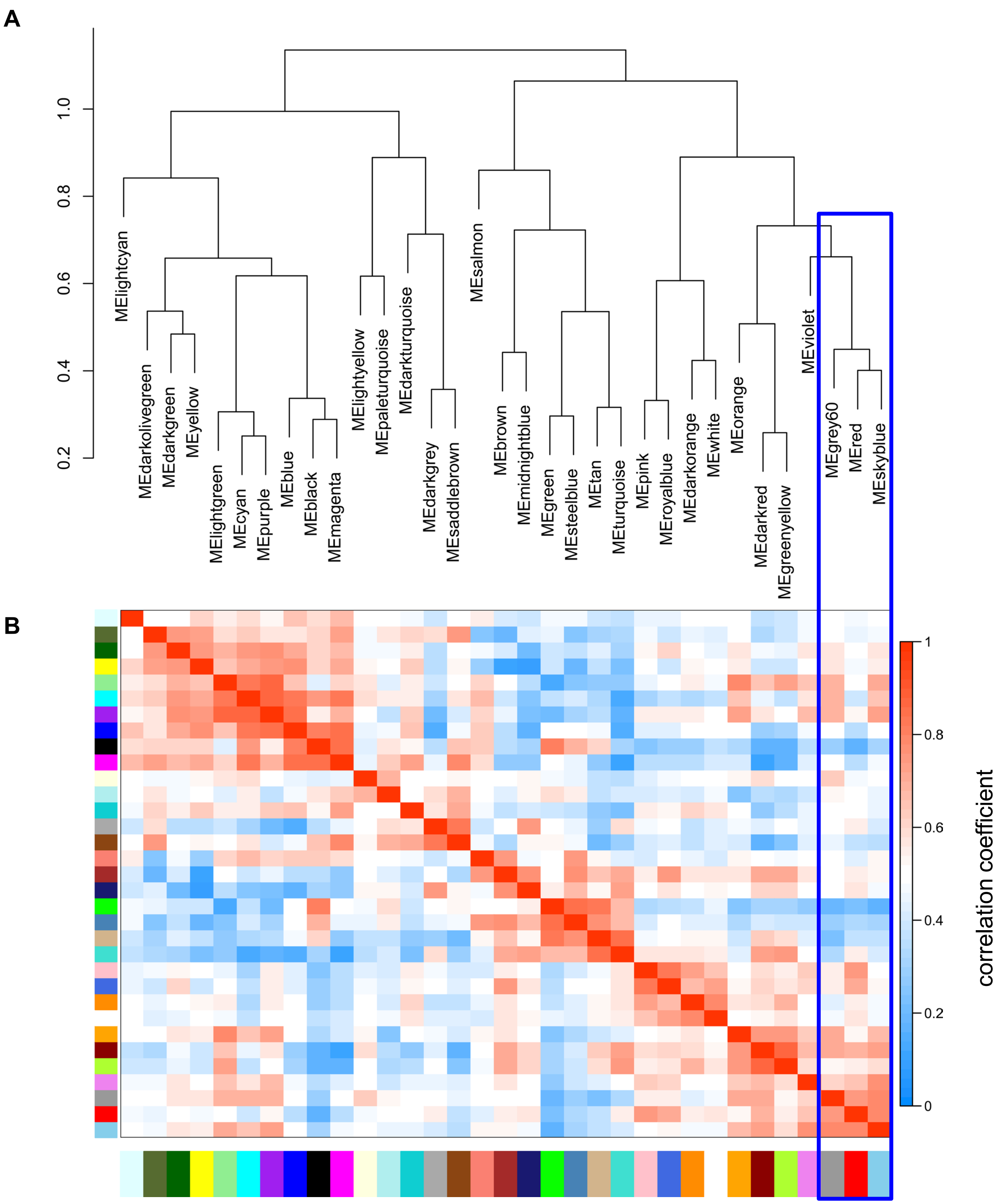


Cano et al., Fig. S13

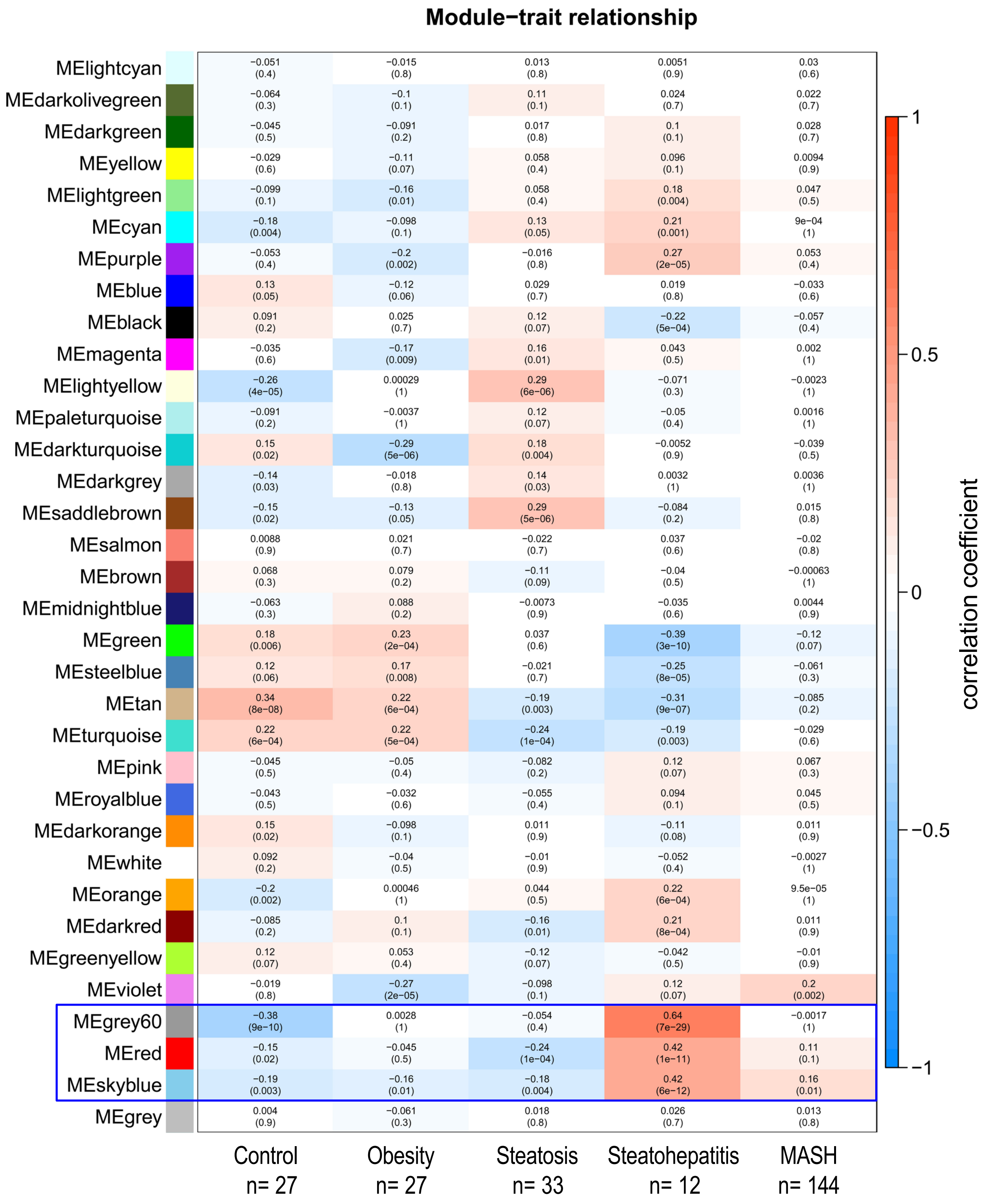


Cano et al., Fig. S14

Module Grey 60

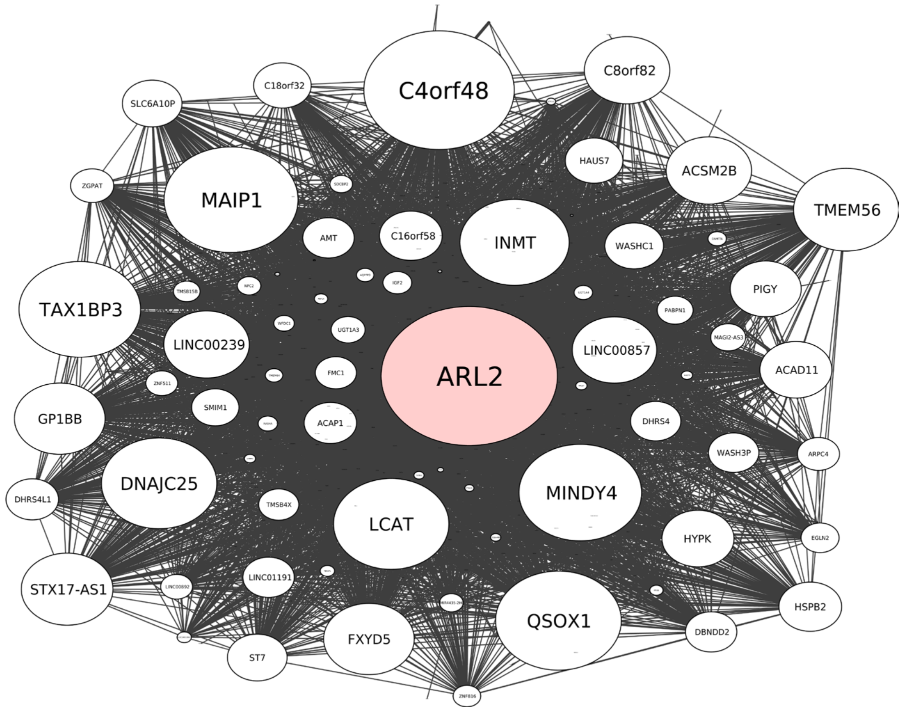


Module Red

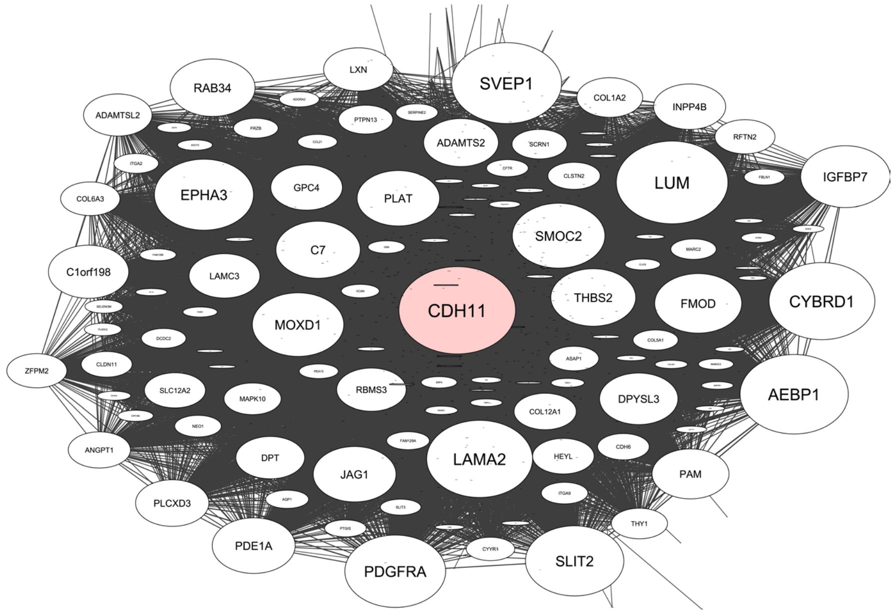


Module Skyblue

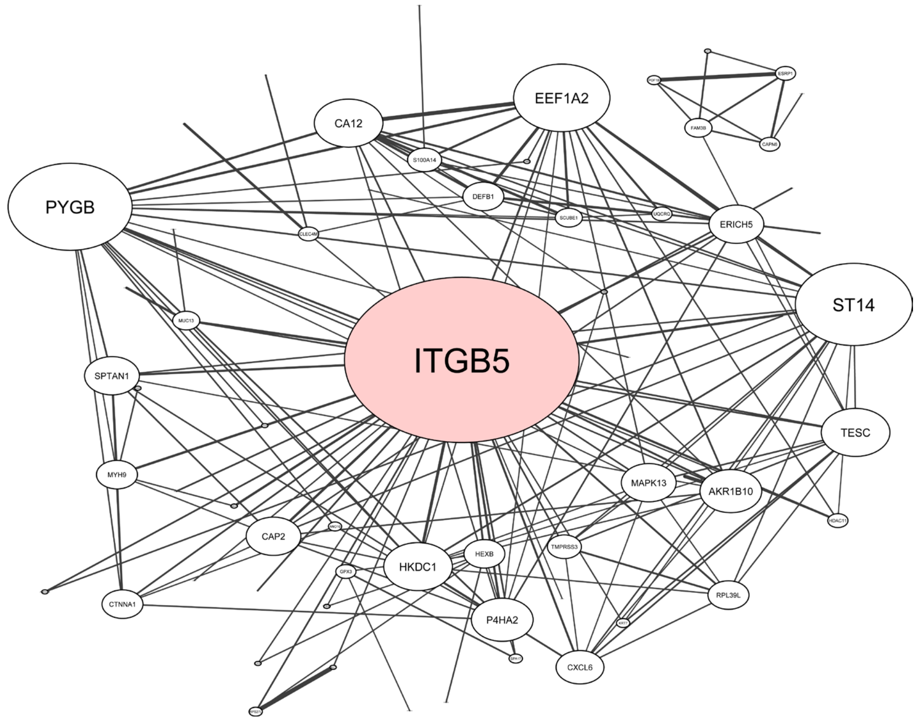


Cano et al., Fig. S15

## Module Grey 60

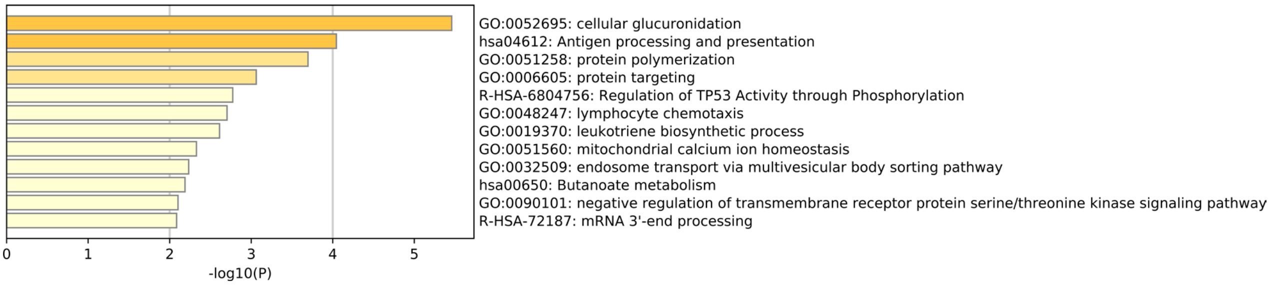


## Module Red

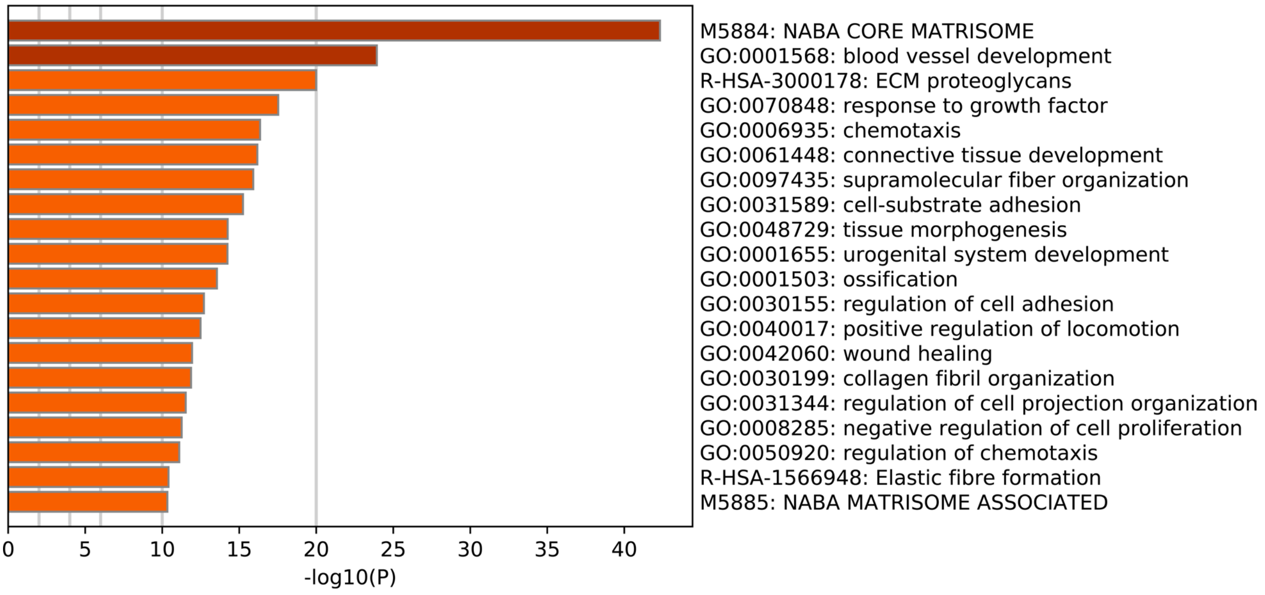


## Module Skyblue

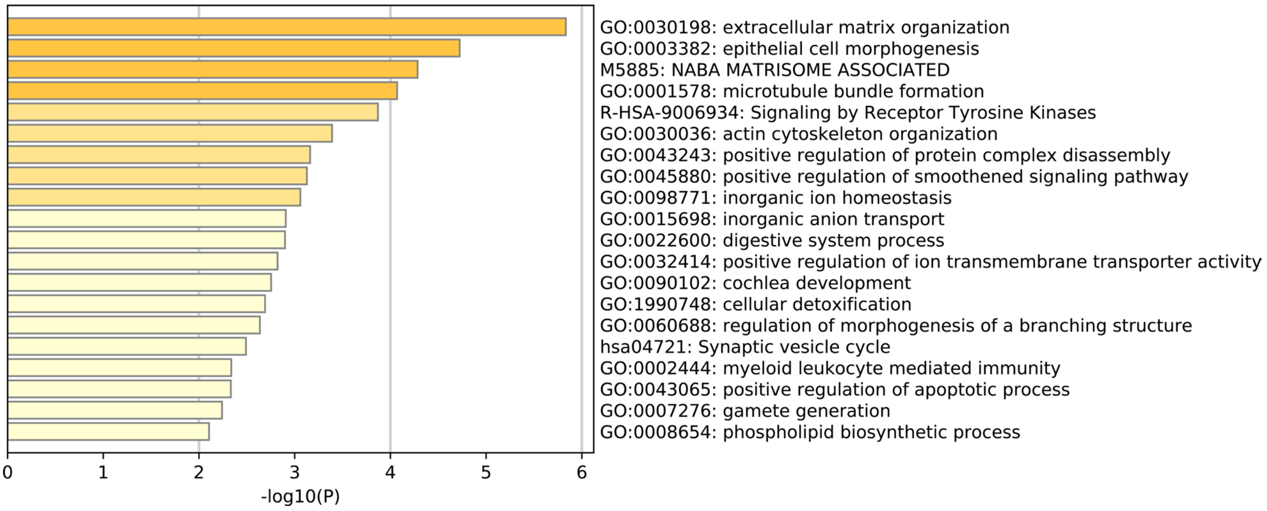


Cano et al., Fig. S16

**A** GSE28619 - Alcoholic Hepatitis (n = 15) *versus* normal (control, n= 7) liver.

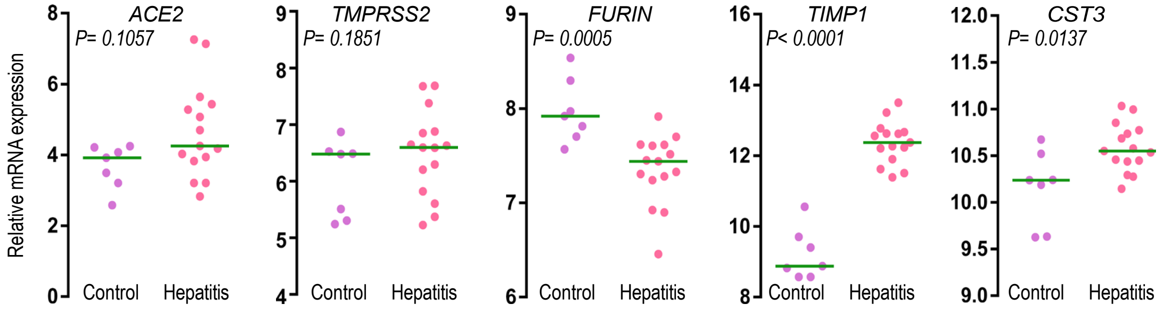


**B** GSE119117 - Longitudinal analysis of peripheral blood mononuclear cells in response to acute HCV infection.

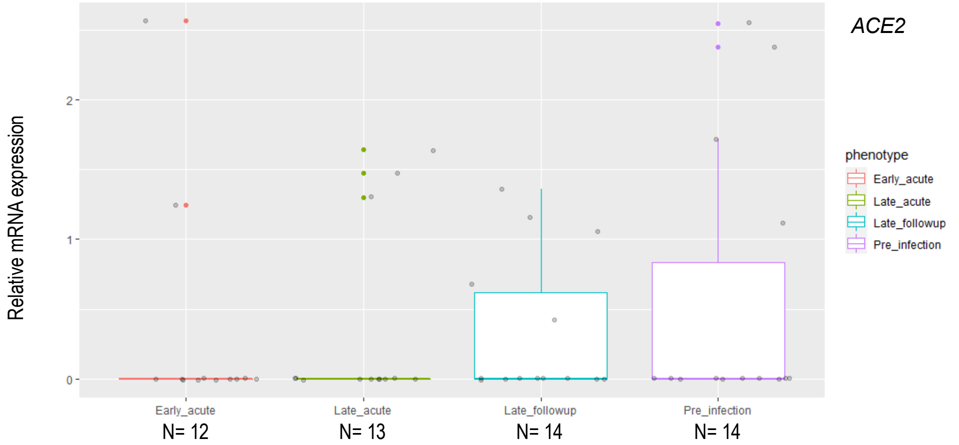


**C** GSE48445 - Effects of two forms of pegylated IFN$\alpha$ on liver gene expression in HCV patients.

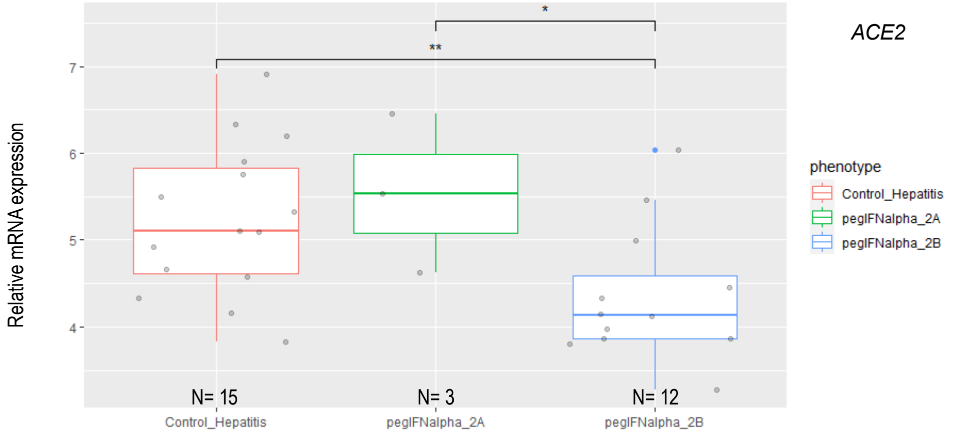


**D** GSE54747 - Chronic HBV liver treated with pegylated IFN$\alpha$2a and adefovir.

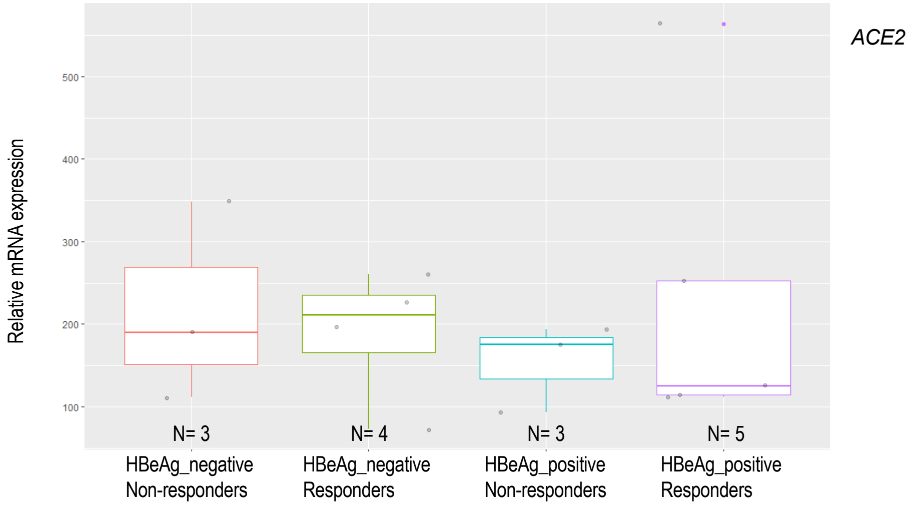


Cano et al., Fig. S17

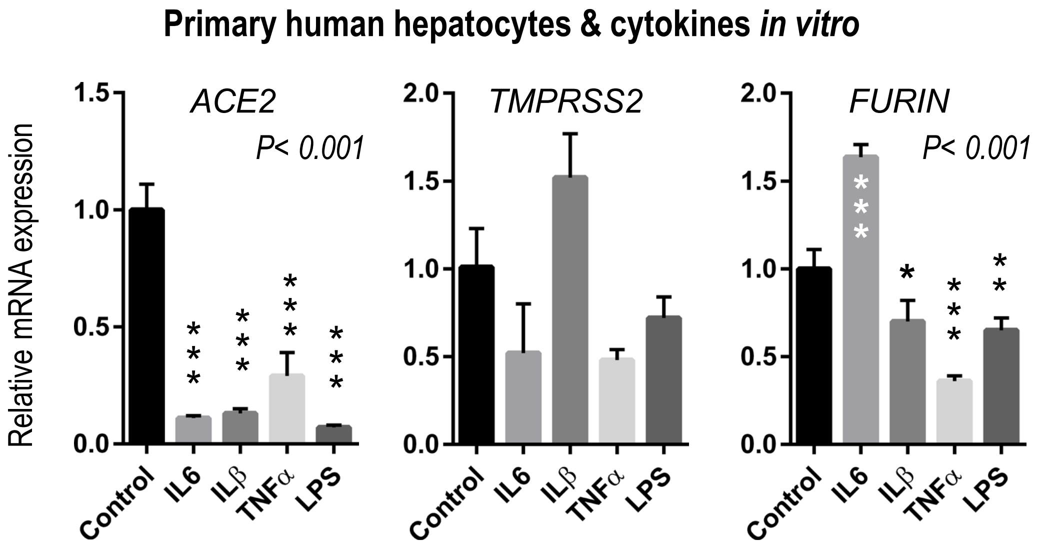


Cano et al., Fig. S18

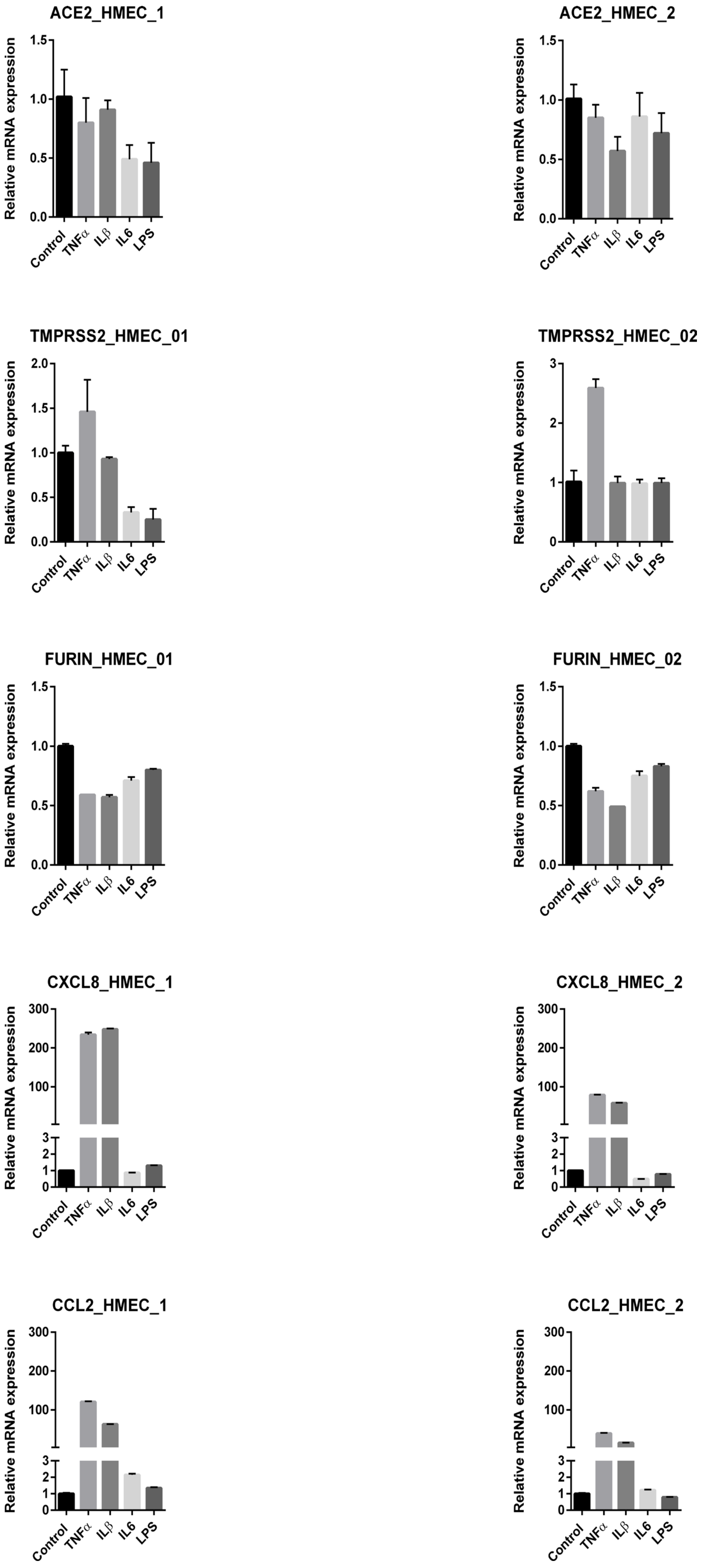


Cano et al., Fig. S19

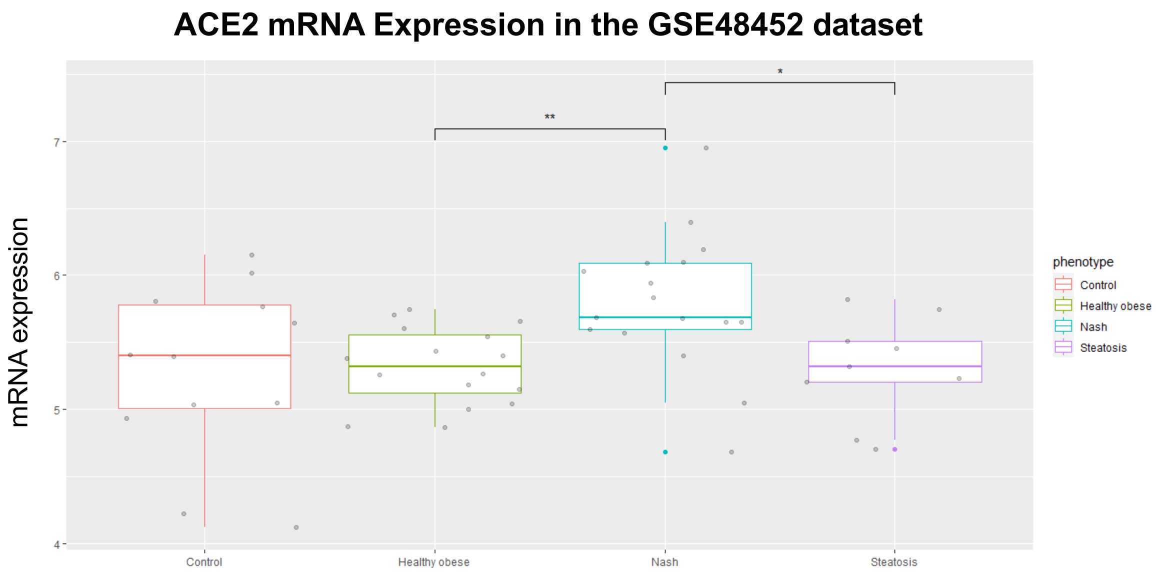


Cano et al., Fig. S20

**Supplementary Tables**

Table S4

| | GROUPS | | | | |
|---|---|---|---|---|---|
| GENE SYMBOL | Control | Obesity | Steatosis | MASH | Steatohepatitis |
| *ACE2* | 27 | 27 | 33 | 18 | 12 |
| *COL1A1* | 27 | 27 | 33 | 18 | 12 |
| *COL3A1* | 27 | 27 | 33 | 18 | 12 |
| *VCAN* | 27 | 27 | 33 | 18 | 12 |
| *COL4A1* | 27 | 27 | 33 | 144 | 12 |
| *LAMC1* | 27 | 27 | 33 | 144 | 12 |

Table S5

| Immunogenicity functional families | | | |
| --- | --- | --- | --- |
| **Major Histocompatibility Complex (MHC)** | **Effector Cells (EC)** | **Suppressor Cells (SC)** | **Check Points (CP)** |
| B2M, Beta-2-Microglobulin | Act CD4, Activated CD4 T cells | T reg, T regulatory cells | PD-1, Programmed cell death 1 |
| TAP1, Transporter 1, ATP Binding Cassette Subfamily B Member | Act CD8, Activated CD8 T cells | MDSC, Myeloid-derived suppressor cells | CTLA4, Cytotoxic T-Lymphocyte Associated Protein 4 |
| TAP2, Transporter 2, ATP Binding Cassette Subfamily B Member | Tem CD4, T effector memory CD4 cells | | LAG3, Lymphocyte Activating 3 |
| HLA-A, Major Histocompatibility Complex, Class I, A | Tem CD8, T effector memory CD8 cells | | TIGIT, T Cell Immunoreceptor With Ig And ITIM Domains |
| HLA-B, Major Histocompatibility Complex, Class I, B | | | TIM3, Hepatitis A Virus Cellular Receptor 2 (HAVCR2) |
| HLA-C, Major Histocompatibility Complex, Class I, C | | | PD-L1, Programmed Death Ligand 1 (CD274) |
| HLA-DPA1, Major Histocompatibility Complex, Class II, DP Alpha 1 | | | PD-L2, Programmed Cell Death 1 Ligand 2 (PDCD1LG2) |
| HLA-DPB1, Major Histocompatibility Complex, Class II, DP Beta 1 | | | CD27, T-Cell Activation Antigen CD27 |
| HLA-E, Major Histocompatibility Complex, Class I, E | | | ICOS, Inducible T Cell Costimulator |
| HLA-F, Major Histocompatibility Complex, Class I, F | | | IDO1, Indoleamine 2,3-Dioxygenase 1 |

Table S6

| Category | Control Livers (C) | | Steatosis (S) | | Steatohepatitis (ST) | | Kruskall Wallis | Dunn Test |
|---|---|---|---|---|---|---|---|---|
| | Mean | SD | Mean | SD | Mean | SD | | |
| **Treg** | 7,33 | 0,11 | 7,26 | 0,18 | 7,30 | 0,11 | 0,25 | |
| **MDSC** | 7,61 | 0,22 | 7,68 | 0,19 | 7,80 | 0,19 | 0,06 | |
| **Tem CD8** | 7,46 | 0,11 | 7,40 | 0,13 | 7,49 | 0,15 | 0,15 | |
| **Tem CD4** | 6,78 | 0,03 | 6,77 | 0,04 | 6,78 | 0,05 | 0,62 | |
| **Act CD8** | 7,21 | 0,09 | 7,19 | 0,11 | 7,31 | 0,14 | 0,08 | |
| **Act CD4** | 6,68 | 0,09 | 6,73 | 0,14 | 6,82 | 0,15 | **0,04** | **0.03 (C-ST)** |
| **IDO1** | 5,98 | 0,22 | 5,90 | 0,22 | 5,94 | 0,14 | 0,56 | |
| **ICOS** | 5,93 | 0,07 | 6,06 | 0,13 | 6,31 | 0,44 | **< 0.01** | **<0.01 (C-ST)**<br>**0.02 (C-S)**<br>**0.04 (S-ST)** |
| **CD27** | 5,78 | 0,09 | 5,75 | 0,14 | 5,98 | 0,29 | **< 0.01** | |
| **PD-L2** | 5,62 | 0,13 | 5,55 | 0,12 | 5,53 | 0,08 | 0,14 | |
| **PD-L1** | 5,78 | 0,09 | 5,85 | 0,16 | 5,77 | 0,06 | 0,27 | |
| **TIM3** | 9,28 | 0,36 | 8,92 | 0,56 | 8,89 | 0,44 | 0,08 | |
| **TIGIT** | 5,95 | 0,07 | 5,86 | 0,10 | 5,87 | 0,10 | **0,03** | **0.03 (C-S)** |
| **LAG3** | 7,28 | 0,52 | 7,11 | 0,47 | 6,87 | 0,41 | 0,11 | |
| **CTLA 4** | 5,94 | 0,13 | 5,84 | 0,14 | 5,90 | 0,21 | 0,21 | |
| **PD-1** | 5,69 | 0,05 | 5,68 | 0,08 | 5,71 | 0,16 | 0,91 | |
| **HLA-F** | 7,29 | 0,38 | 7,56 | 0,42 | 7,76 | 0,40 | **0,02** | **0.01 (C-ST)** |
| **HLA-E** | 8,17 | 0,50 | 8,10 | 0,46 | 8,39 | 0,37 | 0,18 | |
| **HLA-DPB1** | 6,80 | 0,43 | 6,74 | 0,45 | 6,94 | 0,47 | 0,41 | |
| **HLA-DPA1** | 10,90 | 0,69 | 10,52 | 0,80 | 11,27 | 0,75 | **0,02** | **0.01 (S-ST)** |
| **HLA-C** | 7,13 | 0,67 | 7,16 | 0,54 | 7,13 | 0,56 | 0,96 | |
| **HLA-B** | 11,15 | 0,42 | 11,36 | 0,69 | 11,90 | 0,48 | **< 0.01** | **<0.01 (C-ST)**<br>**0.04 (S-ST)** |
| **HLA-A** | 9,96 | 0,53 | 10,25 | 0,50 | 10,81 | 0,33 | **< 0.01** | **<0.01 (C-ST)**<br>**0.02 (S-ST)** |
| **TAP2** | 5,91 | 0,17 | 6,12 | 0,30 | 6,23 | 0,25 | **< 0.01** | **<0.01 (C-ST)** |
| **TAP1** | 9,16 | 0,45 | 9,32 | 0,46 | 9,61 | 0,43 | 0,07 | |
| **B2M** | 12,67 | 0,29 | 12,26 | 0,37 | 12,47 | 0,26 | **0,01** | **< 0.01(C-S)** |

Table S9

| | Immune cell subsets | | | | | | | | |
|---|---|---|---|---|---|---|---|---|---|
| **GENE SYMBOL** | **$CD8^+T$** | **$CD4^+T$** | **NK** | **B Cells** | **IgG/A $m_e$B** | **IgM $m_e$B** | **Plasma Cells** | **Monocytes** | **Neutrophiles** |
| *ACE2* | 3 | 10 | 4 | 7 | 4 | 4 | 7 | 13 | 5 |
| *TMPRSS2* | 2 | 11 | 6 | 7 | 4 | 2 | 0 | 22 | 3 |
| *DPP4* | 4 | 20 | 8 | 0 | 0 | 0 | 0 | 0 | 0 |
